# Silver-catalyzed anionic [3 + 2] cycloaddition of benzyl isocyanides with polyfluoroalkylchromones and pyrones: direct synthesis of 2-arylpyrroles

**DOI:** 10.3762/bjoc.22.103

**Published:** 2026-09-23

**Authors:** Ivan A Kochnev, Alexey Yu Barkov

**Affiliations:** 1 Institute of Natural Sciences and Mathematics, Ural Federal University, pr. Lenina 51, 620000 Yekaterinburg, Russian Federation

**Keywords:** benzyl isocyanides, [3 + 2] cycloaddition, polyfluoroalkyl, pyrroles, silver-catalyzed

## Abstract

A silver-catalyzed anionic [3 + 2] cycloaddition of benzyl isocyanides has been developed, providing direct one-pot access to 2-arylpyrroles – a privileged scaffold pervasive in medicinal chemistry and functional materials. Despite high demand, general one-step routes to this motif remain rare, and the synthetic potential of benzyl isocyanides in cycloaddition chemistry has been severely limited by their low reactivity. We herein show that the combination of AgOAc (10 mol %) and KO*t*-Bu efficiently promote the reaction of benzyl isocyanides with 2-polyfluoroalkylchromones and 6-(trifluoromethyl)-4*H*-pyrones, delivering 2-arylpyrroles in yields of up to 83% with complete chemo- and regioselectivity. The protocol is operationally simple and extends readily to chromones and pyrones lacking a trifluoromethyl group, demonstrating a broad substrate scope. This work establishes a practical catalytic entry to an important heterocyclic class and unlocks the underexplored utility of benzyl isocyanides in cycloaddition reactions.

## Introduction

**Figure 1:**
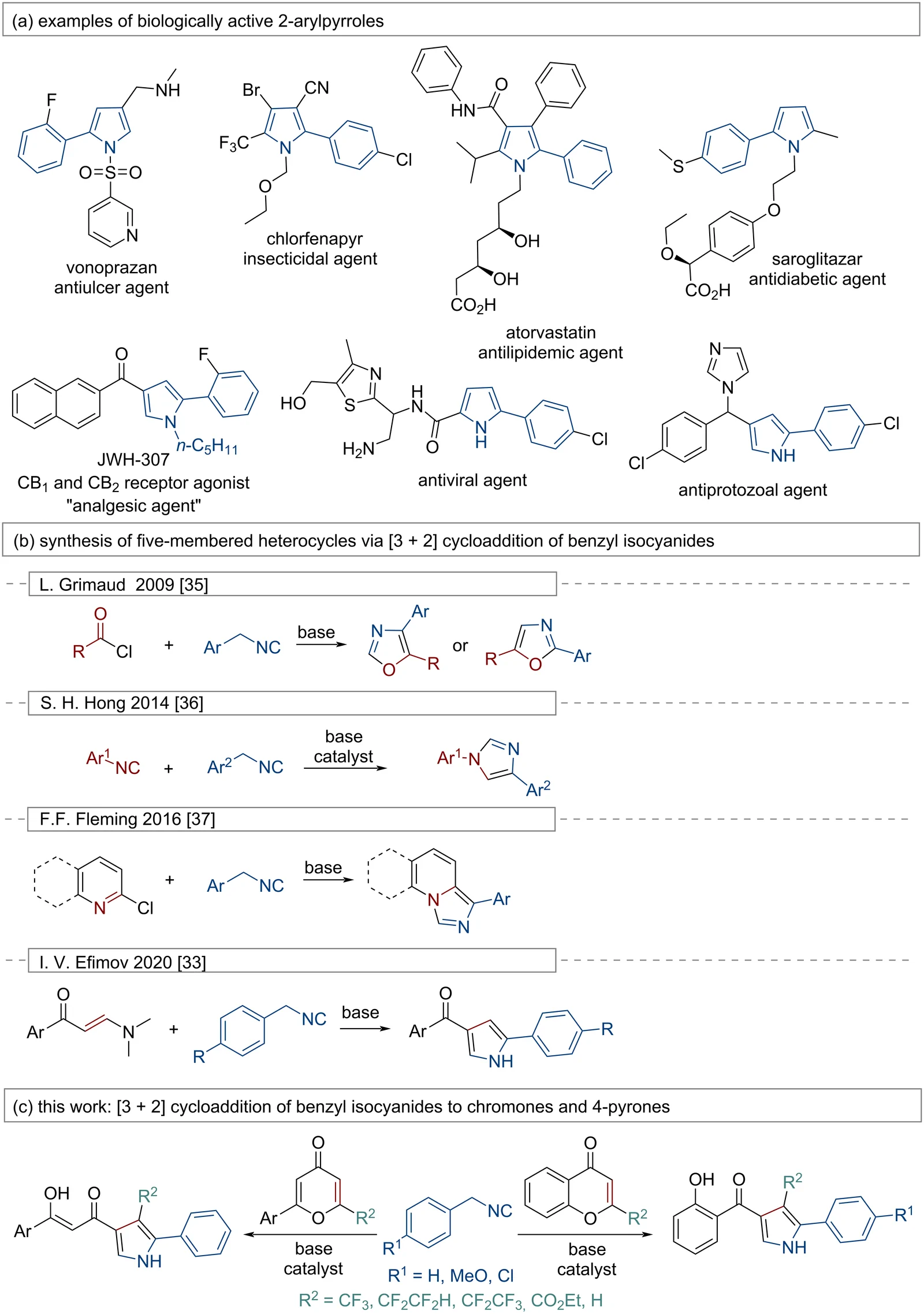
a) Examples of biologically active 2-arylpyrroles. b) Synthesis of five-membered heterocycles via [3 + 2] cycloaddition of benzyl isocyanides. c) This work: [3 + 2] cycloaddition of benzyl isocyanides to chromones and 4-pyrones.

The pyrrole scaffold is a valuable heterocyclic framework occurring in a diverse array of natural products, pharmaceutical substances, and functional materials [1–5]. Natural and synthetic molecules containing a functionalized pyrrole core are of great interest in medicinal chemistry due to their broad spectrum of biological and pharmaceutical activities [1–4,6–9] (Figure 1a). In particular, the 2-arylpyrrole framework is a ubiquitous pharmacophore found in bioactive compounds and commercial drugs, exhibiting multi-faced biological properties, such as analgesic, anti-ulcer and antiviral [10–16] (Figure 1a). Beyond their medicinal use, 2-arylpyrroles serve as versatile building blocks for the synthesis of heterocyclic ensembles and dyes [5,9,17–20]. In recent decades, many strategies for the synthesis of these compounds have been developed. The main approaches for the synthesis of 2-arylpyrroles include classical Paal–Knorr and Hantzsch methods, multicomponent reactions, transition-metal-catalyzed cyclization, palladium-catalyzed cross-coupling reactions and [3 + 2] cycloaddition reactions [9,21–34].

The [3 + 2] cycloaddition reaction is a straightforward one-pot strategy for the construction of functionalized pyrrole cores [38–39]. The distinctive features of this approach are great regioselectivity, a wide range of available substrates, and simple synthetic procedures [32–34,38–43]. One of the most employed approaches for the synthesis of pyrroles via [3 + 2] cycloaddition is the reaction of activated isocyanides with electron-deficient alkenes or alkynes. This synthetic methodology, first demonstrated by Barton and Zard, includes the [3 + 2] cycloaddition of isocyanoacetates with nitroalkenes under basic conditions [40–41]. Subsequently, the groups of Armin de Meijere, Lei, and Bi successfully applied this approach to other alkenes and alkynes bearing electron-withdrawing groups [42–46]. Moreover, in the past decade, the use of synthetic equivalents to activated alkynes, including enamines, chromones, aurones and allenes, has been demonstrated in the [3 + 2] isocyanide cycloaddition reaction [33–34,47–50]. However, previous works have primarily focused on the synthetic application of the most reactive methylene isocyanides, such as isocyanoacetates and tosylmethyl isocyanide (TosMIC). On the other hand, there are limited reports on the systematic use of benzyl isocyanides in [3 + 2] cycloaddition reactions [32–34,38–50]. This is likely due to a decreased reactivity stemming from the lower acidity of the benzyl proton compared to isocyanoacetates and TosMIC [33,35–37,42–46,51]. However, previous reports have demonstrated that the [3 + 2] cycloaddition of benzyl isocyanides offers a promising route to the synthesis of different five-membered heterocycles, including oxazoles, imidazoles, imidazopyridines, imidazoquinolines and 2-arylpyrroles [33,35–37] (Figure 1b). Consequently, developing efficient methodologies for the [3 + 2] cycloaddition of benzyl isocyanides is an important aspect of organic synthesis and medicinal chemistry.

2-Trifluoromethylchromones and 6-(trifluoromethyl)-4*H*-pyrones are versatile building blocks for the synthesis of carbo- and heterocyclic compounds [52–53]. They are of great interest for organic synthesis owing to their easy accessibility and multifaceted reactivity [52–60]. In general, 2-trifluoromethylchromones and 6-(trifluoromethyl)-4*H*-pyrones can act as masked carbonyl compounds, Michael acceptors, and substrates in [3 + 2] cycloaddition reactions [34,54–59]. The presence of a trifluoromethyl group increases the reactivity of these compounds, which makes them convenient reagents for the construction of trifluoromethyl-containing products [34,52–60]. It should be noted that trifluoromethylation of carbo- and heterocyclic compounds has significant effects on biological properties, including lipophilicity, metabolic stability, and bioavailability [60–61]. Therefore, the development of efficient protocols for the synthesis of new trifluoromethyl-containing heterocyclic compounds is a significant objective of medicinal and agricultural chemistry [58–61]. We already successfully employed 2-trifluoromethylchromones and 6-(trifluoromethyl)-4*H*-pyrones in the synthesis of trifluoromethyl-containing pyrroles via a metal-catalyzed [3 + 2] cycloaddition of ethyl isocyanoacetate [34].

Herein, we report a new method for the synthesis of trifluoromethylated 2-arylpyrroles via the silver-catalyzed [3 + 2] cycloaddition of benzyl isocyanides to 2-trifluoromethylchromones and 6-(trifluoromethyl)-4*H*-pyrones (Figure 1c).

## Results and Discussion

Initially, we investigated the model [3 + 2] cycloaddition reaction of benzyl isocyanide using different bases, solvents, and catalysts in accordance with literature data [33–34,42–50]. In the first experiments, it was shown that the desired product **3a** does not form when potassium carbonate, cesium carbonate, and DBU are used without a catalyst (Table 1, entries 1–3.). This is likely due to the insufficient basicity of these reagents for the deprotonation of benzyl isocyanide [33,35–37,51]. It is noteworthy that the use of transition metals such as copper or silver increases the reactivity of methyl isocyanide in [3 + 2] cycloaddition reactions [34,38–39,43,45,48]. In this work, copper iodide and silver acetate were used as catalysts, as they had demonstrated the highest efficiency in the cycloaddition of ethyl isocyanoacetate to chromones [34,44,50]. Nevertheless, the use of copper iodide and silver acetate in the presence of weak bases allowed for the production of 2-arylpyrrole **3a** with yields of 15–27% (Table 1, entries 4–7). Heating the reaction did slightly increase the yield of the product (Table 1, entries 8–10 and 23–26).

**Table 1:** Optimization of the reaction conditions.^a^

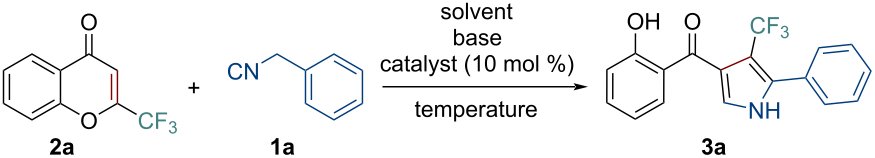					

entry	solvent	*T* [°C]	base	catalyst	yield [%]^b^

1^c^	MeCN	rt	K_2_CO_3_	–	ND^d^
2^c^	MeCN	rt	Cs_2_CO_3_	–	ND^d^
3^c^	MeCN	rt	DBU	–	ND^d^
4	MeCN	rt	K_2_CO_3_	CuI	15
5	MeCN	rt	K_2_CO_3_	AgOAc	22
6	MeCN	rt	Cs_2_CO_3_	CuI	27
7	MeCN	rt	DBU	CuI	25
8	MeCN	70	K_2_CO_3_	AgOAc	34
9	MeCN	70	Cs_2_CO_3_	AgOAc	41
10	MeCN	70	DBU	AgOAc	37
11^c^	MeCN	rt	KO*t*-Bu	–	32
12	MeCN	rt	KO*t*-Bu	AgOAc	40
13	MeCN	rt	KO*t*-Bu	CuI	61
14	MeCN	0–5	KO*t*-Bu	CuI	68
15	MeCN	0–5	KO*t*-Bu	AgOAc	60
16^c^	MeCN	0–5	KO*t*-Bu	–	35
17	MeCN	0–5	KO*t*-Bu	CuI	40
18	THF	0–5	KO*t*-Bu	CuI	30
19	THF	0–5	KO*t*-Bu	AgOAc	42
20	THF	–5 to –10	KO*t*-Bu	CuI	52
21	THF	–5 to –10	KO*t*-Bu	AgOAc	55
22	THF	0–5	NaH	CuI	25
23	DMF	70	K_2_CO_3_	AgOAc	37
24	DMF	70	Cs_2_CO_3_	AgOAc	47
25	DMF	70	DBU	AgOAc	43
26	DMF	100	DBU	AgOAc	30
27^b^	DMF	0–5	KO*t*-Bu	–	40
28	DMF	0–5	KO*t*-Bu	CuI	70
29	DMF	0–5	KO*t*-Bu	AgOAc	62
30	DMF	–5 to –10	KO*t*-Bu	CuI	78
31	DMF	–5 to –10	KO*t*-Bu	AgOAc	83
32	DMF	–15 to –20	KO*t*-Bu	AgOAc	35
33	DMF	0–5	NaH	CuI	65
34	DMF	0–5	NaH	AgOAc	67
35	DMF	–5 to –10	NaH	CuI	63
36	DMF	–5 to –10	NaH	AgOAc	68

^a^Reaction conditions: benzyl isocyanide (**1a**, 88 mg, 0.75 mmol, 1.5 equiv), chromone **2a** (107 mg, 0.5 mmol, 1.0 equiv), catalyst (0.05 mmol, 0.1 equiv, 10 mol %), base (1.0 mmol, 2.0 equiv), solvent (3 mL). ^b^All stated yields refer to isolated yields. ^c^The reaction was carried out without catalyst. ^d^Not detected.

Next, we studied the effect of strong non-nucleophilic bases in polar aprotic solvents. Indeed, when potassium *tert*-butoxide used without a catalyst, pyrrole **3a** is obtained in higher yields (Table 1, entries 11, 16, 27 vs entries 1–3). However, when using a catalyst, the yield of **3a** increased to 25–83% (Table 1, entries 12–15, 17–26 and 28–36). Moreover, the highest yield was achieved when using silver acetate as a catalyst (Table 1, entries 12, 15, 19, 21, 29, 31, 32, 34, 36). The increase in yield when using a catalyst is likely due to the formation of a complex that stabilizes the anion formed after the deprotonation of benzyl isocyanide.

It is noteworthy that the reaction temperature has a significant effect on the yield of pyrrole **3a**. For example, when the reaction is carried out at 0−5 °C, the yield of the product increases to 30–70% (Table 1, entries 14–19, 27–29, 33, 34). Moreover, further reduction of the reaction temperature from −5 to −10 °C leads to an increase in the yield of the product to 52–83% (Table 1, entries 20, 21, 30, 31, 35, 36). However, reducing the temperature from −15 to −20 °C leads to a decrease in the yield of pyrrole to 35% (Table 1, entry 32). The effect of the solvent was also demonstrated. The use of MeCN and THF results in comparable yields of the product (Table 1, entries 10–22). Nevertheless, the best yields of pyrrole were obtained in DMF (Table 1, entries 27–36). It should be noted that the [3 + 2] cycloaddition of benzyl isocyanide (**1a**) to chromone is characterized by great chemo- and regioselectivity, and pyrrole **3a** was the only product regardless of the solvent, base and catalyst used. Based on the reaction screening, the optimized conditions for the synthesis of 3-(trifluoromethyl)-2-phenylpyrrole **3a** were identified: 2.0 equiv of potassium *tert*-butoxide and 10 mol % of silver acetate in DMF at −5 to −10 °C for 4 hours.

**Scheme 1:**
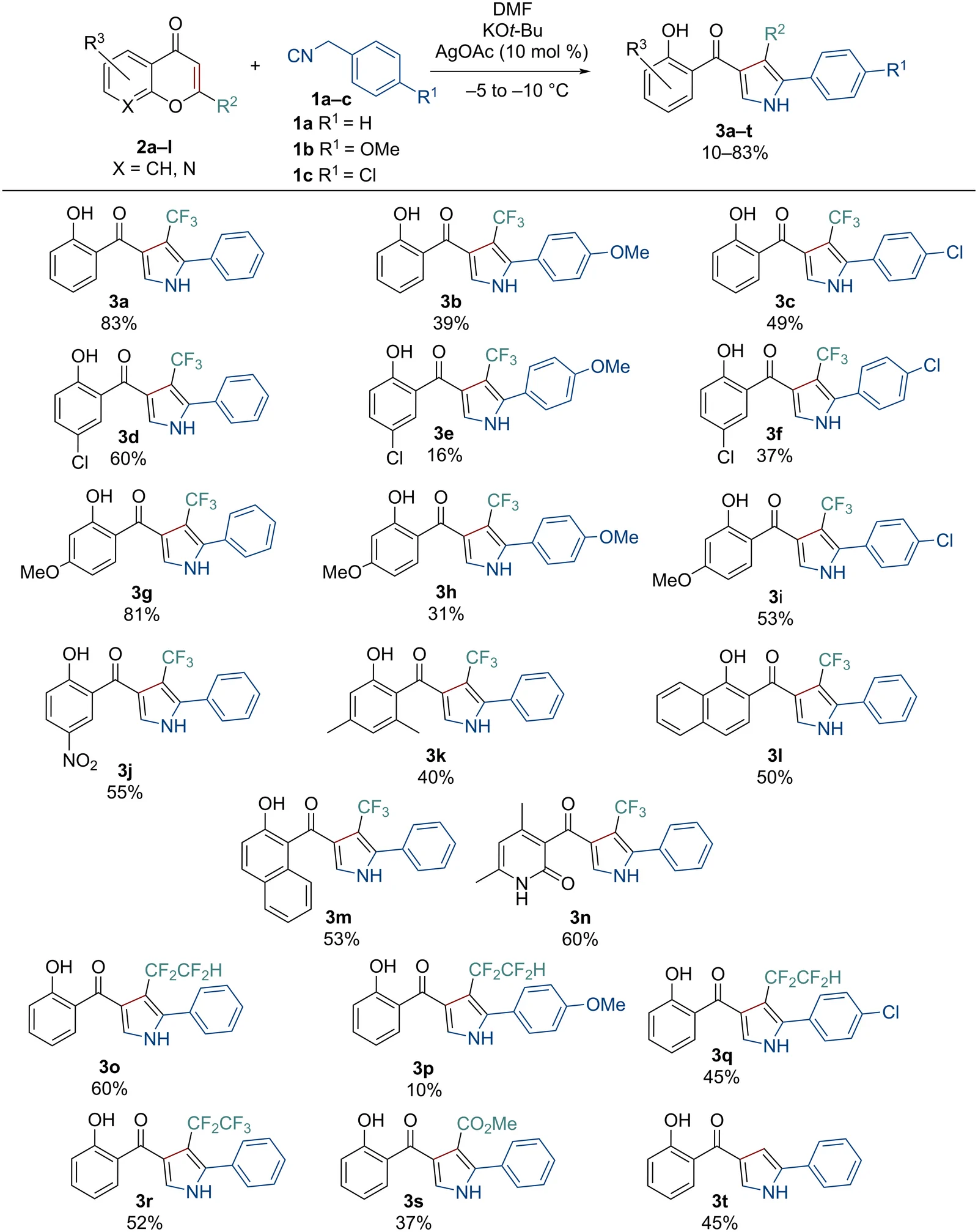
Cycloaddition of benzyl isocyanides **1a–c** with chromones **2a–l**. Reaction conditions: benzyl isocyanides **1a–c** (0.75 mmol, 1.5 equiv), chromones **2a–l** (0.5 mmol, 1.0 equiv), silver acetate (8 mg, 0.05 mmol, 0.1 equiv, 10 mol %), potassium *tert*-butoxide (112 mg, 1.0 mmol, 2.0 equiv), DMF (3 mL), −5 to −10 °C, 4 hours. All stated yields refer to isolated yields.

Next, we investigated the scope of the methodology by using various benzyl isocyanides **1a–c** and chromones **2a–l** (Scheme 1). A series of 2-arylpyrroles were obtained in up to 83% yield. The reaction proceeds with benzyl isocyanides possessing both an electron-donor substituent and a halogen atom. The presence of a donor substituent in the benzyl isocyanide leads to a significant decrease in the yields of pyrroles **3b**, **3e**, **3h** and **3p** to 10–39%. This is probably due to the decrease in the acidity of the methylene group of isocyanide **1b** due to the electron-donating effects of the methoxy group. A similar effect is observed for *p*-chlorobenzyl isocyanide used in the [3 + 2] cycloaddition reaction. However, the yields of pyrroles **3c, 3f**, **3i** and **3q** are 37–53%. The decrease in the yields of pyrroles **3c, 3f**, **3i** and **3q** is owing to the lower reactivity of the anion formed from isocyanide **1c** caused by the electronic acceptor effect of the chlorine atom.

**Scheme 2:**
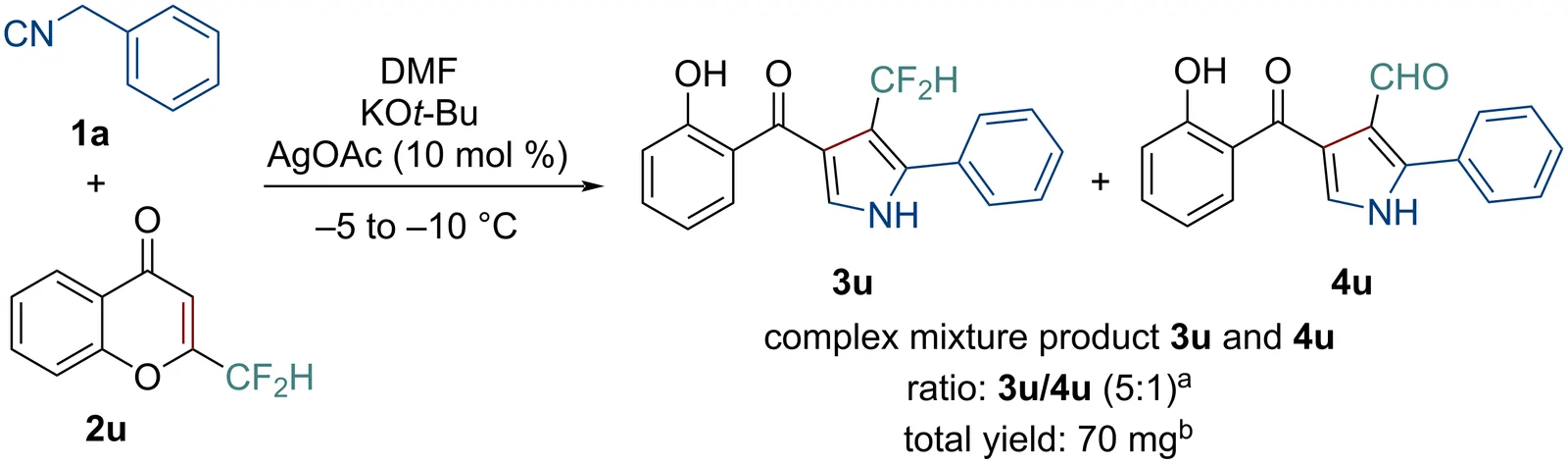
Reaction of benzyl isocyanide with chromone **2u**. Reaction conditions: benzyl isocyanide (**1a**, 88 mg.,0.75 mmol, 1.5 equiv), chromone **2u** (98 mg, 0.5 mmol, 1.0 equiv), silver acetate (8 mg, 0.05 mmol, 0.1 equiv, 10 mol %), potassium *tert*-butoxide (112 mg, 1.0 mmol, 2.0 equiv), DMF (3 mL), –5 to –10 °C, 4 hours. ^a^The structures and ratio of products **3u** and **4u** were determined by use of ^1^H, ^19^F, and ^13^C NMR spectra and HRMS. ^b^Stated yield refers to isolated yield.

**Figure 2:**
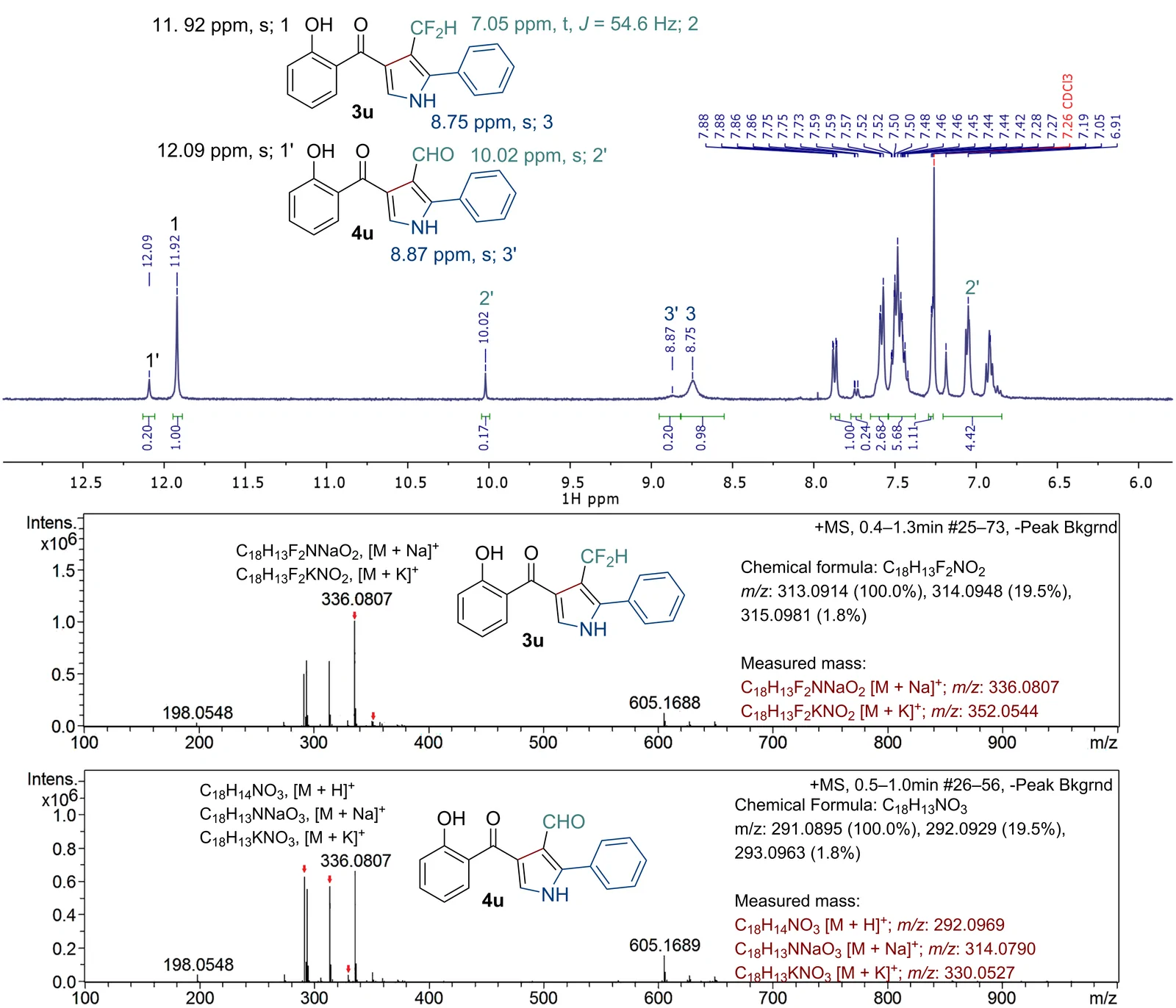
The image of ^1^H NMR spectrum and high-resolution mass spectra of a mixture of pyrroles **3u** and **4u**.

The effect of substituents in chromones has been demonstrated. Namely, the presence of a donor substituent in the aromatic core of the chromone has little effect on the yield of products **3a** and **3g**. On the other hand, the use of chromones containing a halogen atom or an acceptor substituent leads to decreased yields of pyrroles **3d–f** and **3j** to 16–60%. The same results were obtained when polysubstituted and fused chromones were used in the synthesis of 2-arylpyrroles **3k–m** with 40–53% yields. Moreover, the azachromone smoothly reacted with benzyl isocyanide to form the desired pyrrole **3n** with 60% yield. The effect of fluoroalkyl substituents has been demonstrated. When 2-trifluoromethylchromones were used, the highest yields were obtained due to their high reactivity. On the other hand, non-trifluoromethylated chromones were also successfully used in the synthesis of pyrroles **3s** and **3t**. It is noteworthy that when polyfluoroalkyl-containing chromones were used, the yields of pyrroles **3o–r** decreased to 10–60% (Scheme 1), which is likely due to steric hindrance and side elimination reactions that lead to the polymerization of intermediates and reaction products. In particular, when using 2-difluoromethylchromone (**2u)**, a complex mixture of difluoromethyl pyrrole **3u** and the hydrolysis product of the difluoromethyl group, pyrrole-3-carbaldehyde **4u**, was obtained (Scheme 2 and Supporting Information File 1). The structures and ratio of products **3u** and **4u** were determined based on literature data [62–63] and by using ^1^H, ^13^C, and ^19^F NMR spectra and high-resolution mass spectrometry (Figure 2 and Supporting Information File 1).

**Figure 3:**
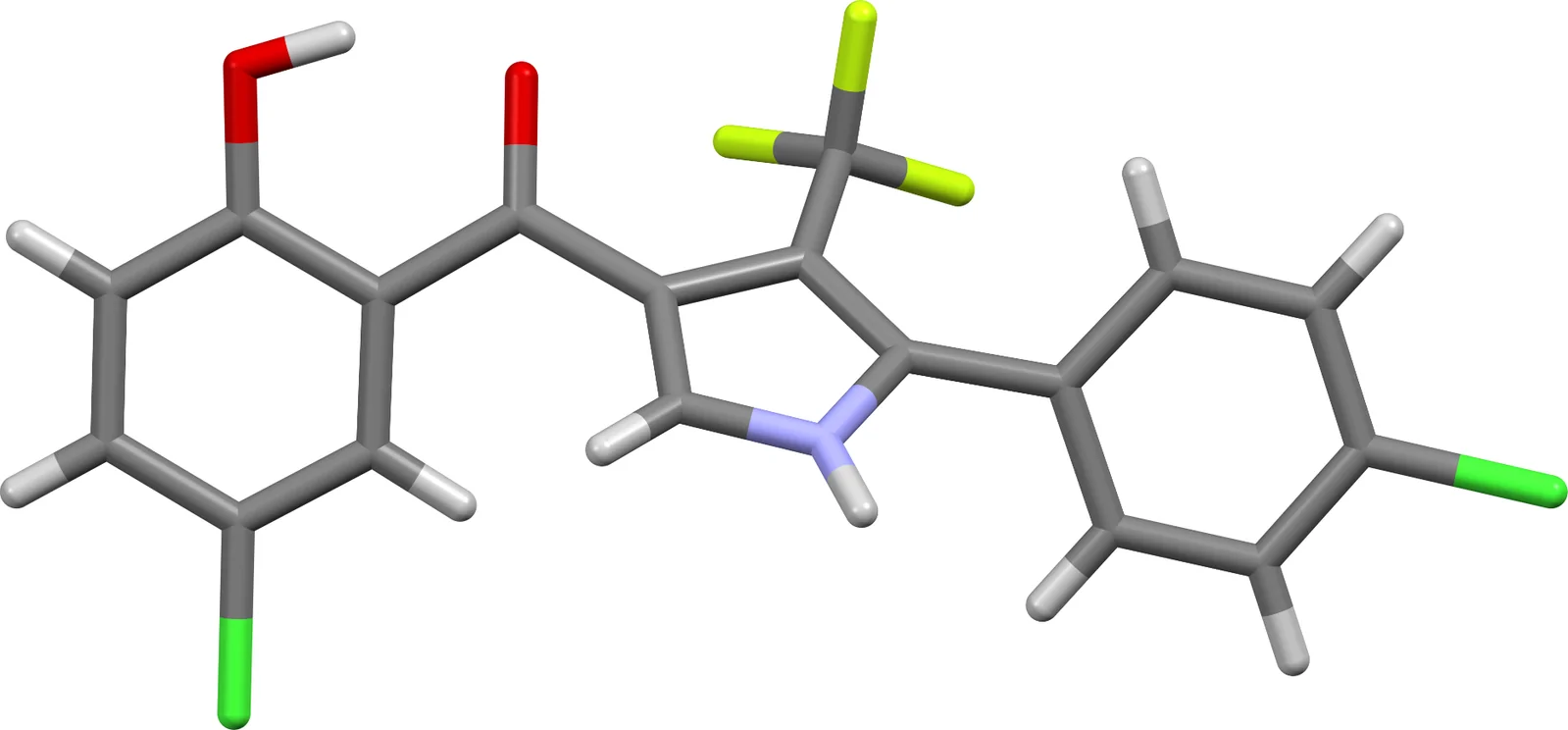
The X-ray crystal structure of compound **3f** (CCDC 2297092).

In the ^1^H NMR spectrum of the mixture of pyrroles **3u** and **4u**, two types of peaks were observed. Thus, the characteristic signals of difluoromethylpyrrole **3u** include the singlet of the phenolic proton 1 at 11.92 ppm, a broad singlet of the NH proton 3 of the pyrrole ring at 8.75 ppm and a triplet of the proton of the difluoromethyl group 2 with a spin-spin coupling constant *J* = 54.6 Hz (Figure 2 and Supporting Information File 1). Similarly, in the ^13^C NMR spectrum, characteristic signals of difluoromethylpyrrole **3u** were observed, such as a singlet of the carbonyl group at 192.3 ppm, a triplet of the C-3 atom of the pyrrole ring at 113.2 ppm with a spin-spin coupling constant *J* = 24.1 Hz and a characteristic triplet of the difluoromethyl group at 111.98 ppm with a spin-spin coupling constant *J* = 231.0 Hz. In addition, a single doublet of the difluoromethyl group of pyrrole **3u** was observed in the ^19^F NMR spectra (Supporting Information File 1). On the other hand, the structure of pyrrole-3-carbaldehyde **4u** was determined by using combined data from ^1^H and ^13^C NMR spectroscopy. Namely, the ^1^H NMR signals include a singlet of the phenolic proton 1’ at 12.09 ppm, a singlet of the aldehyde proton 2’ at 10.02 ppm and a broad singlet of the NH proton 3’ of the pyrrole ring at 8.87 ppm (Figure 2 and Supporting Information File 1). In the ^13^C NMR spectra, a second set of aromatic signals and two signals of carbonyl groups at 194.7 and 186.3 ppm were observed (Supporting Information File 1). These structural assignments of products **3u** and **4u** were confirmed by high-resolution mass spectrometry. Molecular ions of pyrroles **3u** and **4u** were successfully detected in the mass spectrum of the mixture (Figure 2 and Supporting Information File 1). Moreover, the structure of 2-aryl-3-polyfluoroalkylpyrroles **3a–u** was unambiguously established by X-ray single-crystal analysis (CCDC 2297092. Figure 3 and Supporting Information File 1).

**Scheme 3:**
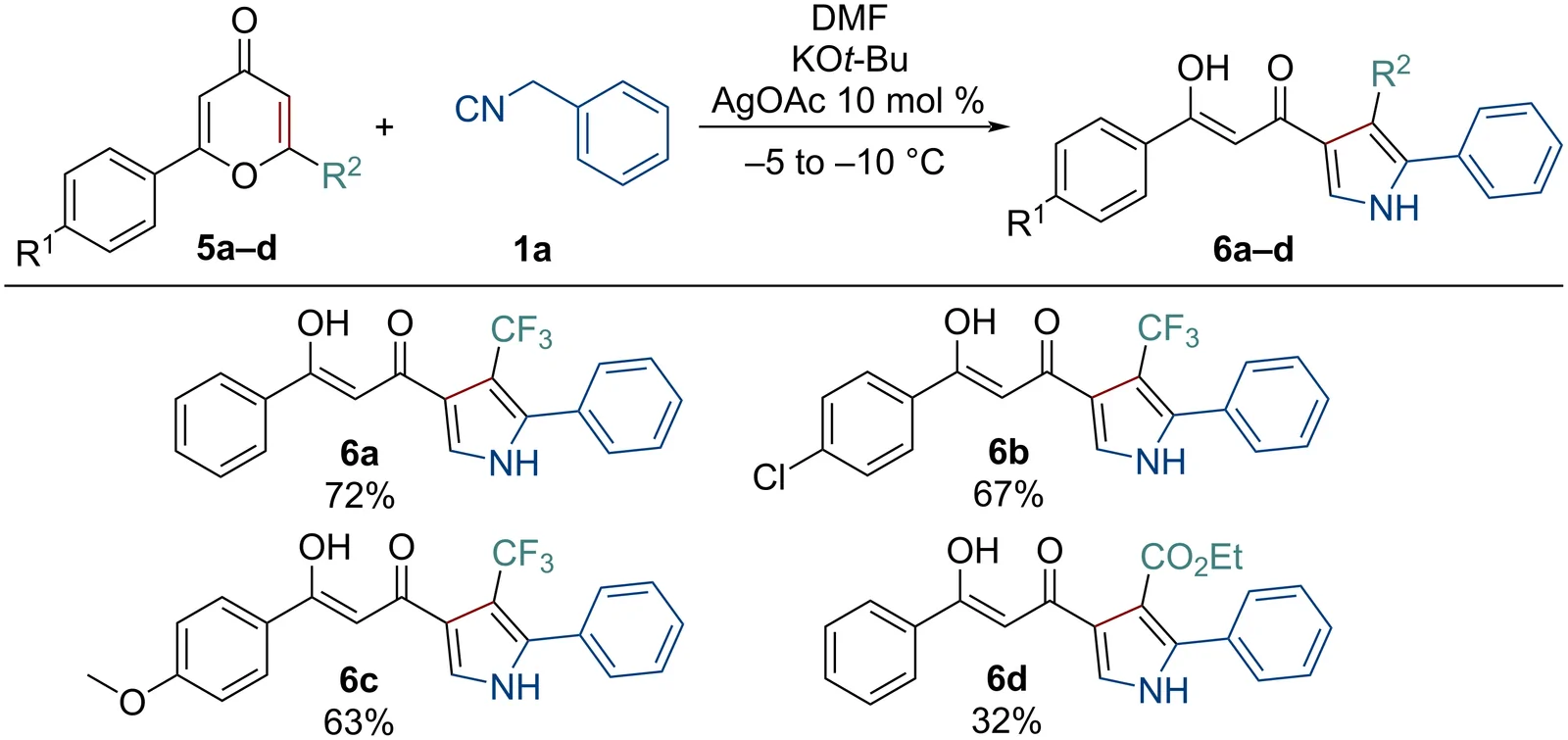
Cycloaddition of benzyl isocyanide (**1a**) with 4-pyrones **5a–d**. Reaction conditions: benzyl isocyanide (**1a**, 88 mg, 0.75 mmol, 1.5 equiv), 4-pyrones **5a–d** (0.5 mmol, 1.0 equiv), silver acetate (8 mg, 0.05 mmol, 0.1 equiv, 10 mol %), potassium *tert*-butoxide (112 mg, 1.0 mmol, 2.0 equiv), DMF (3 mL), **−**5 to **−**10 °C, 4 hours. All stated yields refer to isolated yields.

It should be noted that 6-(trifluoromethyl)-4*H*-pyrones may undergo different transformations, exhibiting reactivity similar to that of chromones [34,53,59]. In particular, they have been successfully used in [3 + 2] cycloaddition reactions, but pyrones remain less studied substrates due to their lower synthetic availability [34,59]. Previously, we reported a new method for the synthesis of 4-pyrones based on the dehydrative cyclization of 1,3,5-triketones [61]. Therefore, the use of pyrones in the [3 + 2] cycloaddition reaction of benzyl isocyanide increases the scope of the proposed methodology and provides structural diversity of the desired pyrroles (Scheme 3).

The use of 4-pyrones **5a–c** in the reaction with benzyl isocyanide (**1a**) under standard conditions produced 2-arylpyrroles **6a–c** with 63–72% yield. The presence of a methoxy group and a chlorine atom in the aromatic ring of the pyrone had only little effect on the yields of pyrroles **6b** and **6c**. Moreover, this methodology was successfully used in the synthesis of pyrrole **6d** from the ethyl ester of 6-phenylcomanic acid.

**Scheme 4:**
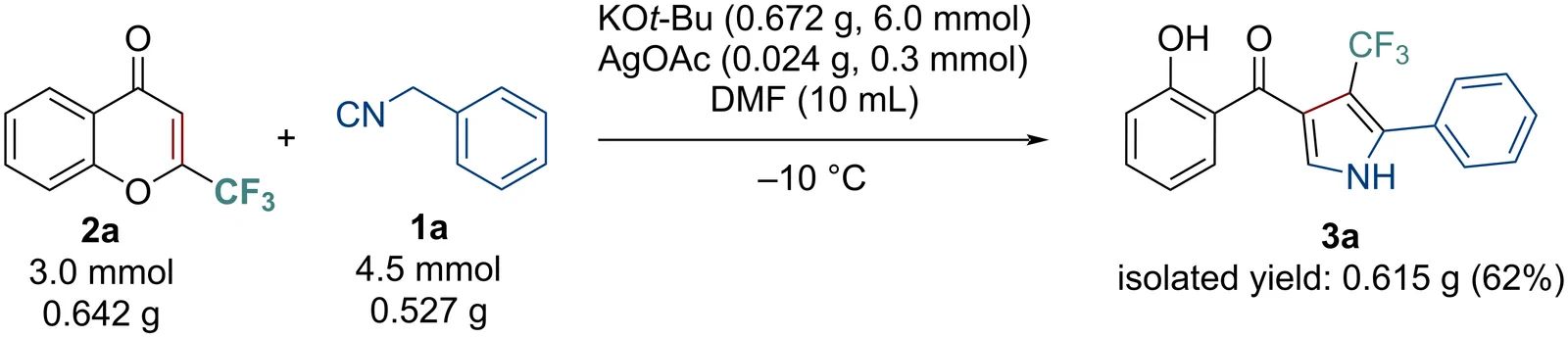
Large scale reaction of the [3 + 2] cycloaddition reaction. Reaction conditions: benzyl isocyanide (**1a**, 0.527 g, 4.5 mmol, 1.5 equiv), chromone **2a** (0.642 g, 3.0 mmol, 1.0 equiv), silver acetate (0.0240 mg, 0.15 mmol, 0.1 equiv, 10 mol %), base (1.0 mmol, 2.0 equiv), DMF (10 mL).

The structures of 2-arylpyrroles **3a–u** and **6a–d** were determined based on literature data and combined information from X-ray single-crystal analysis (CCDC 2297092, Figure 3), values of ^1^H,^1^H coupling constants as well as ^1^H, ^19^F and ^13^C chemical shifts (Supporting Information File 1) [33–34,48,62–63]. It is noteworthy that products **6a–d** were obtained as the enol tautomer with a *Z*-configuration of the double bond owing to the reaction mechanism and the stability of this tautomer due to an intramolecular hydrogen bond (Supporting Information File 1). Finally, to demonstrate the synthetic utility of this methodology, we scaled up the reaction to a 3.0 mmol scale, whereby 0.615 g of 2-arylpyrrole **3a** was obtained in 62% yield (Scheme 4. Supporting Information File 1).

**Scheme 5:**
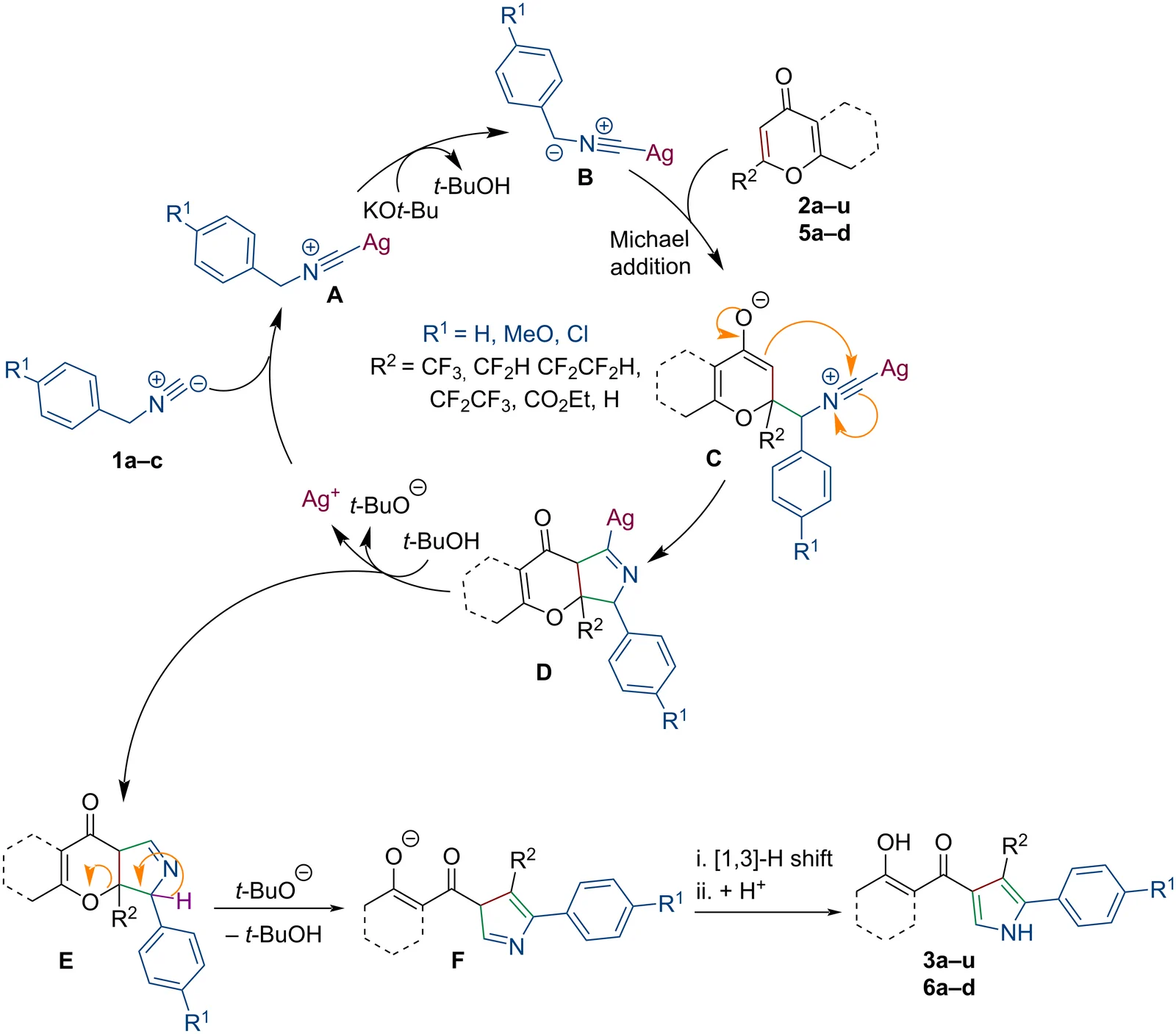
Proposed mechanism of [3 + 2] cycloaddition reaction.

The plausible reaction mechanism can be proposed based on literature data [33–34,38–50] (Scheme 5). The formation of 2-arylpyrroles **3a–u** and **6a–d** involves a catalytic cycle. In this cycle, silver acetate initially reacts with benzyl isocyanides, which leads to the formation of complex **A**. Complex **A** further interacts with potassium *tert*-butoxide and gives anion **B**. It is likely that the use of a catalyst is necessary both to increase the acidity of the benzyl proton and to stabilize anion **B**. Complex **B** subsequently undergoes Michael addition to chromones **2a–u** or pyrones **5a–d**, forming intermediate **C**. Complex **C** is transformed to **D** through intramolecular cyclization. After cleavage of the metal ion from **D**, the catalytic cycle is completed, and pyrroline **E** is formed. Then the chromane/pyran ring opens after deprotonation of pyrroline **E** to give intermediate **F**. Finally, intermediate **F** after a 1,3-hydrogen shift and subsequent acidification, affords 2-arylpyrroles **3a–u** and **6a–d**.

## Conclusion

We have developed a one-pot, silver-catalyzed base-induced anionic [3 + 2] cycloaddition of benzyl isocyanides with 2-polyfluoroalkylchromones and 6-(trifluoromethyl)-4*H*-pyrones for the direct synthesis of 2-arylpyrroles. The reaction proceeds under mild conditions (10 mol % of AgOAc, 2.0 equiv of KO*t*-Bu, DMF) and furnishes 2-arylpyrroles **3a–t** in yields of up to 83% with high chemo- and regioselectivity. The scope was extended to 6-(trifluoromethyl)-4*H*-pyrones, affording the corresponding pyrroles **6a–c** in up to 72% yield, and further to chromones and 4-pyrones lacking polyfluoroalkyl groups, yielding pyrroles **3s**, **3t** and **6d**. This operationally simple method provides a versatile and practical entry to the 2-arylpyrrole scaffold, a privileged motif in medicinal chemistry and materials science.

## Supporting Information

CCDC 2297092 contains the supplementary crystallographic data for this paper. These data can be obtained free of charge via https://www.ccdc.cam.ac.uk/data_request/cif, or by emailing data_request@ccdc.cam.ac.uk, or by contacting The Cambridge Crystallographic Data Centre, 12 Union Road, Cambridge CB2 1EZ, UK; fax: +44 1223336033.

File 1 Materials and methods, detailed optimization table, experimental procedure, characterization data, сopies of ^1^H NMR, ^19^F NMR and ^13^C NMR spectra for all new compounds.

## Data Availability

All data that supports the findings of this study is available in the published article and/or the supporting information of this article.
