# Supplementary material for: Silver-catalyzed anionic [3 + 2] cycloaddition of benzyl isocyanides with polyfluoroalkylchromones and pyrones: direct synthesis of 2-arylpyrroles
Source: Beilstein J Org Chem. 2026 Sep 23;22:1284–94. doi: 10.3762/bjoc.22.103 (PMC13618341; doi:10.3762/bjoc.22.103)
Supplement: Supplementary file 1 [file Beilstein_J_Org_Chem-22-1284-s001.pdf]

BEILSTEIN JOURNAL OF ORGANIC CHEMISTRY

Supporting Information

for

Silver-catalyzed anionic [3 + 2] cycloaddition of benzyl isocyanides with polyfluoroalkylchromones and pyrones: direct synthesis of 2-arylpyrroles

Ivan A. Kochnev and Alexey Yu. Barkov

Beilstein J. Org. Chem. **2026,** *22,* 1284–1294. doi:10.3762/bjoc.22.103

Materials and methods, detailed optimization table, experimental procedure, characterization data, copies of ^{1}H NMR, ^{19}F NMR and ^{13}C NMR spectra for all new compounds

License and Terms: This is a supporting information file under the terms of the Creative Commons Attribution License (https://creativecommons.org/licenses/by/4.0). Please note that the reuse, redistribution and reproduction in particular requires that the author(s) and source are credited and that individual graphics may be subject to special legal provisions.
The license is subject to the *Beilstein Journal of Organic Chemistry* terms and conditions: (https://www.beilstein-journals.org/bjoc/terms)

Contents

1. Experimental section S2

   i. General procedure for the synthesis of 2-arylpyrroles **3a–t** and **6a–d** S2

   ii. Illustrated synthesis of the 2-arylpyrrole **2a**...S2–S3

   iii. Analytical data for 2-arylpyrroles **3a-t** S4–S10

   iv. Analytical data for the mixture of 2-arylpyrroles **3u**, **4u** S10

   v. Analytical data for 2-arylpyrroles **6a–d**...S11–S12

2. Copy of ^{1}H and ^{13}C NMR spectrum of pyrroles **3a–t**...S13–S50

3. Copy of ^{1}H and ^{13}C NMR ^{19}F HRMS spectrum of pyrroles **3u** and **4u** S51–S53

4. Copy of ^{1}H and ^{13}C NMR spectrum of pyrroles **6a–d**..S54–S60

5. Table S1 Crystal data and structure refinement for **3f**...S61

6. Citing literature...S62

1. Experimental section

General information

NMR spectra were recorded on Bruker Avance III-500 (^{1}H, 500 MHz; ^{19}F, 471 MHz; ^{13}C, 126 MHz) and Bruker DRX-400 (^{1}H, 400 MHz; ^{19}F, 376 MHz) spectrometers in $CDCl_3$. The chemical shifts (δ) are reported in ppm relative to the internal standard TMS (^{1}H NMR), C_6F_6 (^{19}F NMR), and the residual signal of the solvent (^{13}C NMR). The HRMS spectra were obtained using the UHR-QqTOF maXis Impact HD (Bruker Daltonics, Billerica, MA, USA) mass spectrometer. Melting points were determined on a Stuart SMP40 apparatus. Monitoring of the reaction progress and assessment of the purity of the synthesized compounds were carried out by TLC on Sorbfil PTSKh-AF-A-UF plates (eluent EtOAc–hexane 1:2, eluent EtOAc–hexane 1:3). Unless otherwise noted, all solvents and other reagents were commercially available and were used without further purification. All reagents were weighed and handled in air at room temperature. All solvents used were dried and distilled by standard procedures. The starting benzyl isocyanides **1a–d**, chromones **2a–l**, and 4-pyrones **5a–d** were prepared according to the described procedures [1-4].

i. General procedure for the synthesis of 2-arylpyrroles 3a–t and 6a–d

A 10 mL reaction tube equipped with a magnetic stirrer under an argon atmosphere was charged with chromones **2a–l** or pyrones **5a–d** (0.5 mmol, 1.0 equiv), silver acetate (8.3 mg, 0.05 mmol, 10 mol %, 0.1 equiv) and 3 mL dry DMF. The mixture was stirred at room temperature for 2 min, and then benzyl isocyanides **1a–c** (0.75 mmol, 1.5 equiv), was added, and the reaction mixture was stirred at a temperature from –5 to –10 °C until complete dissolution of the reagents. Then, potassium *tert*-butoxide (112 mg, 1.0 mmol, 2.0 equiv) was added to the resulting solution in one portion. The reaction mixture was stirred for 4 hours at –5 to –10 °C and was subsequently quenched with diluted hydrochloric acid (20 mL). The product was extracted with EtOAc (4 ×10 mL), washed with brine (2 × 10 mL), dried over anhydrous $MgSO_4$, and evaporated to dryness to yield a crude reaction product, which was purified by silica gel column chromatography eluting with EtOAc-Hexane (1:2) to give the desired 2-arylpyrroles **3a–t** and **6a–d**.

ii. Illustrated synthesis of the 2-arylpyrrole 2a

A 50 mL reaction tube equipped with a magnetic stirrer under an argon atmosphere was charged with chromones **2a** (0. 642 g, 3.0 mmol, 1.0 equiv), silver acetate (0.025 g, 0.15 mmol, 10 mol %, 0.1 equiv) and 10 mL dry DMF. The mixture was stirred at room temperature for 2 min, and then benzyl isocyanide **1a** (0.527 g, 4.5 mmol, 1.5 equiv), was added, and the reaction

mixture was stirred at a temperature from –5 to –10 °C until complete dissolution of the reagents. Then potassium *tert*-butoxide (0.672 g, 4.0 mmol, 2.0 equiv) was added to the resulting solution in one portion. The reaction mixture was stirred for 4 hours at –5 to –10 °C and was quenched with diluted hydrochloric acid (220 mL). The product was extracted with EtOAc (4 × 40 mL), washed with brine (2 × 50 mL), dried over anhydrous $MgSO_4$, and evaporated to dryness to yield a crude reaction product, which was purified by silica gel column chromatography eluting with EtOAc-Hexane (1:2) to give 0.615 g of the 2-phenylpyrrole **3a** with 62% yield.

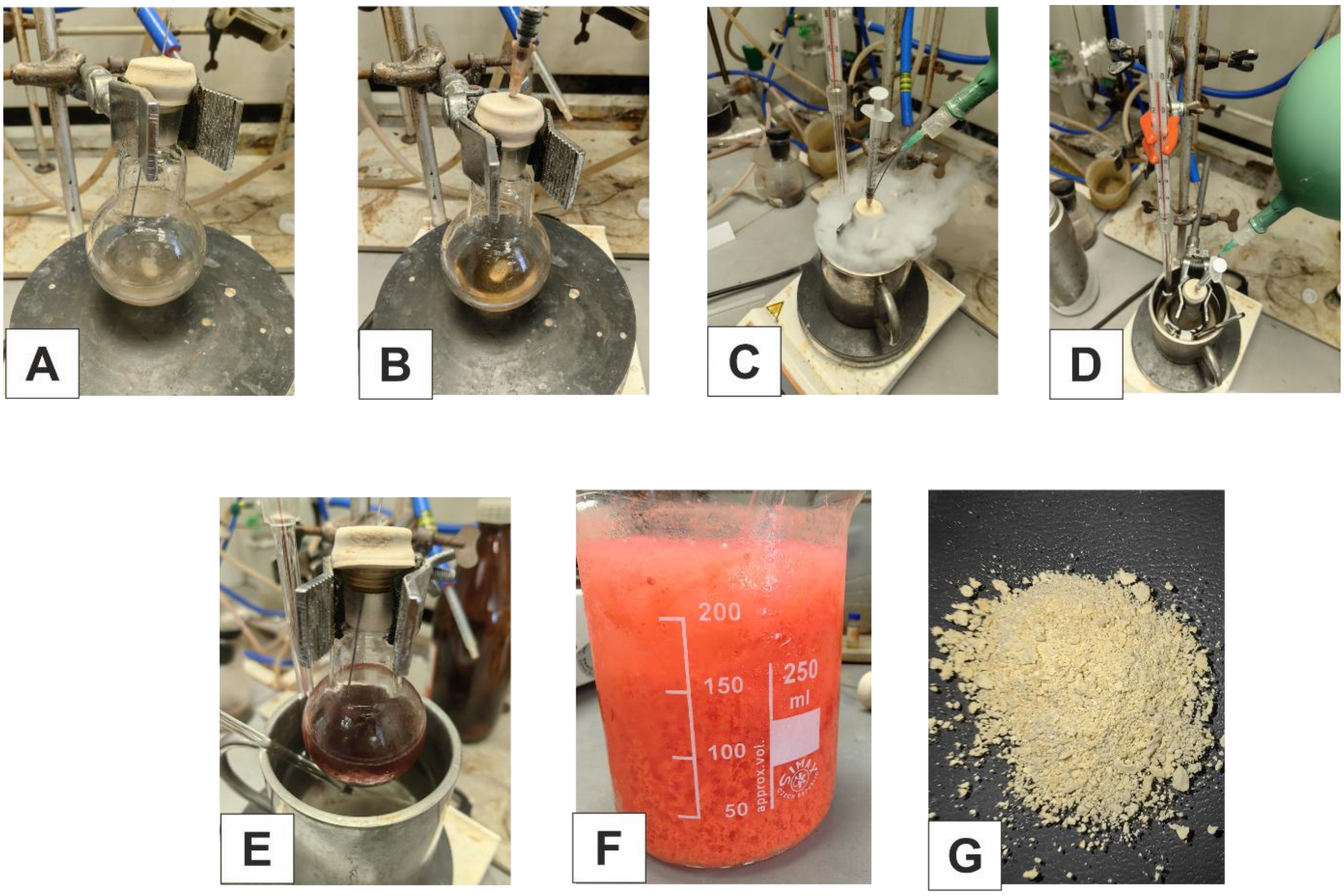

A: The suspension of chromone **2a** and silver acetate in dry DMF.

B: The resulting solution after benzyl isocyanide addition.

C: Cooling of the reaction mixture to –5 to –10 °C in *n*-butanol-liquid N_2 bath.

D: The cooled reaction mixture after potassium *tert*-butoxide addition.

E: The reaction mixture after 4 hours of stirring.

F: The crude reaction product after quenching with hydrochloric acid.

G: The pure 2-phenylpyrrole **3a**.

iii. Analytical data for 2-arylpyrroles 3a–t

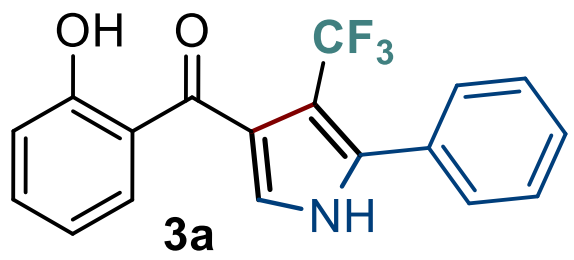

3a

(2-Hydroxyphenyl)(5-phenyl-4-(trifluoromethyl)-1*H*-pyrrol-3-yl)methanone (3a): beige solid (137 mg, 83%), mp 133–135 °C [4]; **^{1}H NMR** (400 MHz, $CDCl_3$) δ 12.03 (s, 1H), 8.74 (s, 1H), 7.83 (dd, J = 8.0, 1.7 Hz, 1H), 7.56 – 7.43 (m, 6H), 7.16 (s, 1H), 7.04 (dd, J = 8.4, 1.1 Hz, 1H), 6.95 – 6.87 (m, 1H).

^{19}F NMR (376 MHz, $CDCl_3$) δ 109.12.

^{13}C NMR (126 MHz, $CDCl_3$) δ 194.8, 162.9, 136.3, 135.6 (q, J = 4.0 Hz), 132.9, 130.4, 129.5, 128.9 (4C), 124.0, 123.3 (q, J = 268.2 Hz), 122.4 (q, J = 1.9 Hz), 120.6, 118.9, 118.4, 110.7 (q, J = 36.6 Hz).

HRMS (ESI): m/z calcd. for $C_{18}H_{13}F_3NO_2$ $[M + H]^+$, 332.0893; found, 332.0891.

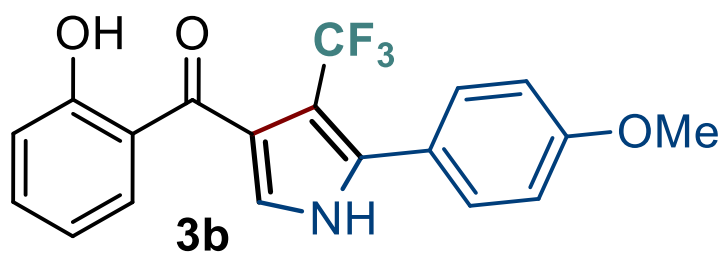

3b

(2-Hydroxyphenyl)(5-(4-methoxyphenyl)-4-(trifluoromethyl)-1*H*-pyrrol-3-yl)methanone (3b): beige solid (70 mg, 39%), mp 148–150 °C;

^{1}H NMR (400 MHz, $CDCl_3$) δ 12.04 (s, 1H), 8.65 (s, 1H), 7.83 (dd, J = 7.9, 1.7 Hz, 1H), 7.51 (ddd, J = 8.6, 7.2, 1.7 Hz, 1H), 7.47 – 7.40 (m, 2H), 7.14 (d, J = 2.8 Hz, 1H), 7.05 (dd, J = 8.4, 1.1 Hz, 1H), 7.02 – 6.95 (m, 2H), 6.91 (ddd, J = 8.2, 7.2, 1.2 Hz, 1H), 3.86 (s, 3H).

^{19}F NMR (376 MHz, $CDCl_3$) δ 109.03.

^{13}C NMR (126 MHz, $CDCl_3$) δ 194.8, 162.9, 160.5, 136.2, 135.6 (q, J = 4.1 Hz), 132.9, 130.2 (q, J = 1.7 Hz) (2C), 123.8, 123.4 (q, J = 268.2 Hz), 122.8, 122.6 (d, J = 2.4 Hz), 120.6, 118.8, 118.3, 114.3 (2C), 110.1 (q, J = 36.4 Hz), 55.5.

HRMS (ESI): m/z calcd for $C_{19}H_{15}F_3NO_3$ $[M + H]^+$, 362.0999; found, 362.1000.

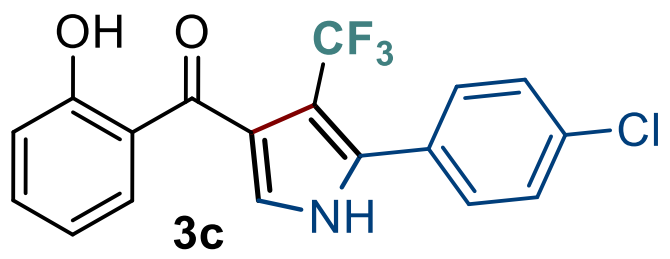

3c

(5-(4-Chlorophenyl)-4-(trifluoromethyl)-1*H*-pyrrol-3-yl)(2-hydroxyphenyl)methanone (3c): beige solid (89 mg, 49%), mp 176–178 °C;

^{1}H NMR (400 MHz, $CDCl_3$) δ 11.91 (s, 1H), 8.71 (s, 1H), 7.81 (d, J = 2.6 Hz, 1H), 7.56 – 7.42 (m, 6H), 7.22 (d, J = 2.9 Hz, 1H), 7.02 (d, J = 8.8 Hz, 1H).

^{19}F NMR (376 MHz, $CDCl_3$) δ 109.03.

^{13}C NMR (126 MHz, $CDCl_3$) δ 193.5, 161.5, 136.1, 135.9 (q, J = 4.0 Hz), 131.8, 130.3, 129.6, 129.0 (4C), 124.2, 123.6, 123.1 (q, J = 266.6 Hz), 122.0 (q, J = 2.0 Hz), 121.2, 120.1, 110.8 (q, J = 36.6 Hz).

HRMS (ESI): m/z calcd for $C_{18}H_{12}ClF_3NO_2$ $[M + H]^+$, 366.0503; found, 366.0504.

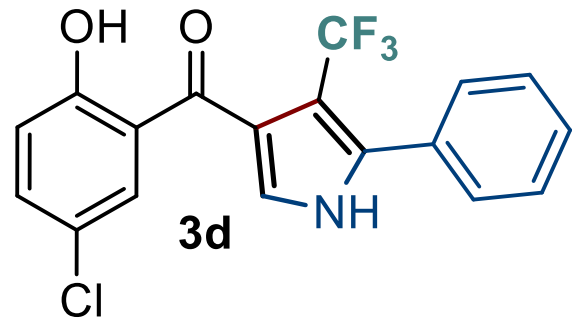

(5-Chloro-2-hydroxyphenyl)(5-phenyl-4-(trifluoromethyl)-1*H*-pyrrol-3-yl)methanone (3d): beige solid (110 mg, 60%), mp 195–197 °C;

^{1}H NMR (400 MHz, $CDCl_3$) δ 11.88 (s, 1H), 8.70 (s, 1H), 7.78 (d, *J* = 2.7 Hz, 1H), 7.50 – 7.42 (m, 4H), 7.23 (d, *J* = 2.8 Hz, 1H), 7.02 (d, *J* = 8.9 Hz, 1H).

^{19}F NMR (376 MHz, $CDCl_3$) δ 108.97.

^{13}C NMR (126 MHz, DMSO-*d6*) δ 188.7, 156.0, 134.3 (q, *J* = 4.2 Hz), 133.7, 132.5, 131.0 (2C), 129.3 (2C), 129.1, 128.3 (2C), 126.9, 123.4 (q, *J* = 268.0 Hz), 122.3, 122.1, 118.7, 108.0 (q, *J* = 35.8 Hz).

HRMS (ESI): m/z calcd for $C_{18}H_{12}ClF_3NO_2$ $[M + H]^+$, 366.0503; found, 366.0500.

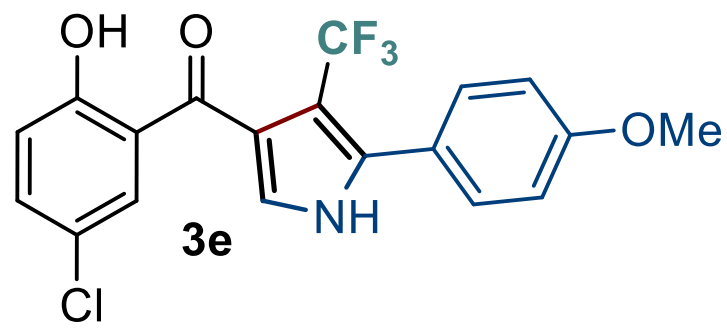

(5-Chloro-2-hydroxyphenyl)(5-(4-methoxyphenyl)-4-(trifluoromethyl)-1*H*-pyrrol-3-yl)methanone (3e): beige solid (32 mg, 16%), mp 177–179 °C;

^{1}H NMR (400 MHz, $CDCl_3$) δ 11.91 (s, 1H), 8.60 (s, 1H), 7.80 (d, *J* = 2.7 Hz, 1H), 7.49 – 7.41 (m, 3H), 7.19 (d, *J* = 2.9 Hz, 1H), 7.05 – 6.97 (m, 3H), 3.87 (s, 3H).

^{19}F NMR (376 MHz, $CDCl_3$) δ 108.91.

^{13}C NMR (126 MHz, DMSO-*d6*) δ 188.9, 159.7, 156.2, 135.8 (q, *J* = 4.1 Hz), 132.5, 130.5 (2C), 129.5, 128.6, 126.8, 123.6 (q, *J* = 267.6 Hz), 122.8, 122.3, 122.0, 118.7, 113.8 (2C), 107.2 (q, *J* = 35.6 Hz), 55.2.

HRMS (ESI): m/z calcd for $C_{19}H_{14}ClF_3NO_3$ $[M + H]^+$, 396.0609; found, 396.0607.

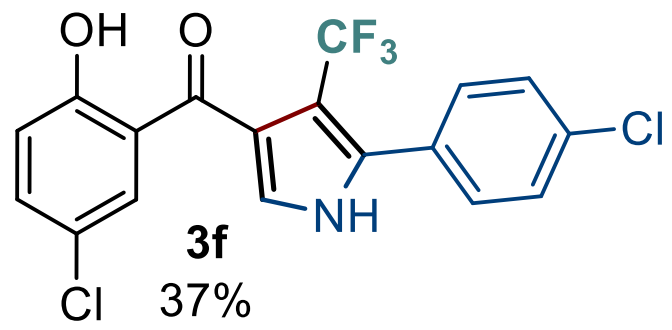

(5-Chloro-2-hydroxyphenyl)(5-(4-chlorophenyl)-4-(trifluoromethyl)-1*H*-pyrrol-3-yl)methanone (3f): beige solid (74 mg, 37%), mp 229–231 °C;

^{1}H NMR (400 MHz, $CDCl_3$) δ 11.88 (s, 1H), 8.70 (s, 1H), 7.78 (d, *J* = 2.7 Hz, 1H), 7.50 – 7.42 (m, 4H), 7.23 (d, *J* = 2.8 Hz, 1H), 7.02 (d, *J* = 8.9 Hz, 1H).

^{19}F NMR (376 MHz, $CDCl_3$) δ 108.93.

^{13}C NMR (126 MHz, DMSO-*d6*) δ 188.7, 156.0, 134.3 (q, *J* = 4.2 Hz), 133.7, 132.5, 131.0 (2C), 129.3 (2C), 129.1, 128.3 (2C), 126.9, 123.4 (q, *J* = 268.0 Hz), 122.3, 122.1, 118.7, 108.0 (q, *J* = 35.8 Hz).

HRMS (ESI): m/z calcd for $C_{18}H_{10}Cl_2F_3NNaO_2$ $[M + Na]^+$, 421.9933; found, 421.9930.

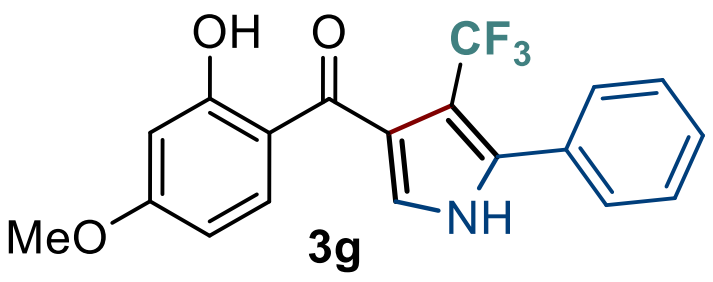

3g

(2-Hydroxy-4-methoxyphenyl)(5-phenyl-4-(trifluoromethyl)-1*H*-pyrrol-3-yl)methanone (3g): beige solid (146 mg, 81%), mp 138–140 °C;

^{1}H NMR (400 MHz, $CDCl_3$) δ 12.66 (s, 1H), 8.80 (s, 1H), 7.73 (d, J = 8.8 Hz, 1H), 7.54 – 7.42 (m, 5H), 7.09 (s, 1H), 6.49 (d, J = 2.4 Hz, 1H), 6.44 (dd, J = 9.0, 2.4 Hz, 1H), 3.86 (s, 3H).

^{19}F NMR (376 MHz, $CDCl_3$) δ 109.29.

^{13}C NMR (126 MHz, $CDCl_3$) δ 193.5, 166.3, 166.0, 135.2 (q, J = 4.2 Hz), 134.7, 130.6, 129.3, 128.9 (3C), 127.0, 123.4 (q, J = 268.4 Hz), 122.5 (q, J = 2.1 Hz), 122.9, 114.6, 110.5 (q, J = 36.6 Hz), 107.5, 101.1, 55.8.

HRMS (ESI): m/z calcd for $C_{19}H_{15}F_3NO_3$ $[M + H]^+$, 362.0999; found, 362.1000.

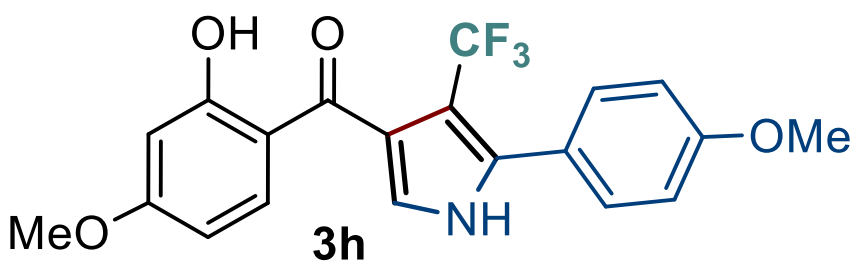

3h

(2-Hydroxy-4-methoxyphenyl)(5-(4-methoxyphenyl)-4-(trifluoromethyl)-1*H*-pyrrol-3-yl)methanone (3h): beige solid (61 mg, 31%), mp 153–155 °C;

^{1}H NMR (400 MHz, $CDCl_3$) δ 12.67 (s, 1H), 8.59 (s, 1H), 7.73 (d, J = 8.9 Hz, 1H), 7.43 (d, J = 8.4 Hz, 2H), 7.07 (d, J = 2.6 Hz, 1H), 7.01 – 6.95 (m, 2H), 6.50 (d, J = 2.4 Hz, 1H), 6.44 (dd, J = 8.9, 2.5 Hz, 1H), 3.86 (s, 6H).

^{19}F NMR (376 MHz, $CDCl_3$) δ 109.17.

^{13}C NMR (126 MHz, $CDCl_3$) δ 193.6, 166.3, 166.0, 160.5, 135.2 (q, J = 4.1 Hz), 134.6, 130.1 (2C), 123.5 (q, J = 268.0 Hz), 122.9, 122.6, 122.5 (q, J = 1.7 Hz), 114.6, 114.3 (2C), 110.0 (q, J = 36.2 Hz), 107.4, 101.1, 55.8, 55.5.

HRMS (ESI): m/z calcd for $C_{20}H_{17}F_3NO_4$ $[M + H]^+$, 392.1104; found, 392.1108

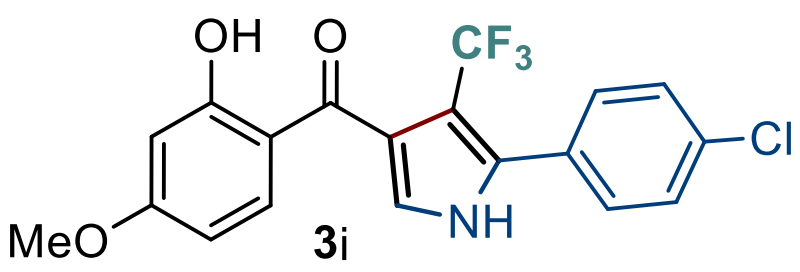

3i

(5-(4-Chlorophenyl)-4-(trifluoromethyl)-1*H*-pyrrol-3-yl)(2-hydroxy-4-methoxyphenyl)methanone (3i): beige solid (105 mg, 53%), mp 192–194 °C;

^{1}H NMR (400 MHz, $CDCl_3$) δ 12.62 (s, 1H), 8.69 (s, 1H), 7.70 (d, J = 8.9 Hz, 1H), 7.11 (d, J = 2.7 Hz, 1H), 6.50 (d, J = 2.4 Hz, 1H), 6.44 (dd, J = 8.9, 2.5 Hz, 1H), 3.87 (s, 3H).

^{19}F NMR (376 MHz, $CDCl_3$) δ 109.18.

^{13}C NMR (126 MHz, DMSO-*d6*) δ 192.6, 165.5, 164.4, 134.6, 133.7 (2C), 133.6 (q, J = 4.0 Hz), 130.7, 129.3, 128.5 (2C), 125.7, 123.6 (q, J = 267.6 Hz), 121.00 (q, J = 1.5 Hz), 114.3, 108.5 (q, J = 35.3 Hz), 107.0, 101.1, 55.7.

HRMS (ESI): m/z calcd for $C_{19}H_{14}ClF_3NO_3$ $[M + H]^+$, 396.0609; found, 396.0608.

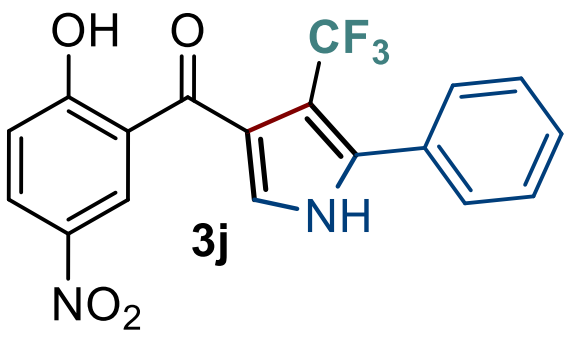

3j

(2-Hydroxy-5-nitrophenyl)(5-phenyl-4-(trifluoromethyl)-1*H*-pyrrol-3-yl)methanone (3j): white solid (103 mg, 55%), mp 207–209 °C;

^{1}H NMR (400 MHz, $CDCl_3$) δ 12.75 (s, 1H), 8.84 (d, *J* = 2.8 Hz, 1H), 8.78 (s, 1H), 8.40 (dd, *J* = 9.2, 2.8 Hz, 1H), 7.57 – 7.47 (m, 5H), 7.30 (d, *J* = 2.8 Hz, 1H), 7.17 (d, *J* = 9.2 Hz, 1H).

^{19}F NMR (376 MHz, $CDCl_3$) δ 108.89.

^{13}C NMR (126 MHz, DMSO-*d6*) δ 187.0, 162.2, 139.1, 136.1 (q, *J* = 4.1 Hz),130.5, 129.9, 129.3 (2C), 128.9, 128.2 (2C), 127.7, 126.8, 125.8, 123.4 (q, *J* = 268.4 Hz), 122.2, 117.3, 107.7 (q, *J* = 36.0 Hz).

HRMS (ESI): m/z calcd for $C_{18}H_{12}F_3N_2O_4$ $[M + H]^+$, 377.0744; found, 377.0739.

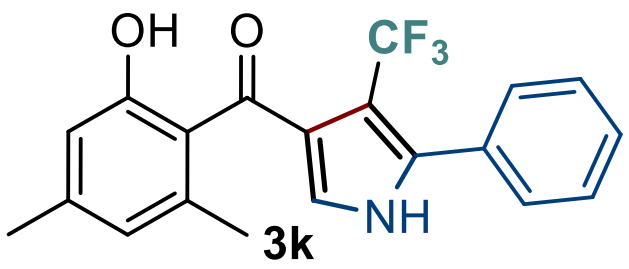

3k

(2-Hydroxy-4,6-dimethylphenyl)(5-phenyl-4-(trifluoromethyl)-1*H*-pyrrol-3-yl)methanone (3k): beige solid (72 mg, 40%), mp 157–159 °C;

^{1}H NMR (400 MHz, $CDCl_3$) δ 9.62 (s, 1H), 8.54 (s, 1H), 7.57 – 7.43 (m, 5H), 6.95 (d, *J* = 2.9 Hz, 1H), 6.66 (s, 1H), 6.57 (s, 1H), 2.31 (s, 3H), 2.17 (s, 3H).

^{19}F NMR (376 MHz, $CDCl_3$) δ 108.62.

^{13}C NMR (126 MHz, $CDCl_3$) δ 194.2, 159.7, 144.7, 139.0, 135.7 (q, *J* = 4.7 Hz),130.5, 129.5, 129.0 (2C), 128.9 (2C), 126.7 (q, *J* = 1.7 Hz), 123.3 (q, *J* = 268.0 Hz), 124.5, 124.2, 121.7, 115.5, 109.7 (q, *J* = 36.5 Hz), 22.7, 21.8.

HRMS (ESI): m/z calcd for $C_{20}H_{17}F_3NO_2$ $[M + H]^+$, 360.1206; found, 360.1209.

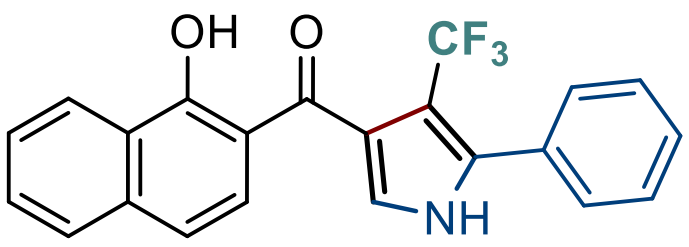

3l

(1-Hydroxynaphthalen-2-yl)(5-phenyl-4-(trifluoromethyl)-1*H*-pyrrol-3-yl)methanone (3l): light yellow solid (95 mg, 50%), mp 188–190 °C;

^{1}H NMR (400 MHz, $CDCl_3$) δ 13.91 (s, 1H), 8.66 (s, 1H), 8.52 (d, *J* = 8.3 Hz, 1H), 7.78 (dd, *J* = 8.6, 2.8 Hz, 2H), 7.65 (ddd, *J* = 8.1, 6.8, 1.3 Hz, 1H), 7.60 – 7.44 (m, 6H), 7.28 (d, *J* = 8.4 Hz, 1H), 7.22 – 7.17 (m, 1H).

^{19}F NMR (376 MHz, $CDCl_3$) δ 109.26.

^{13}C NMR (126 MHz, DMSO-*d6*) δ 194.2, 161.6, 136.7, 135.3 (q, *J* = 4.2 Hz), 130.4, 130.2, 129.0 (2C), 128.9, 128.5 (2C), 127.6, 127.0, 126.2, 126.2, 124.3, 123.7 (q, *J* = 267.4 Hz), 123.5, 120.7, 118.2, 113.7, 108.3 (q, *J* = 35.6 Hz).

HRMS (ESI): m/z calcd for $C_{22}H_{15}F3NO_2$ $[M + H]^+$, 382,1049; found, 382.1048.

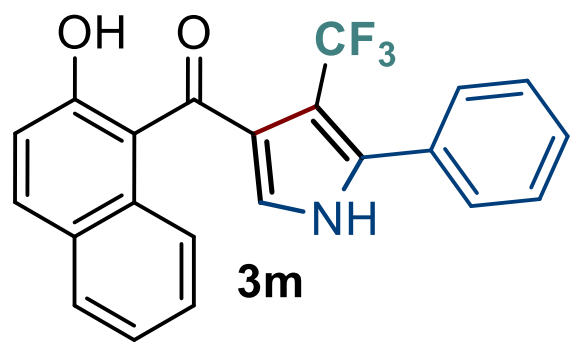

(2-Hydroxynaphthalen-1-yl)(5-phenyl-4-(trifluoromethyl)-1*H*-pyrrol-3-yl)methanone (3m): beige solid (101 mg, 53%), mp 133–135 °C;

^{1}H NMR (400 MHz, $CDCl_3$) δ 11.07 (s, 1H), 8.48 (s, 1H), 7.90 (d, *J* = 9.0 Hz, 1H), 7.84 – 7.71 (m, 2H), 7.50 – 7.45 (m, 5H), 7.33 – 7.30 (m, 2H), 7.19 (d, *J* = 9.0 Hz, 1H), 6.72 (s, 1H).

^{19}F NMR (376 MHz, $CDCl_3$) δ 108.79.

^{13}C NMR (126 MHz, DMSO-*d6*) δ 189.4, 151.5, 135.9 (q, *J* = 4.3 Hz), 131.7, 130.8, 130.2, 129.9, 129.4 (2C), 128.8, 128.2 (2C), 128.1, 127.5, 126.9, 124.4, 123.7 (q, *J* = 267.6 Hz), 123.2, 122.9, 121.5, 118.3, 107.1 (q, *J* = 36.2 Hz).

HRMS (ESI): m/z calcd for $C_{22}H_{15}F_3NO_2$ $[M + H]^+$, 382,1049; found, 382.1050.

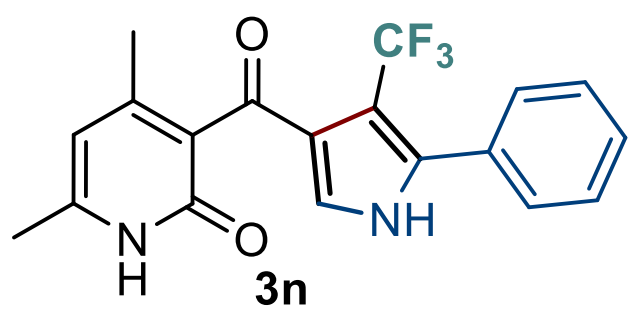

4,6-Dimethyl-3-(5-phenyl-4-(trifluoromethyl)-1*H*-pyrrole-3-carbonyl)pyridin-2(1*H*)-one (3n): white solid (108 mg, 60%), mp 285–287 °C (decomposition);

^{1}H NMR (400 MHz, DMSO-*d6*) δ 12.22 (s, 1H), 11.68 (s, 1H), 7.51 – 7.41 (m, 5H), 7.31 (d, *J* = 3.1 Hz, 1H), 5.96 (s, 1H), 2.17 (s, 3H), 1.98 (s, 3H).

^{19}F NMR (376 MHz, DMSO-*d6*) δ 110.75.

^{13}C NMR (126 MHz, DMSO-*d6*) δ 188.3, 161.0, 148.4, 145.0, 135.7 (q, *J* = 4.5 Hz), 130.9, 129.4 (2C), 129.3, 128.8, 128.2 (2C), 127.5, 123.7, 123.6 (q, *J* = 267.6 Hz), 107.0 (q, *J* = 35.6 Hz),106.9, 18.8, 18.4.

HRMS (ESI): m/z calcd for $C_{19}H_{16}F_3N_2O_2$ $[M + H]^+$, 361.1158; found, 361.1157.

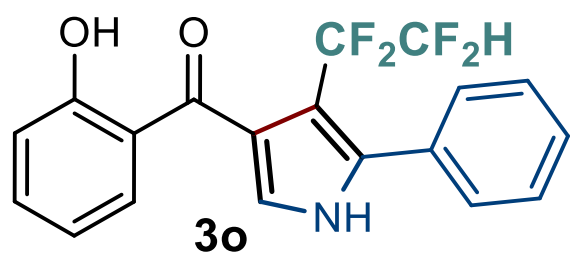

(2-Hydroxyphenyl)(5-phenyl-4-(1,1,2,2-tetrafluoroethyl)-1*H*-pyrrol-3-yl)methanone (3o): beige solid (109 mg, 60%), mp 145–147 °C;

^{1}H NMR (400 MHz, $CDCl_3$) δ 11.87 (s, 1H), 8.62 (s, 1H), 7.85 (dd, *J* = 8.0, 1.7 Hz, 1H), 7.56 – 7.49 (m, 3H), 7.48 – 7.42 (m, 3H), 7.21 (d, *J* = 3.0 Hz, 1H), 7.05 (dd, *J* = 8.5, 1.1 Hz, 1H), 6.94 – 6.90 (m, 1H), 6.89 (tt, *J* = 54.2, 6.0 Hz, 1H).

^{19}F NMR (376 MHz, $CDCl_3$) δ 53.49 (q, *J* = 9.4 Hz), 25.22 (dt, *J* = 54.2, 10.4 Hz),

^{13}C NMR (126 MHz, $CDCl_3$) δ 194.9, 162.9, 136.4 (t, *J* = 4.6 Hz), 136.2, 132.9, 131.1, 129.4 (2C), 129.3, 128.6 (2C), 125.5, 122.3 (t, *J* = 3.7 Hz), 120.6, 118.9, 118.4, 115.4 (tt, *J* = 249.6, 26.5 Hz), 111.3 (t, *J* = 26.5 Hz), 111.2 (tt, *J* = 250.8, 34.9 Hz).

HRMS (ESI): m/z calcd for $C_{19}H_{14}F_4NO_2$ [M + H]$^+$, 364.0955; found; 364.0948.

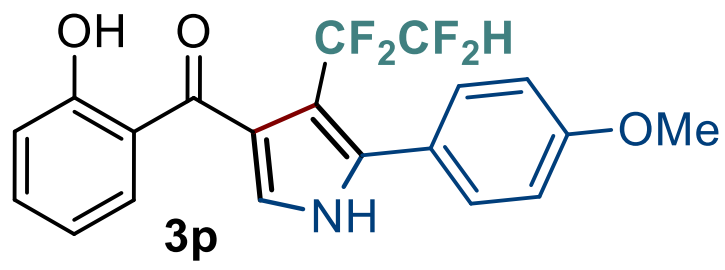

(2-Hydroxyphenyl)(5-(4-methoxyphenyl)-4-(1,1,2,2-tetrafluoroethyl)-1*H*-pyrrol-3-yl)methanone (3p): beige solid (20 mg, 10%), mp 130–132 °C;

^{1}H NMR (400 MHz, $CDCl_3$) δ 11.87 (s, 1H), 8.55 (s, 1H), 7.84 (dd, *J* = 7.9, 1.7 Hz, 1H), 7.51 (t, *J* = 7.7 Hz, 1H), 7.44 (d, *J* = 8.3 Hz, 2H), 7.18 (d, *J* = 2.7 Hz, 1H), 7.04 (d, *J* = 8.4 Hz, 1H), 6.97 (d, *J* = 8.3 Hz, 2H), 6.91 (t, *J* = 7.7 Hz, 1H), 6.88 (tt, *J* = 54.5, 6.2 Hz, 1H), 3.86 (s, 3H).

^{19}F NMR (376 MHz, $CDCl_3$) δ 53.27 (q, *J* = 10.5 Hz), 25.18 (dt, *J* = 54.1, 10.5 Hz).

^{13}C NMR (126 MHz, DMSO-*d6*) δ 192.1, 159.5, 158.2, 136.1 (t, *J* = 4.5 Hz), 133.6, 131.0, 130.6 (2C), 129.2, 124.3, 123.5, 121.8 (t, *J* = 2.6 Hz), 118.9, 117.0, 115.7 (tt, *J* = 247.8, 27.1 Hz), 110.7 (tt, *J* = 248.5, 34.2 Hz), 108.1 (t, *J* = 26.0 Hz), 113.5 (2C), 55.2.

HRMS (ESI): m/z calcd for $C_{20}H_{15}F_4NNaO_3$ [M + H]$^+$, 394.1061; found, 394.1056.

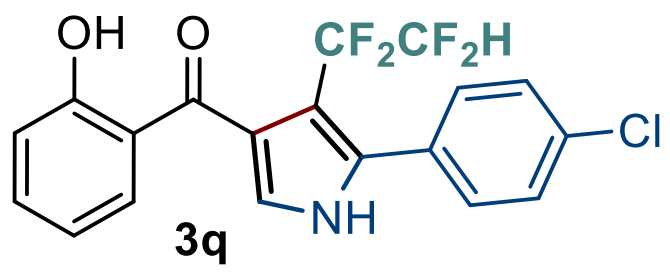

(5-(4-Chlorophenyl)-4-(1,1,2,2-tetrafluoroethyl)-1*H*-pyrrol-3-yl)(2-hydroxyphenyl)methanone (3q): beige solid (90 mg, 45%), mp 146–148 °C;

^{1}H NMR (400 MHz, $CDCl_3$) δ 11.84 (s, 1H), 8.64 (s, 1H), 7.83 (dd, *J* = 7.9, 1.7 Hz, 1H), 7.52 (ddd, *J* = 8.6, 7.2, 1.7 Hz, 1H), 7.48 – 7.39 (m, 4H), 7.22 (d, *J* = 2.8 Hz, 1H), 7.05 (dd, *J* = 8.3, 1.2 Hz, 1H), 6.92 (ddd, *J* = 8.2, 7.2, 1.1 Hz, 1H), 6.89 (tt, *J* = 54.3, 6.1 Hz, 1H).

^{19}F NMR (376 MHz, $CDCl_3$) δ 53.44 – 53.25 (m), 25.25 (dt, *J* = 54.3, 10.3 Hz).

^{13}C NMR (126 MHz, DMSO-*d6*) δ 192.0, 158.1, 134.7 (t, *J* = 3.6 Hz), 133.7, 133.5, 131.2 (2C), 131.0, 130.1, 129.7, 128.1 (2C), 124.3, 122.0 (t, *J* = 3.4 Hz), 118.9, 117.0, 115.6 (tt, *J* = 248.1, 26.0 Hz), 110.6 (tt, *J* = 249.7, 34.3 Hz), 109.00 (t, *J* = 26.1 Hz).

HRMS (ESI): m/z calcd for $C_{19}H_{12}ClF_4NNaO_2$ [M + Na]$^+$, 420.0385; found, 420.0385.

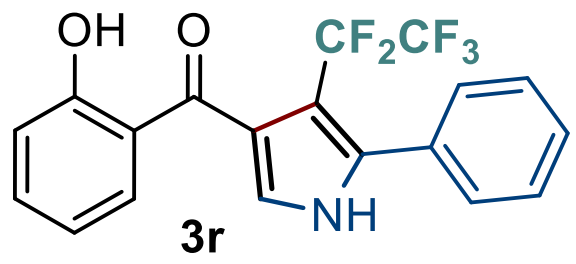

(2-Hydroxyphenyl)(4-(perfluoroethyl)-5-phenyl-1*H*-pyrrol-3-yl)methanone (3r): beige solid (99 mg, 52%), mp 141–143 °C;

^{1}H NMR (400 MHz, $CDCl_3$) δ 12.06 (s, 1H), 8.65 (s, 1H), 7.72 (dd, *J* = 8.1, 1.7 Hz, 1H), 7.50 (ddd, *J* = 8.7, 7.2, 1.8 Hz, 1H), 7.48 – 7.43 (m, 5H), 7.10 (d, *J* = 2.8 Hz, 1H), 7.04 (dd, *J* = 8.3, 1.2 Hz, 1H), 6.89 (ddd, *J* = 8.2, 7.2, 1.2 Hz, 1H).

^{19}F NMR (376 MHz, $CDCl_3$) δ 77.70 (t, *J* = 3.0 Hz), 59.03 (q, *J* = 3.2 Hz).

^{13}C NMR (126 MHz, DMSO-*d6*) δ 192.7, 159.2, 136.7 (t, *J* = 5.3 Hz), 134.3, 131.6, 131.1, 129.8 (2C), 128.8, 128.0, 127.5 (2C), 123.8, 121.9 (t, *J* = 2.1 Hz), 119.2 (qt, *J* = 286.9, 41.0 Hz), 118.9, 117.1, 113.4 (tq, *J* = 252.5, 39.7 Hz), 105.4 (t, *J* = 27.3 Hz).

HRMS (ESI): m/z calcd for $C_{19}H_{13}F_5NO_2$ [M + H]$^+$, 382.0861; found; 382.0860.

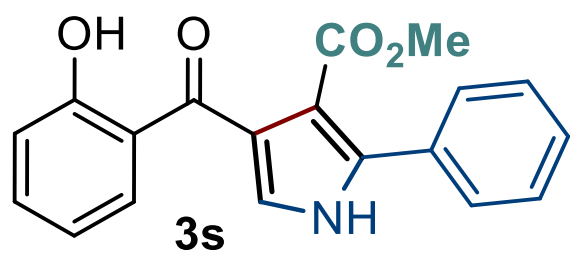

Methyl 4-(2-hydroxybenzoyl)-2-phenyl-1*H*-pyrrole-3-carboxylate (3s): beige solid (59 mg, 37%), mp 188–190 °C;

^{1}H NMR (400 MHz, $CDCl_3$) δ 12.11 (s, 1H), 8.63 (s, 1H), 7.73 (dd, *J* = 8.0, 1.7 Hz, 1H), 7.66 – 7.59 (m, 2H), 7.52 – 7.40 (m, 4H), 7.17 (d, *J* = 2.8 Hz, 1H), 7.04 (dd, *J* = 8.3, 1.1 Hz, 1H), 6.87 (ddd, *J* = 8.2, 7.1, 1.2 Hz, 1H), 3.53 (s, 3H).

^{13}C NMR (126 MHz, DMSO-*d6*) δ 193.7, 165.5, 160.2, 135.0, 134.9, 131.8, 130.6, 128.9, 128.4, 128.1, 127.9, 127.1, 125.2, 123.6, 121.8, 119.0, 117.3, 112.8, 51.3.

HRMS (ESI): m/z calcd for $C_{19}H_{16}NO_4$ [M + H]$^+$, 322.1074; found, 322.1073.

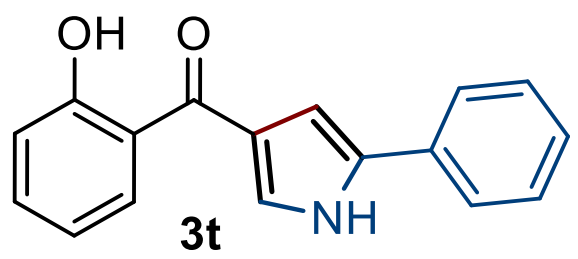

(2-Hydroxyphenyl)(5-phenyl-1*H*-pyrrol-3-yl)methanone (3t): beige solid (59 mg, 45%), mp 188-190 °C [5];

^{1}H NMR (500 MHz, $CDCl_3$) δ 12.17 (s, 1H), 8.94 (s, 1H), 7.99 (dd, *J* = 7.9, 1.7 Hz, 1H), 7.55 – 7.51 (m, 2H), 7.51 – 7.45 (m, 2H), 7.42 (t, *J* = 7.8 Hz, 2H), 7.35 – 7.27 (m, 1H), 7.05 (dd, *J* = 8.5, 1.1 Hz, 1H), 7.01 (dd, *J* = 2.9, 1.6 Hz, 1H), 6.96 – 6.89 (m, 1H).

HRMS (ESI): m/z calcd for $C_{17}H_{14}NO_2$ [M + H]$^+$, 264.1019; found, 264.1019.

iv. Analytical data for the mixture of 2-arylpyrroles 3u and 4u

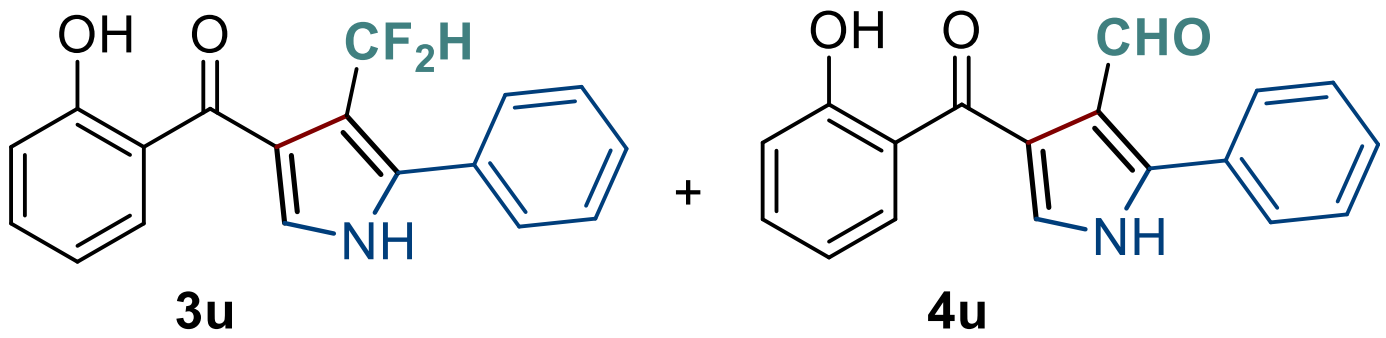

Complex mixture product **3u** and **4u**
ratio: **3u:4u (5:1)**
Total yield: 70mg

(4-(Difluoromethyl)-5-phenyl-1*H*-pyrrol-3-yl)(2-hydroxyphenyl)methanone (3u) and 4-(2-hydroxybenzoyl)-2-phenyl-1*H*-pyrrole-3-carbaldehyde (4u) mixture of products: grey solid (70 mg**).**

^{1}H NMR (400 MHz, $CDCl_3$) δ 12.09 (s, 0.2H), 11.92 (s, 1H), 10.02 (s, 0.2H), 8.87 (s, 0.2H), 8.74 (s, 1H), 7.87 (dd, *J* = 7.8, 1.8 Hz, 1H), 7.74 (d, *J* = 8.0 Hz, 0H), 7.67 – 7.55 (m, 2H), 7.53 – 7.41 (m, 5H), 7.30 – 7.24 (m, 2H), 7.05 (t, *J* = 54.6 Hz, 1H),7.05 (d, *J* = 2.4 Hz, 1H), 6.96 – 6.83 (m, 1H).

^{19}F NMR (376 MHz, $CDCl_3$) δ 56.36 (d, *J* = 54.8 Hz).

^{13}C NMR (126 MHz, DMSO-*d6*) δ 194.7, 192.3, 186.3, 159.6, 157.9, 134.6, 134.4, 133.3, 131.9, 130.9, 130.6, 129.2, 129.0, 128.5, 128.4, 128.3, 128.3, 124.6, 122.5, 119.0, 118.9, 117.2, 116.96 113.20 (t, *J* = 24.1 Hz), 111.98 (t, *J* = 231.0 Hz).

3u: HRMS (ESI): m/z calcd for $C_{18}H_{13}F_2NNaO_2$ [M + Na]$^+$ 336.0807; found, 336.0807.

4u: HRMS (ESI): m/z calcd for $C_{18}H_{14}NO_3$ [M + H]$^+$, 292.0968; found 292.0969.

v. Analytical data for 2-arylpyrroles 6a–d

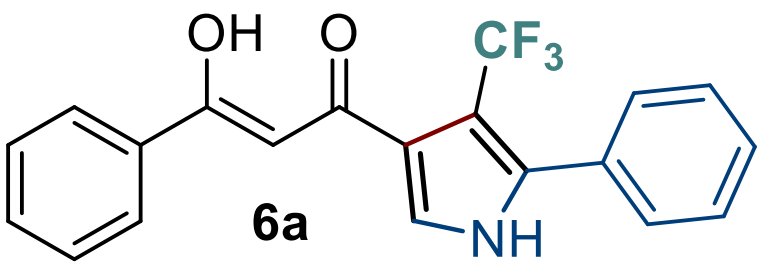

(*Z*)-3-Hydroxy-3-phenyl-1-(5-phenyl-4-(trifluoromethyl)-1*H*-pyrrol-3-yl)prop-2-en-1-one (6a): beige solid (100 mg, 56%), mp 185–187 °C [4];

^{1}H NMR (500 MHz, DMSO-*d6*) δ 16.74 (s, 1H), 12.46 (s, 1H), 8.28 (d, *J* = 3.2 Hz, 1H), 8.09 – 8.05 (m, 2H), 7.63 – 7.58 (m, 1H), 7.57 – 7.53 (m, 2H), 7.51 – 7.45 (m, 5H), 7.06 (s, 1H).

^{19}F NMR (376 MHz, DMSO-*d6*) δ 111.28.

^{13}C NMR (126 MHz, DMSO-*d6*) δ 186.3, 177.5, 136.1 (q, *J* = 4.2 Hz),134.1, 132.1, 130.9, 129.4 (2C), 128.8, 128.7 (2C), 128.2 (2C), 127.3, 126.6 (2C), 123.6 (d, *J* = 267.4 Hz), 121.0, 107.1 (q, *J* = 35.6 Hz), 94.4.

HRMS (ESI): m/z calcd for $C_{20}H_{15}F_3NO_2$ [M + H]$^+$, 358.1049; found, 358.1047.

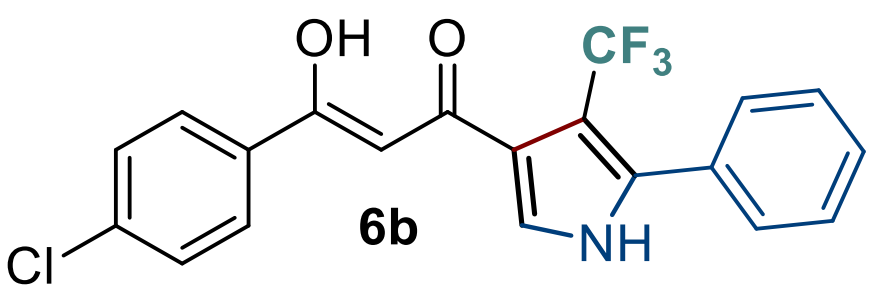

(*Z*)-3-(4-Chlorophenyl)-3-hydroxy-1-(5-phenyl-4-(trifluoromethyl)-1*H*-pyrrol-3-yl)prop-2-en-1-one (6b): beige solid (131 mg, 67%), mp 186–187 °C;

^{1}H NMR (400 MHz, DMSO-*d6*) δ 16.68 (s, 1H), 12.46 (s, 1H), 8.28 (d, *J* = 3.2 Hz, 1H), 8.08 (d, *J* = 8.7 Hz, 2H), 7.62 (d, *J* = 8.6 Hz, 2H), 7.53 – 7.43 (m, 5H), 7.08 (s, 1H).

^{19}F NMR (376 MHz, DMSO-*d6*) δ 111.24.

^{13}C NMR (126 MHz, DMSO-*d6*) δ 186.4, 176.0, 136.8, 136.1 (q, *J* = 4.5, 4.0 Hz), 132.9, 130.9, 130.4, 129.4 (2C), 128.8 (2C), 128.4 (2C), 128.2 (2C), 127.6, 123.5 (q, *J* = 267.7 Hz), 120.9, 107.2 (q, *J* = 35.9 Hz), 94.6.

HRMS (ESI): m/z calcd for $C_{20}H_{12}ClF_3NO_2$ [M-H]$^-$, 390.0514; found, 390.0517

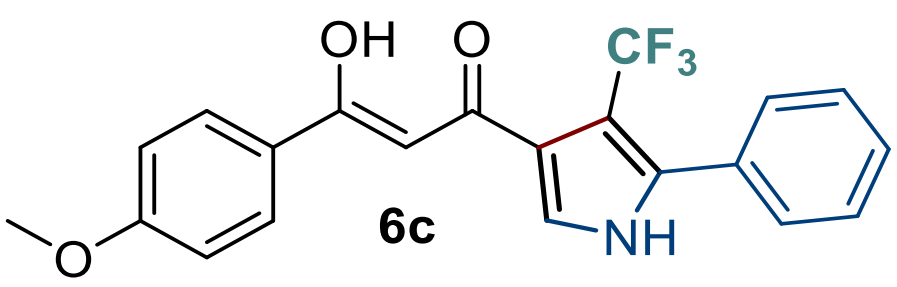

(*Z*)-3-Hydroxy-3-(4-methoxyphenyl)-1-(5-phenyl-4-(trifluoromethyl)-1*H*-pyrrol-3-yl)prop-2-en-1-one (6c): beige solid (122 mg, 63%) mp 143–144 °C;

^{1}H NMR (400 MHz, DMSO-*d*6) δ 16.95 (s, 1H), 12.39 (s, 1H), 8.18 (d, *J* = 3.1 Hz, 1H), 8.03 (d, *J* = 8.6 Hz, 2H), 7.52 – 7.42 (m, 5H), 7.08 (d, *J* = 8.8 Hz, 2H), 6.95 (s, 1H), 3.86 (s, 3H).
^{19}F NMR (376 MHz, DMSO-*d*6) δ 111.48.
^{13}C NMR (126 MHz, DMSO-*d*6) δ 184.9, 178.9, 162.6, 135.8 (q, *J* = 4.0 Hz), 131.0, 129.4 (2C), 128.8, 128.7 (2C), 128.2 (2C), 126.6, 126.5, 123.6 (q, *J* = 268.2 Hz), 120.9, 114.1 (2C), 107.1 (q, *J* = 35.8 Hz), 93.3, 55.5.
HRMS (ESI): m/z calcd for $C_{21}H_{15}F_3NO_3$ $[M-H]^-$, 386.1010; found, 386.1010

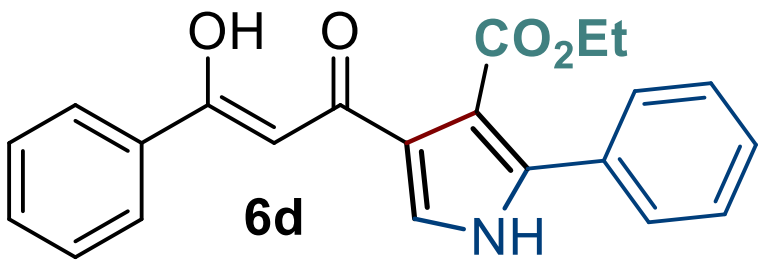

6d

Ethyl (*Z*)-4-(3-hydroxy-3-phenylacryloyl)-2-phenyl-1*H*-pyrrole-3-carboxylate (6d): beige solid (58 mg, 32%), mp 118–120 °C;
^{1}H NMR (500 MHz, $CDCl_3$) δ 16.50 (s, 1H), 9.03 (s, 1H), 7.90 (d, *J* = 7.7 Hz, 2H), 7.54 – 7.47 (m, 1H), 7.45 (d, *J* = 7.6 Hz, 4H), 7.39 – 7.32 (m, 4H), 6.64 (s, 1H), 4.30 (q, *J* = 7.1 Hz, 2H), 1.21 (t, *J* = 7.1 Hz, 3H).
^{13}C NMR (126 MHz, $CDCl_3$) δ 185.0, 180.8, 168.0, 135.1, 135.0, 132.0, 130.9, 128.9 (2C), 128.7 (2C), 128.4, 127.4 (2C), 126.9 (2C), 123.5, 123.2, 112.6, 94.7, 61.7, 14.0.
HRMS (ESI): m/z calcd for $C_{22}H_{20}NO_4$ $[M + H]^+$, 362.1387; found, 362.1389.

2. Copy of ^{1}H, ^{13}C NMR and ^{19}F spectrum of pyrroles 3a–t

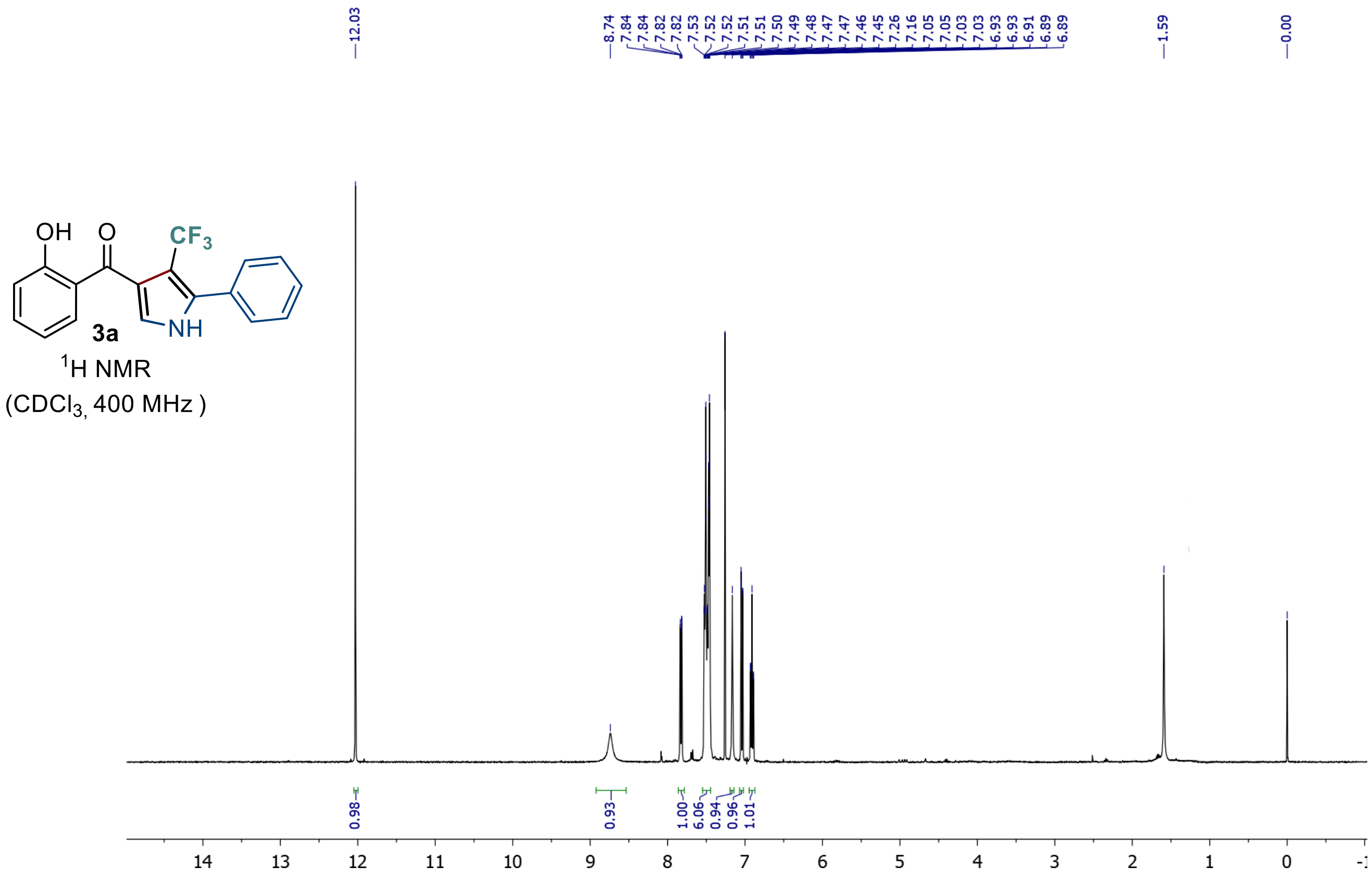

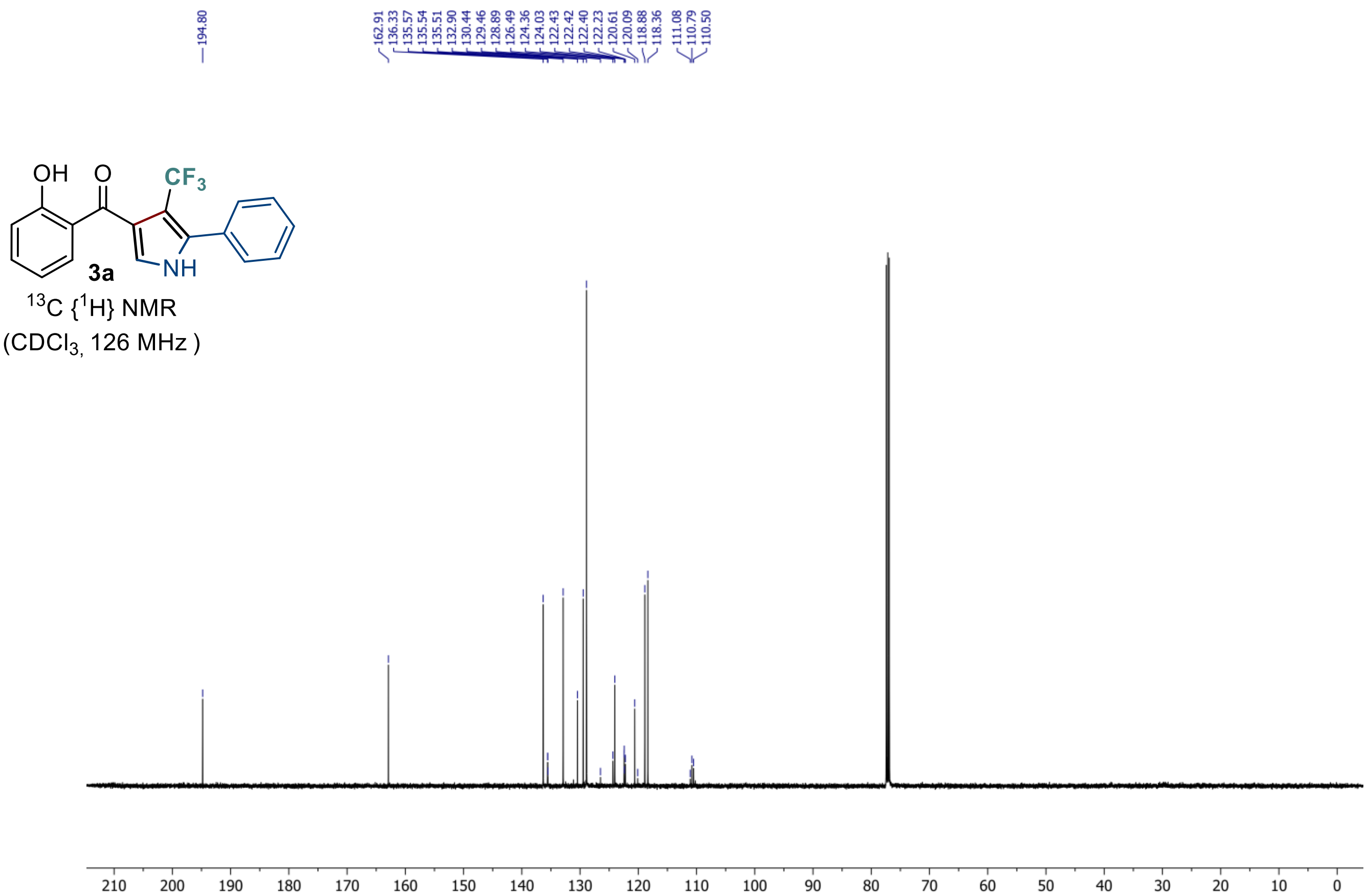

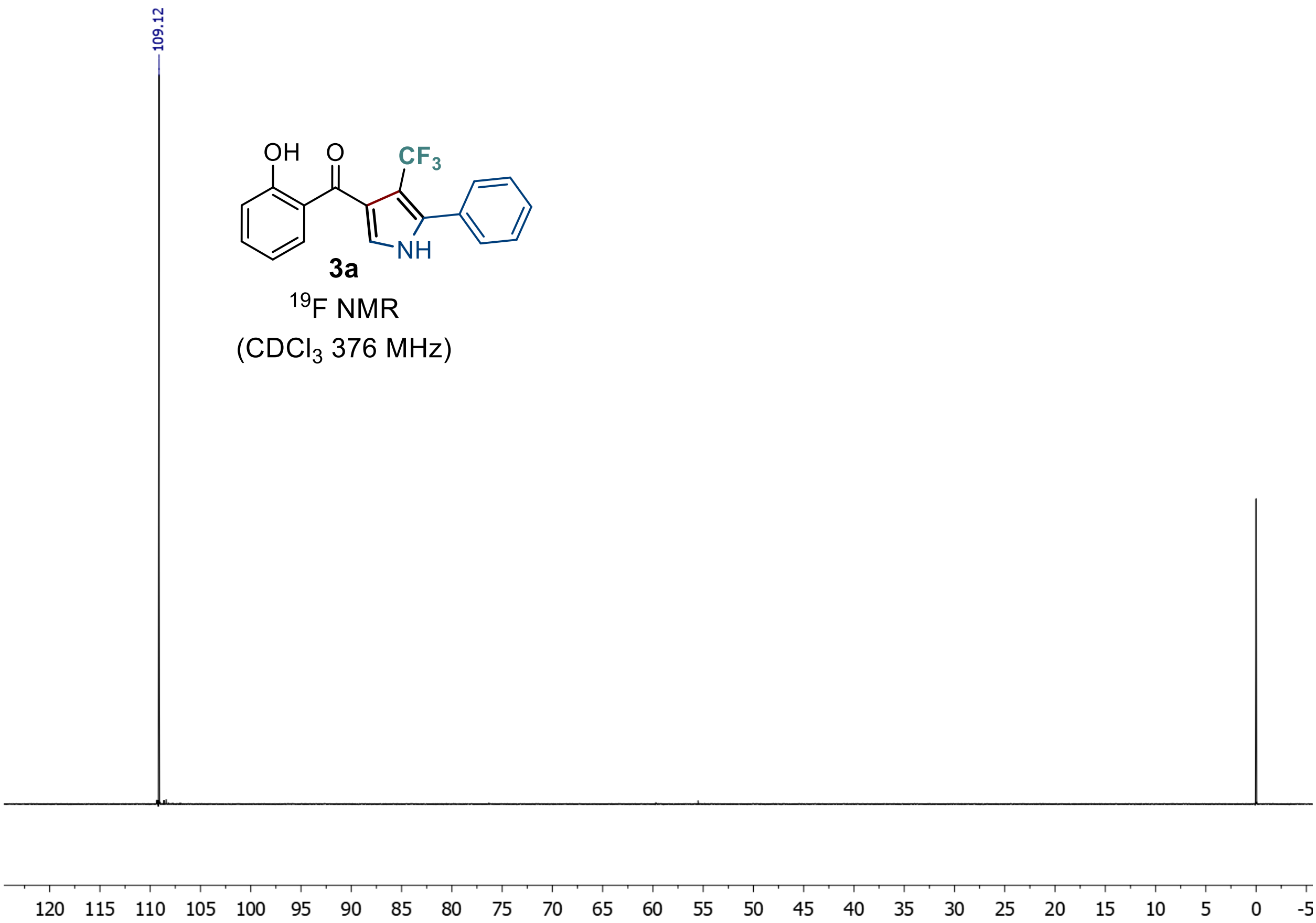
109.12
OH
O
CF3
NH
3a
19F NMR
(CDCl3 376 MHz)
120 115 110 105 100 95 90 85 80 75 70 65 60 55 50 45 40 35 30 25 20 15 10 5 0 -5

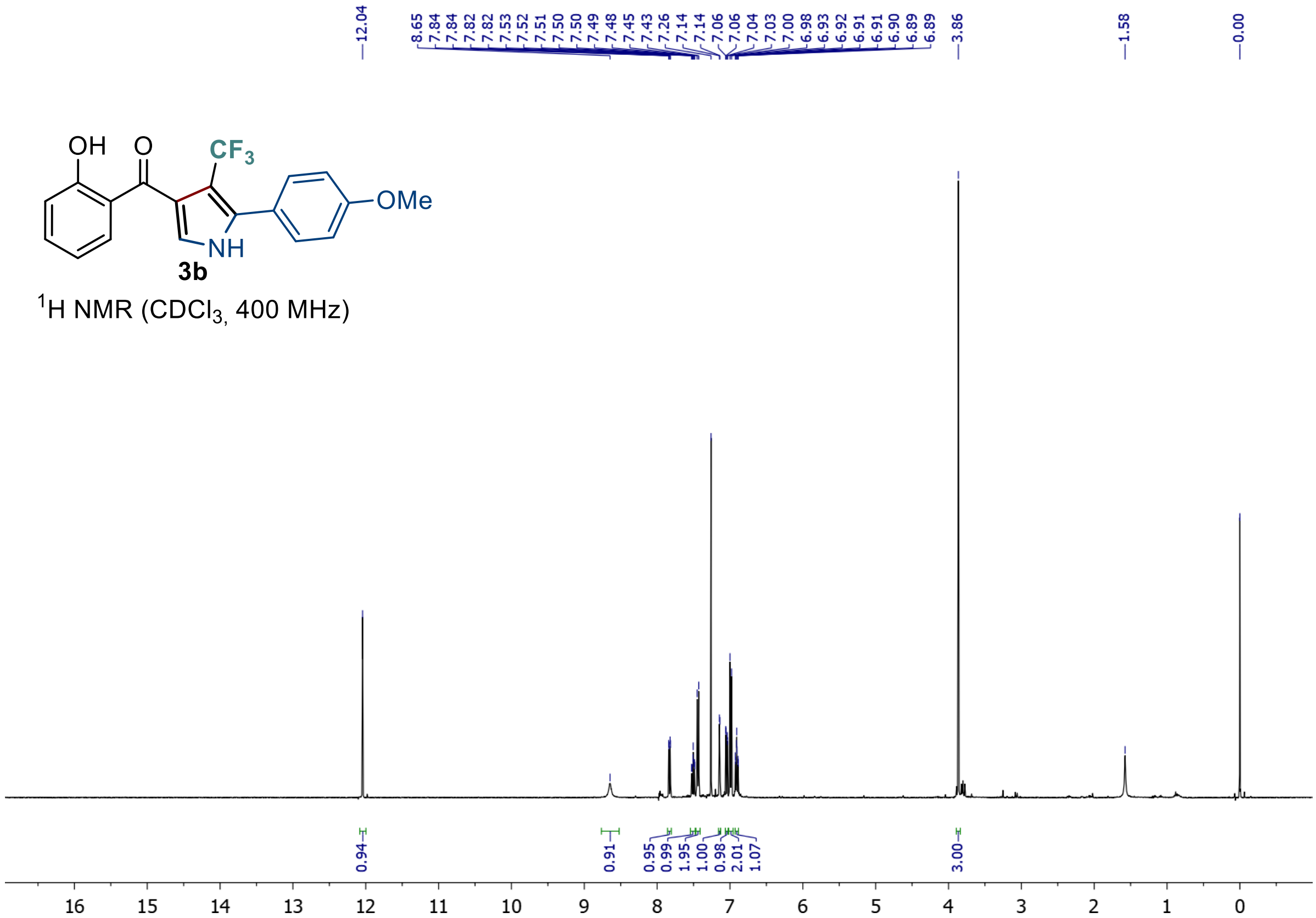
12.04
8.65
7.84
7.84
7.82
7.82
7.53
7.52
7.51
7.50
7.50
7.49
7.48
7.45
7.43
7.26
7.14
7.14
7.06
7.06
7.04
7.03
7.00
6.98
6.93
6.92
6.91
6.91
6.90
6.89
6.89
3.86
1.58
0.00
OH
O
CF3
OMe
NH
3b
1H NMR (CDCl3, 400 MHz)
0.94
0.91
0.95
0.99
1.95
1.00
0.98
2.01
1.07
3.00
16
15
14
13
12
11
10
9
8
7
6
5
4
3
2
1
0

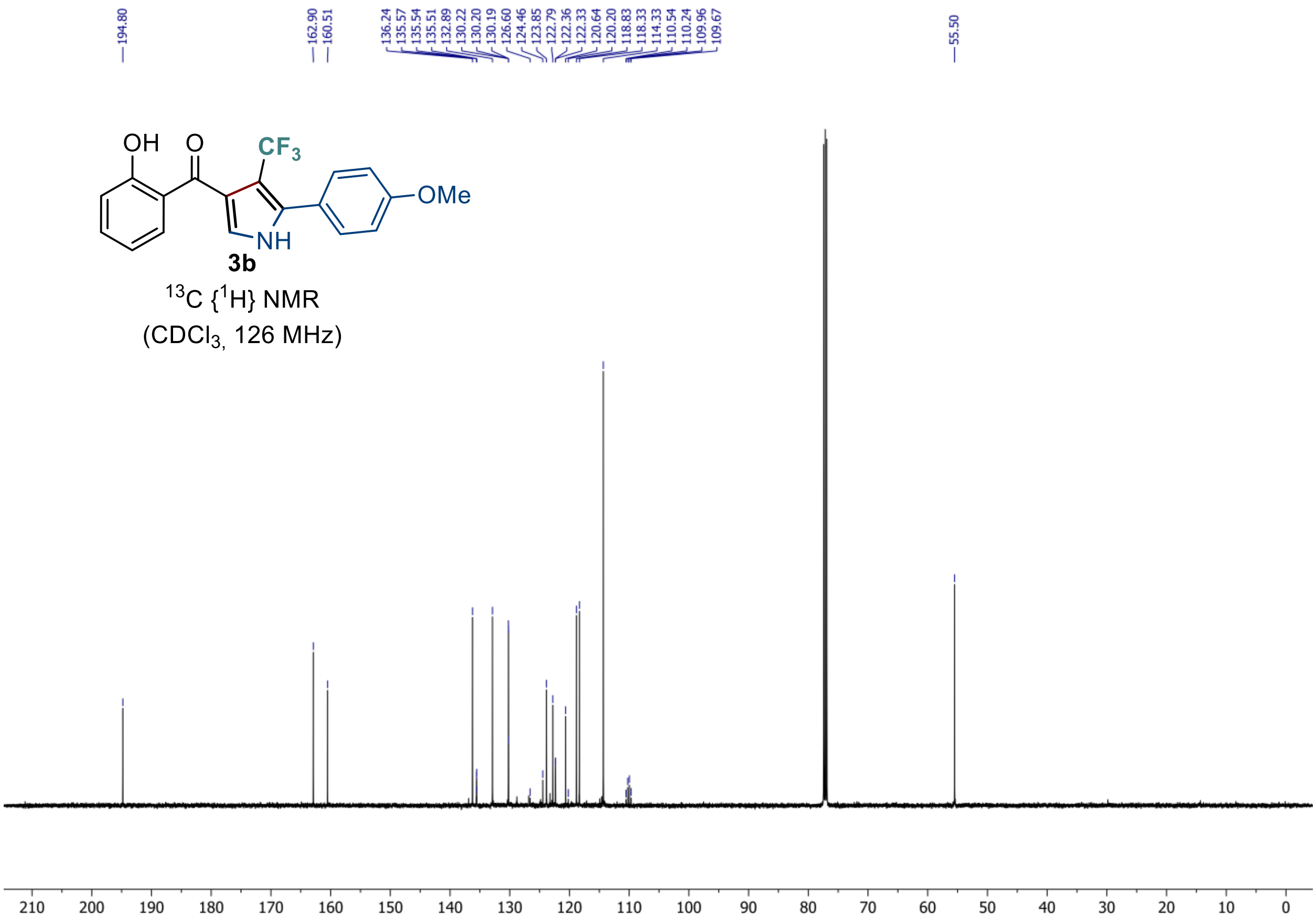
194.80
162.90
160.51
136.24
135.57
135.54
135.51
132.89
130.22
130.20
130.19
126.60
124.46
123.85
122.79
122.36
122.33
120.64
120.20
118.83
118.33
114.33
110.54
110.24
109.96
109.67
55.50
OH
O
CF3
OMe
NH
3b
13C {1H} NMR
(CDCl3, 126 MHz)
210
200
190
180
170
160
150
140
130
120
110
100
90
80
70
60
50
40
30
20
10
0

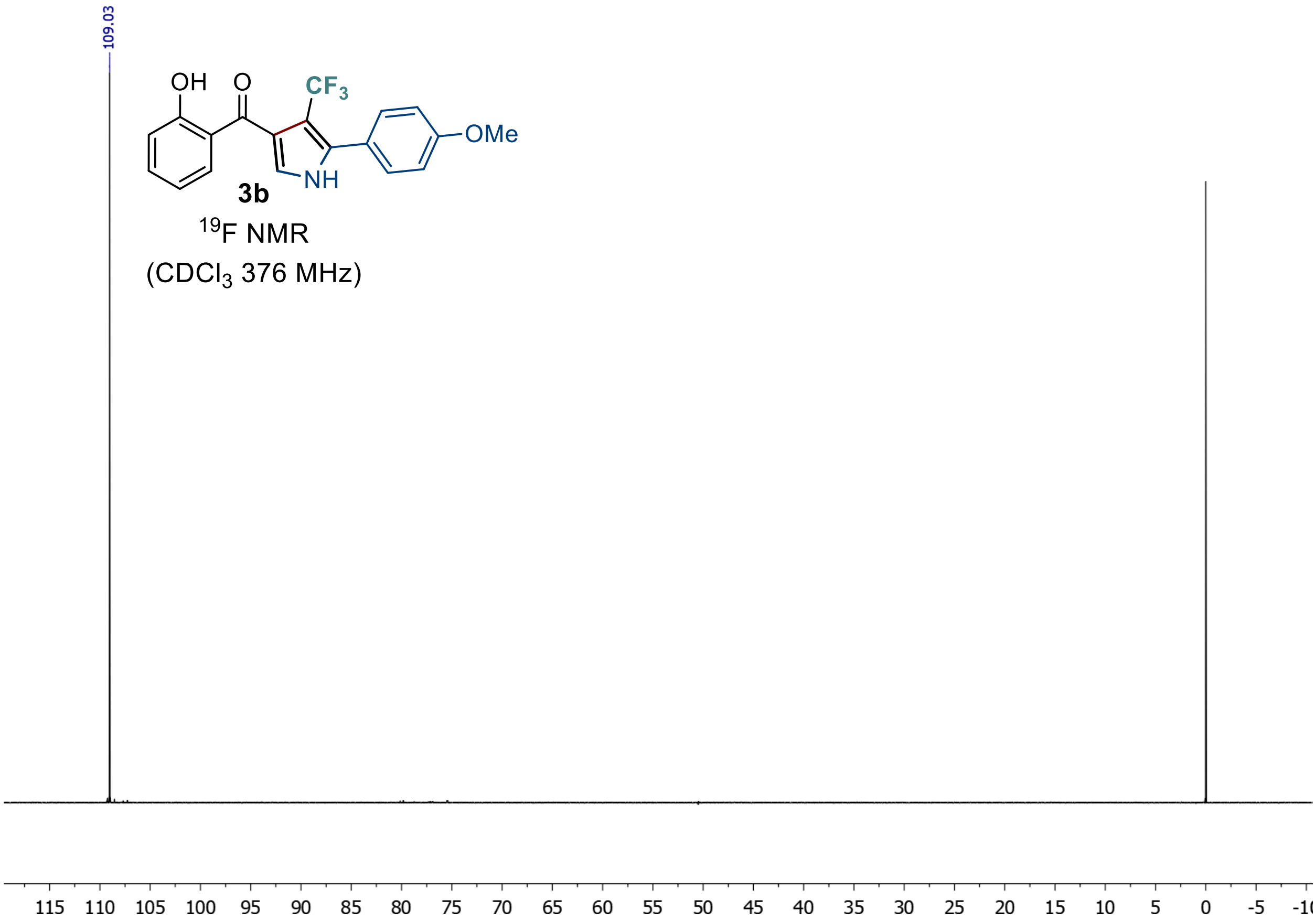
109.03
OH
O
CF3
OMe
NH
3b
19F NMR
(CDCl3 376 MHz)
115
110
105
100
95
90
85
80
75
70
65
60
55
50
45
40
35
30
25
20
15
10
5
0
-5

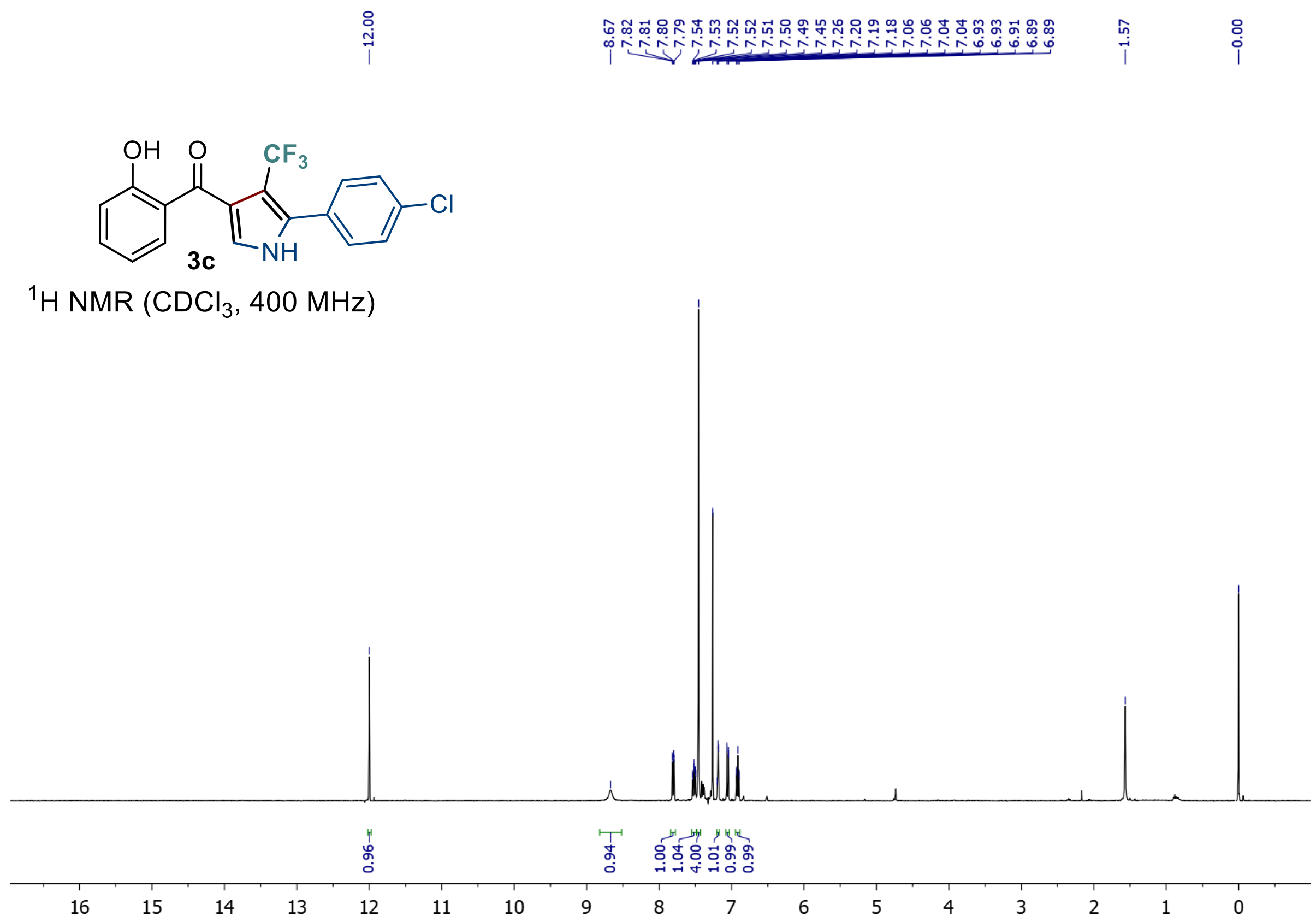
OH
O
CF3
Cl
NH
3c
1H NMR (CDCl3, 400 MHz)

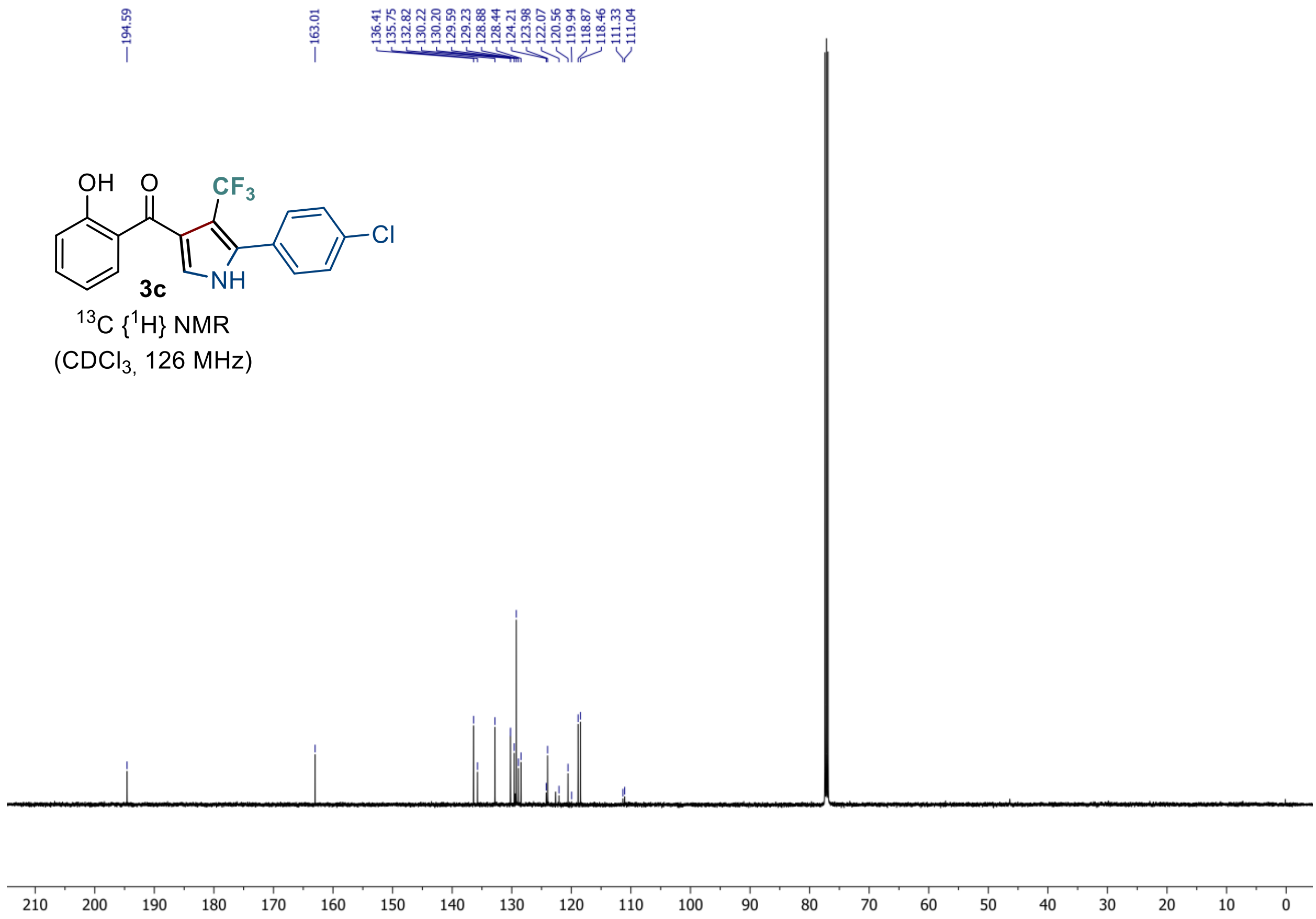
OH
O
CF3
Cl
NH
3c
13C {1H} NMR
(CDCl3, 126 MHz)

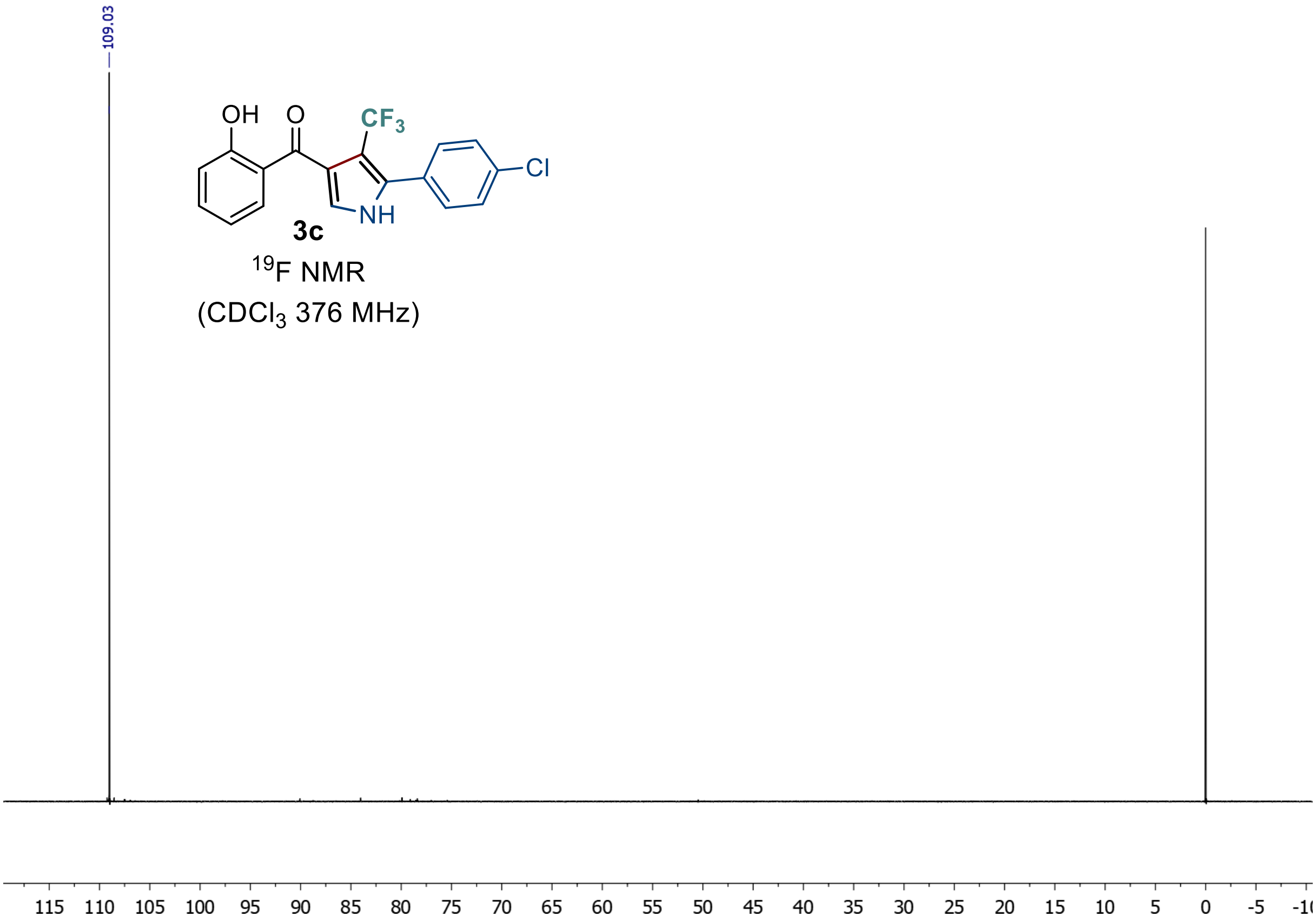

-109.03
OH
O
CF3
Cl
NH
3c
19F NMR
(CDCl3 376 MHz)
115 110 105 100 95 90 85 80 75 70 65 60 55 50 45 40 35 30 25 20 15 10 5 0 -5 -10

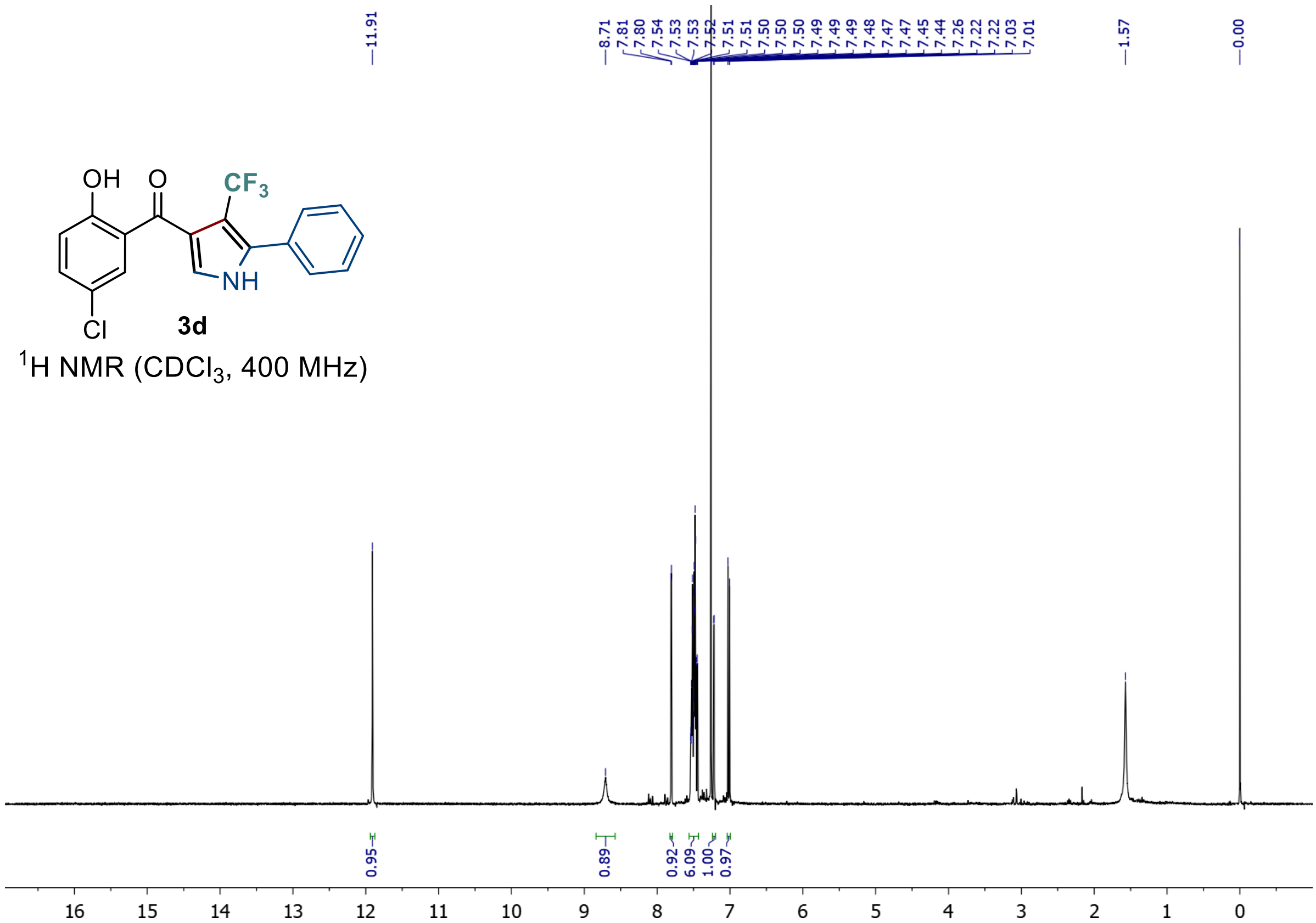
OH O CF3
NH
Cl
3d
1H NMR (CDCl3, 400 MHz)
11.91
8.71 7.81 7.80 7.54 7.53 7.53 7.52 7.51 7.51 7.50 7.50 7.50 7.49 7.49 7.49 7.48 7.47 7.47 7.45 7.44 7.26 7.22 7.22 7.22 7.03 7.01
1.57
0.00
0.95 0.89 0.92 6.09 1.00 0.97
16 15 14 13 12 11 10 9 8 7 6 5 4 3 2 1 0

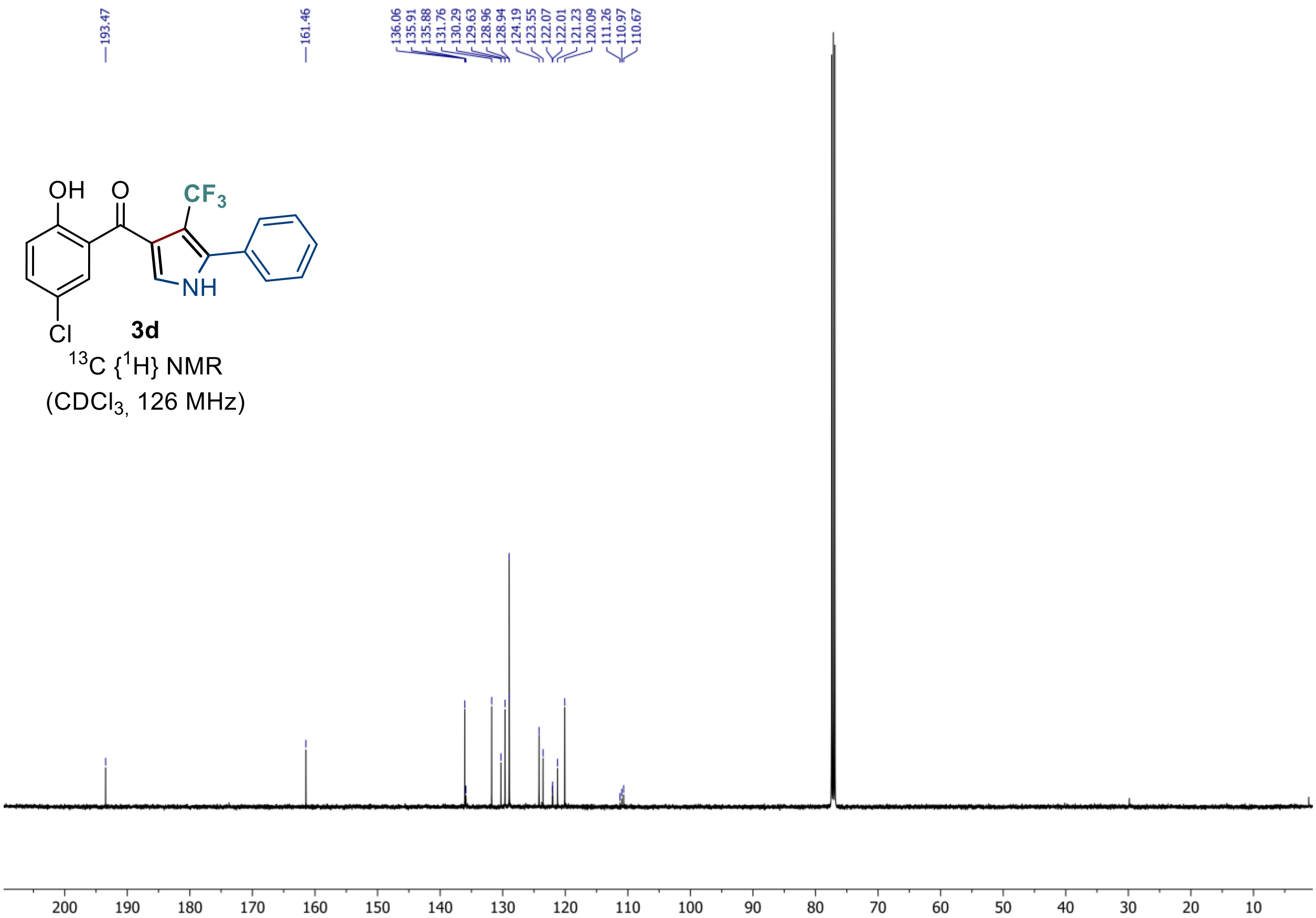
193.47
161.46
136.06 135.91 135.88 131.76 130.29 129.63 128.96 128.94 124.19 123.55 122.07 122.01 121.23 120.09 111.26 110.97 110.67
OH O CF3
NH
Cl
3d
13C {1H} NMR
(CDCl3, 126 MHz)
200 190 180 170 160 150 140 130 120 110 100 90 80 70 60 50 40 30 20 10

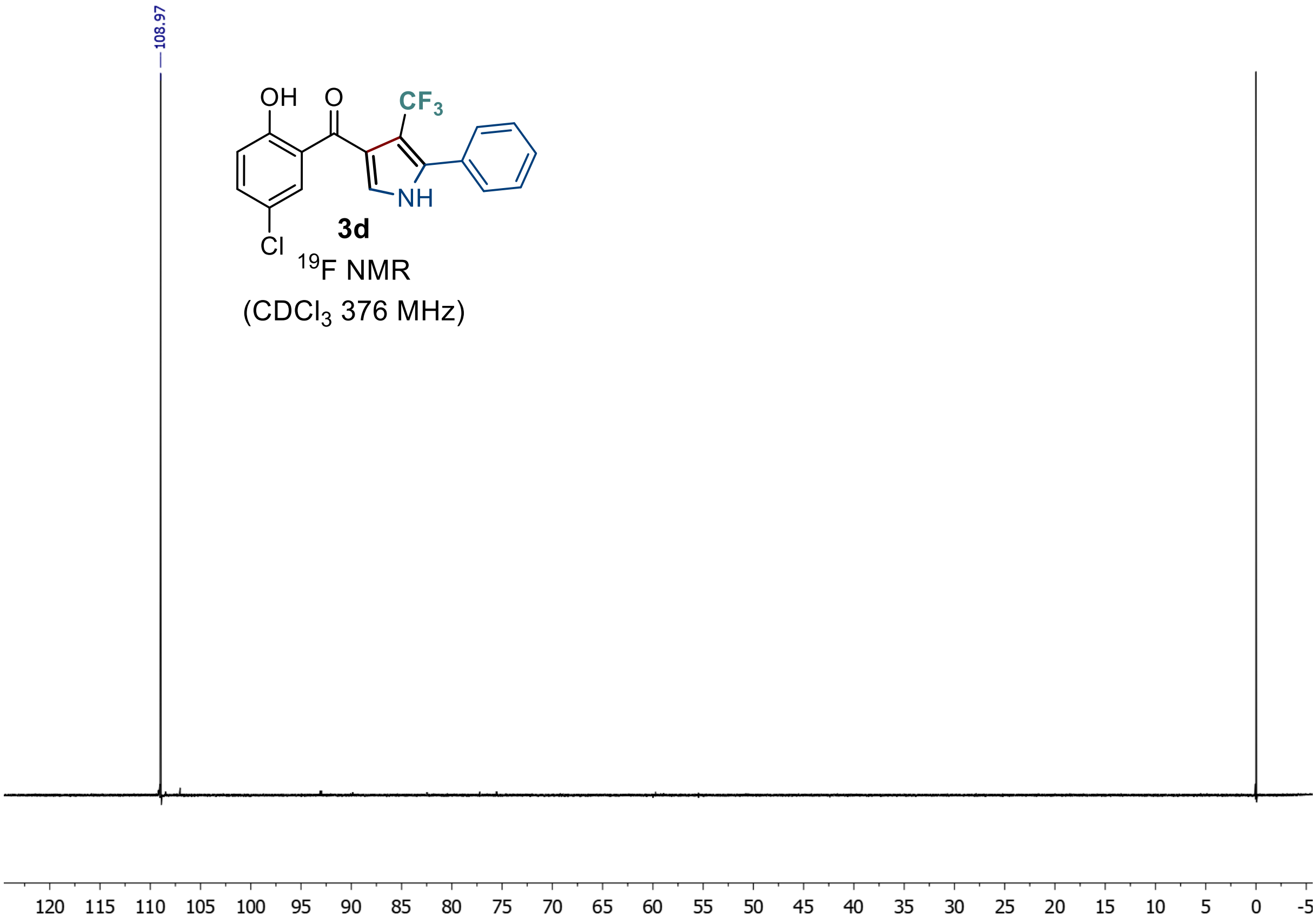

108.97
OH
O
CF3
NH
Cl
3d
19F NMR
(CDCl3 376 MHz)
120 115 110 105 100 95 90 85 80 75 70 65 60 55 50 45 40 35 30 25 20 15 10 5 0 -5

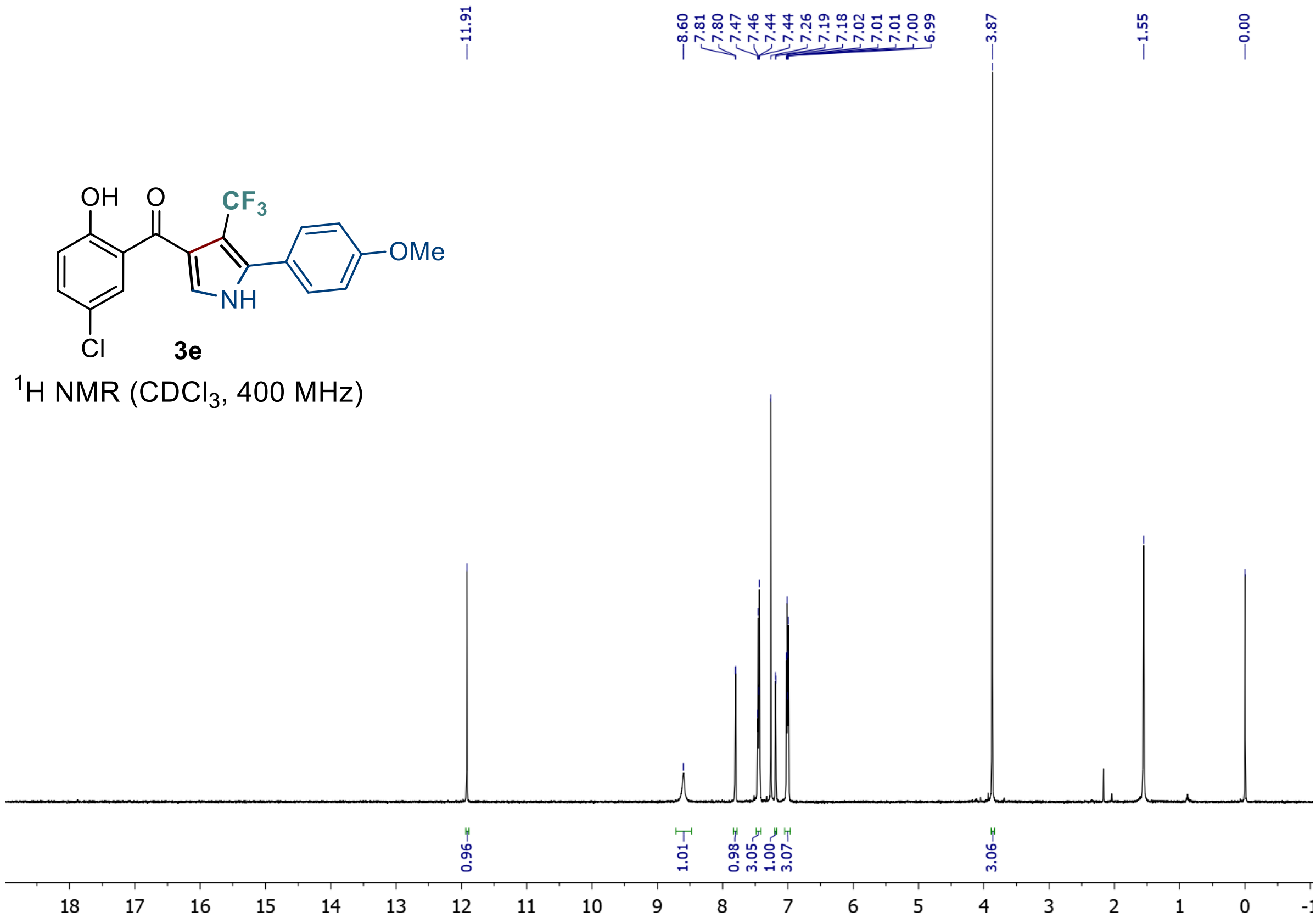
OH
O
CF3
OMe
NH
Cl
3e
1H NMR (CDCl3, 400 MHz)
11.91
8.60
7.81
7.80
7.47
7.46
7.44
7.44
7.26
7.19
7.18
7.02
7.01
7.01
7.00
6.99
3.87
1.55
0.00
0.96
1.01
0.98
3.05
1.00
3.07
3.06
18
17
16
15
14
13
12
11
10
9
8
7
6
5
4
3
2
1
0

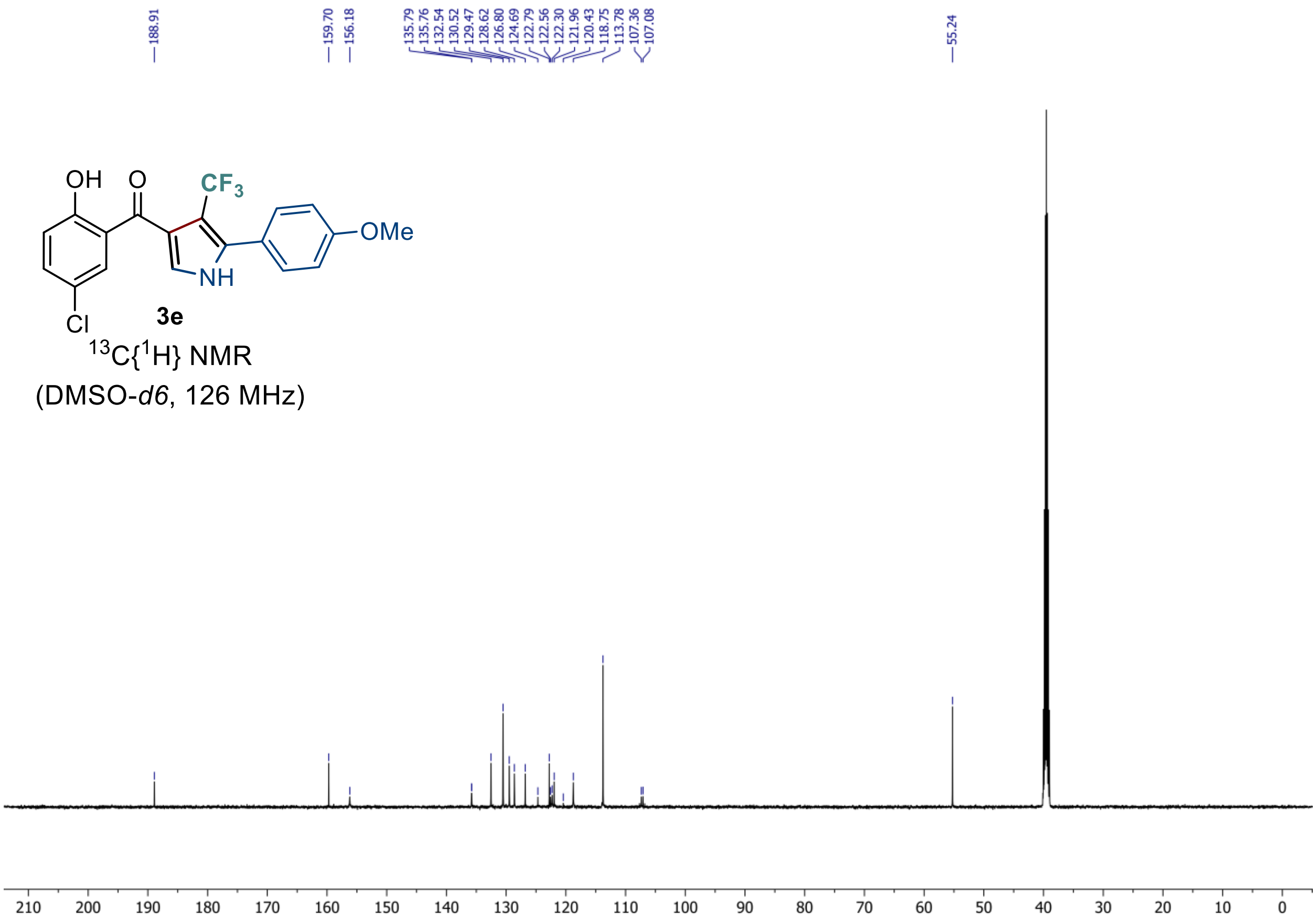
188.91
159.70
156.18
135.79
135.76
132.54
130.52
129.47
128.62
126.80
124.69
122.79
122.56
122.30
121.96
120.43
118.75
113.78
107.36
107.08
55.24
OH
O
CF3
OMe
NH
Cl
3e
13C{1H} NMR
(DMSO-d6, 126 MHz)
210
200
190
180
170
160
150
140
130
120
110
100
90
80
70
60
50
40
30
20
10
0

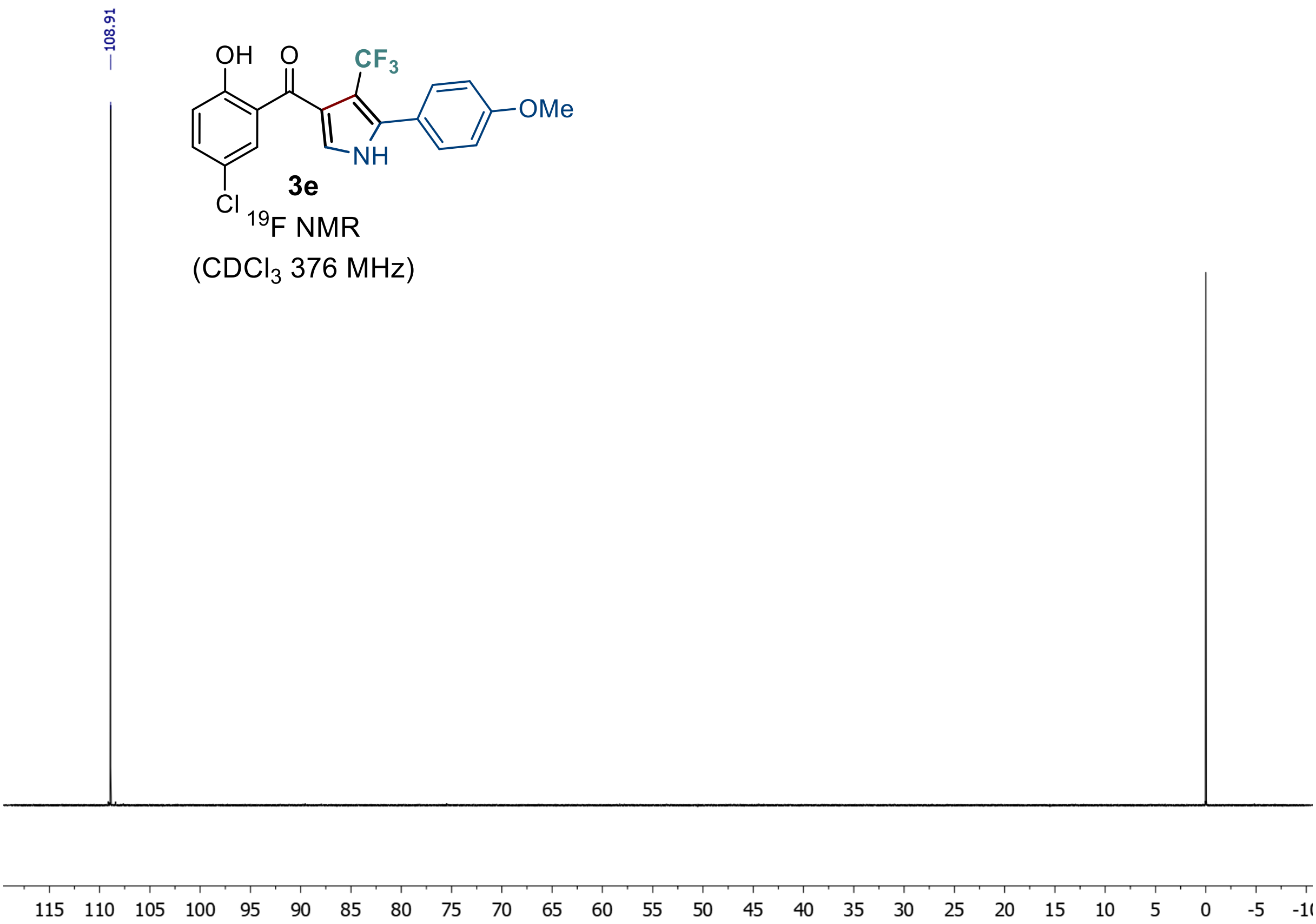

— 108.91
OH
O
CF3
OMe
NH
Cl
3e
19F NMR
(CDCl3 376 MHz)
115
110
105
100
95
90
85
80
75
70
65
60
55
50
45
40
35
30
25
20
15
10
5
0
-5

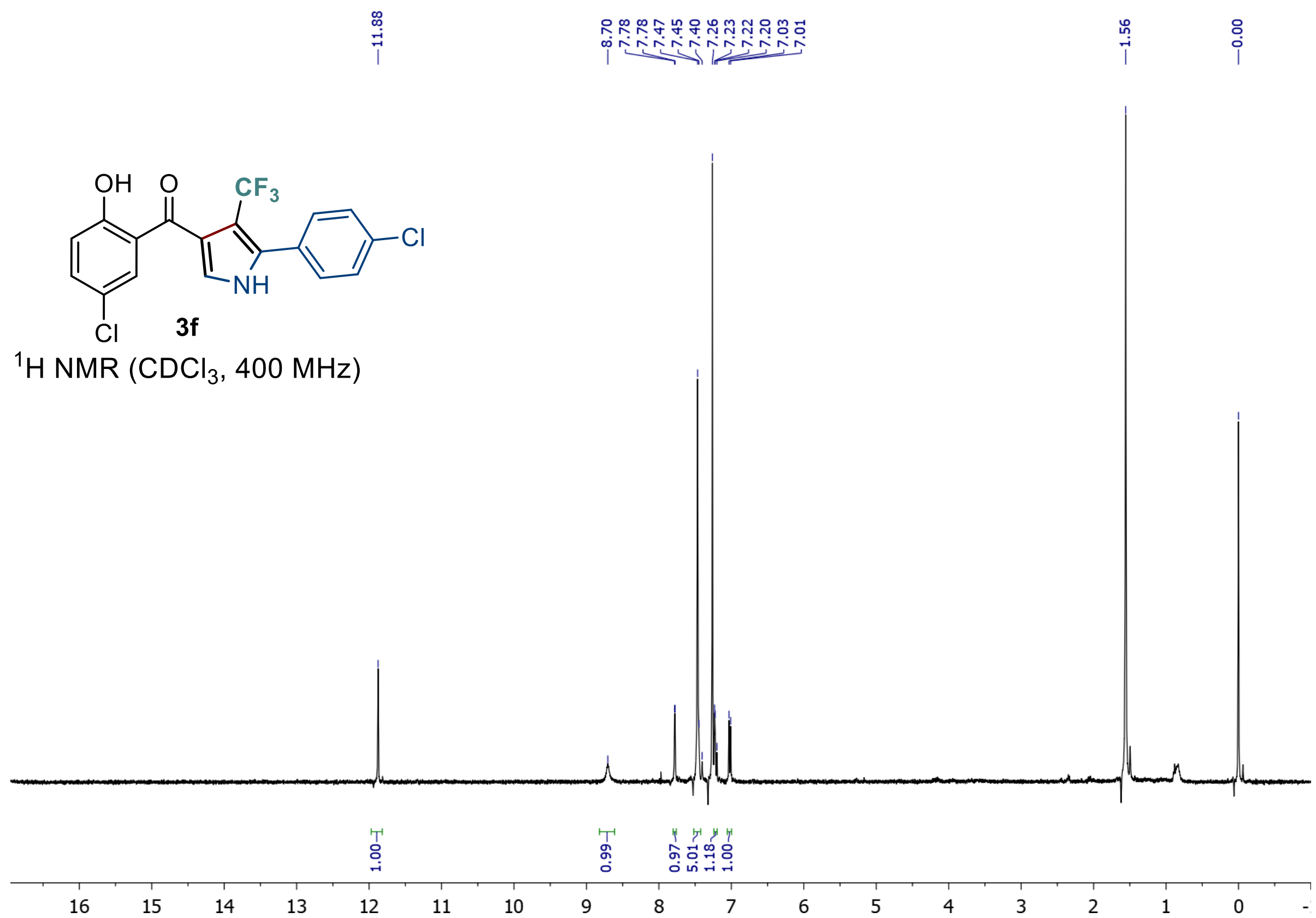

11.88
8.70
7.78
7.78
7.47
7.45
7.40
7.26
7.23
7.22
7.20
7.03
7.01
1.56
0.00
OH
O
CF3
Cl
NH
Cl
3f
1H NMR (CDCl3, 400 MHz)
1.00
0.99
0.97
5.01
1.18
1.00
16 15 14 13 12 11 10 9 8 7 6 5 4 3 2 1 0

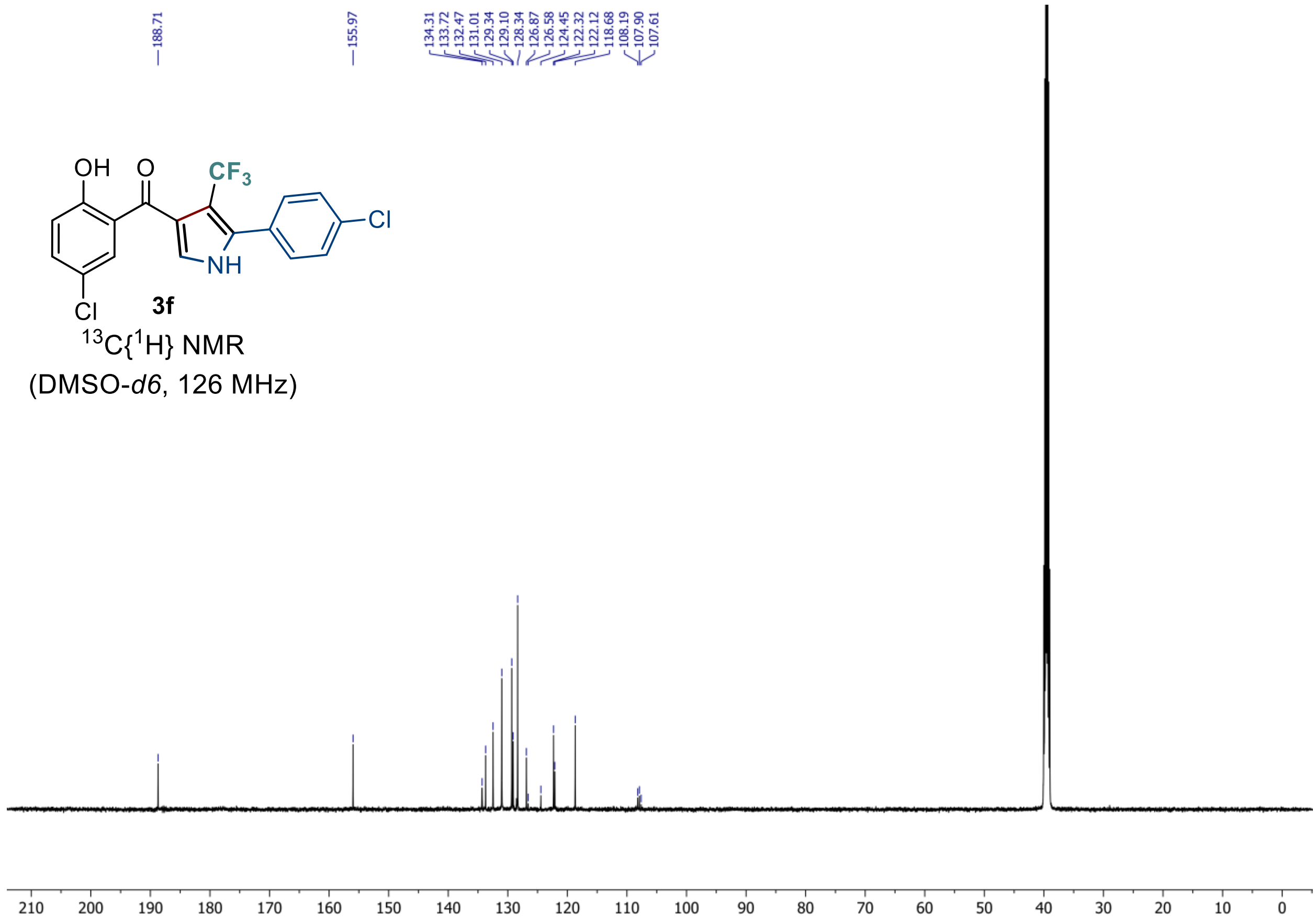

188.71
155.97
134.31
133.72
132.47
131.01
129.34
129.10
128.34
126.87
126.58
124.45
122.32
122.12
118.68
108.19
107.90
107.61
OH
O
CF3
Cl
NH
Cl
3f
13C{1H} NMR
(DMSO-d6, 126 MHz)
210 200 190 180 170 160 150 140 130 120 110 100 90 80 70 60 50 40 30 20 10 0

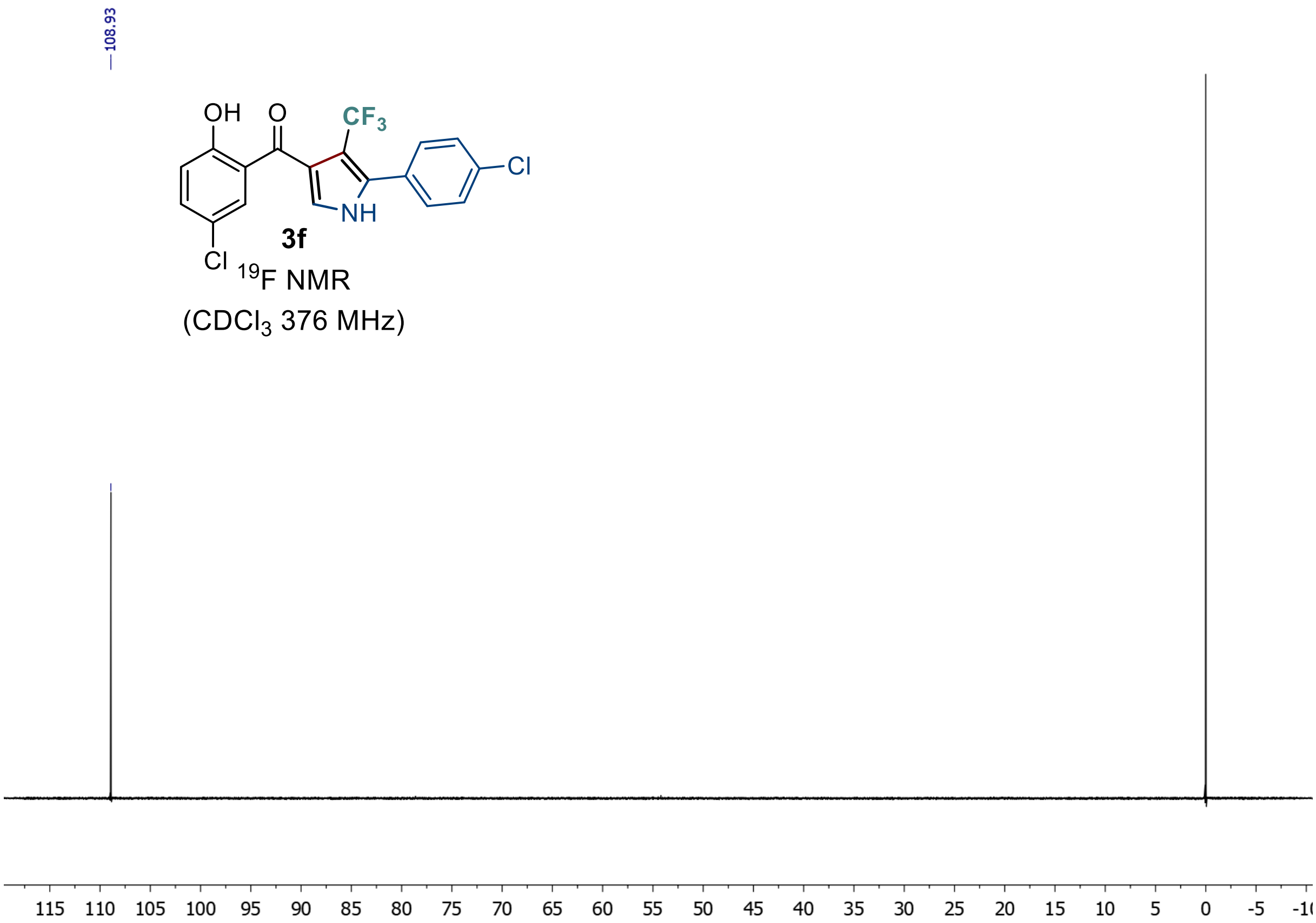

—108.93
OH
O
CF3
Cl
NH
Cl
3f
19F NMR
(CDCl3 376 MHz)
115 110 105 100 95 90 85 80 75 70 65 60 55 50 45 40 35 30 25 20 15 10 5 0 -5

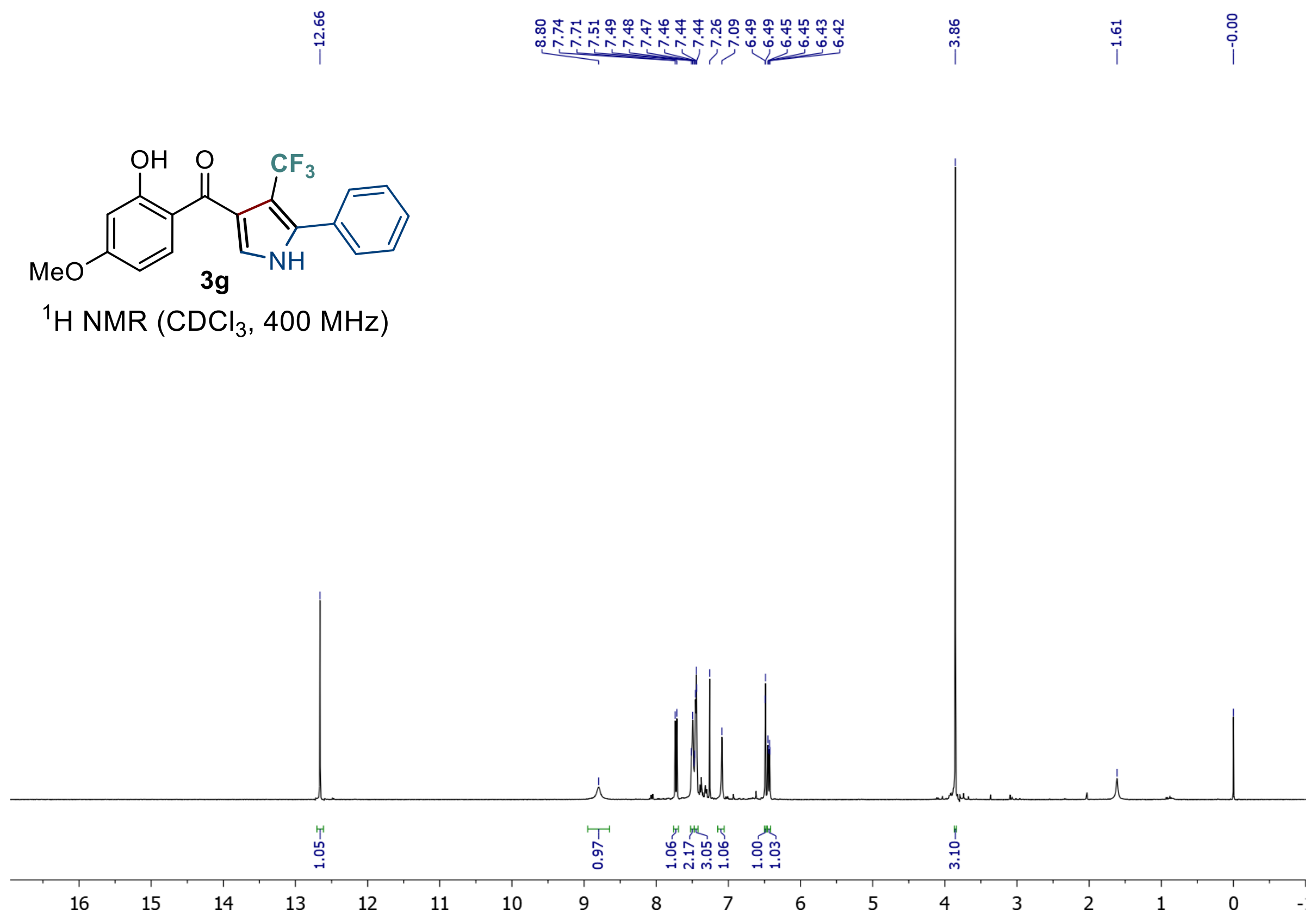

12.66
8.80
7.74
7.71
7.51
7.49
7.48
7.47
7.46
7.44
7.44
7.26
7.09
6.49
6.49
6.45
6.45
6.43
6.42
3.86
1.61
0.00
OH
O
CF3
MeO
NH
3g
1H NMR (CDCl3, 400 MHz)
1.05
0.97
1.06
2.17
3.05
1.06
1.00
1.03
3.10
16 15 14 13 12 11 10 9 8 7 6 5 4 3 2 1 0

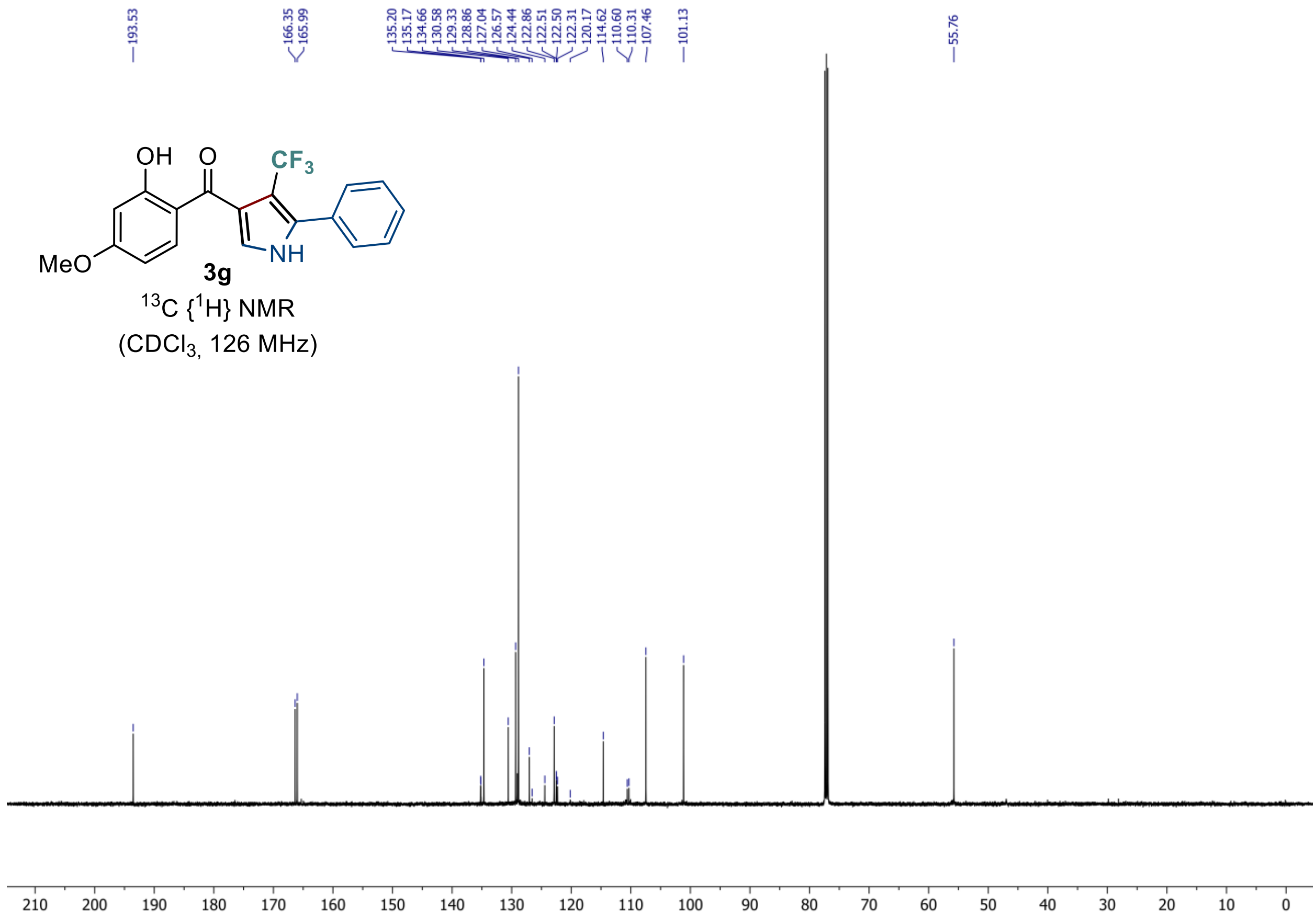

193.53
166.35
165.99
135.20
135.17
134.66
130.58
129.33
128.86
127.04
126.57
124.44
122.86
122.51
122.50
122.31
120.17
114.62
110.60
110.31
107.46
101.13
55.76
OH
O
CF3
MeO
NH
3g
13C {1H} NMR
(CDCl3, 126 MHz)
210 200 190 180 170 160 150 140 130 120 110 100 90 80 70 60 50 40 30 20 10 0

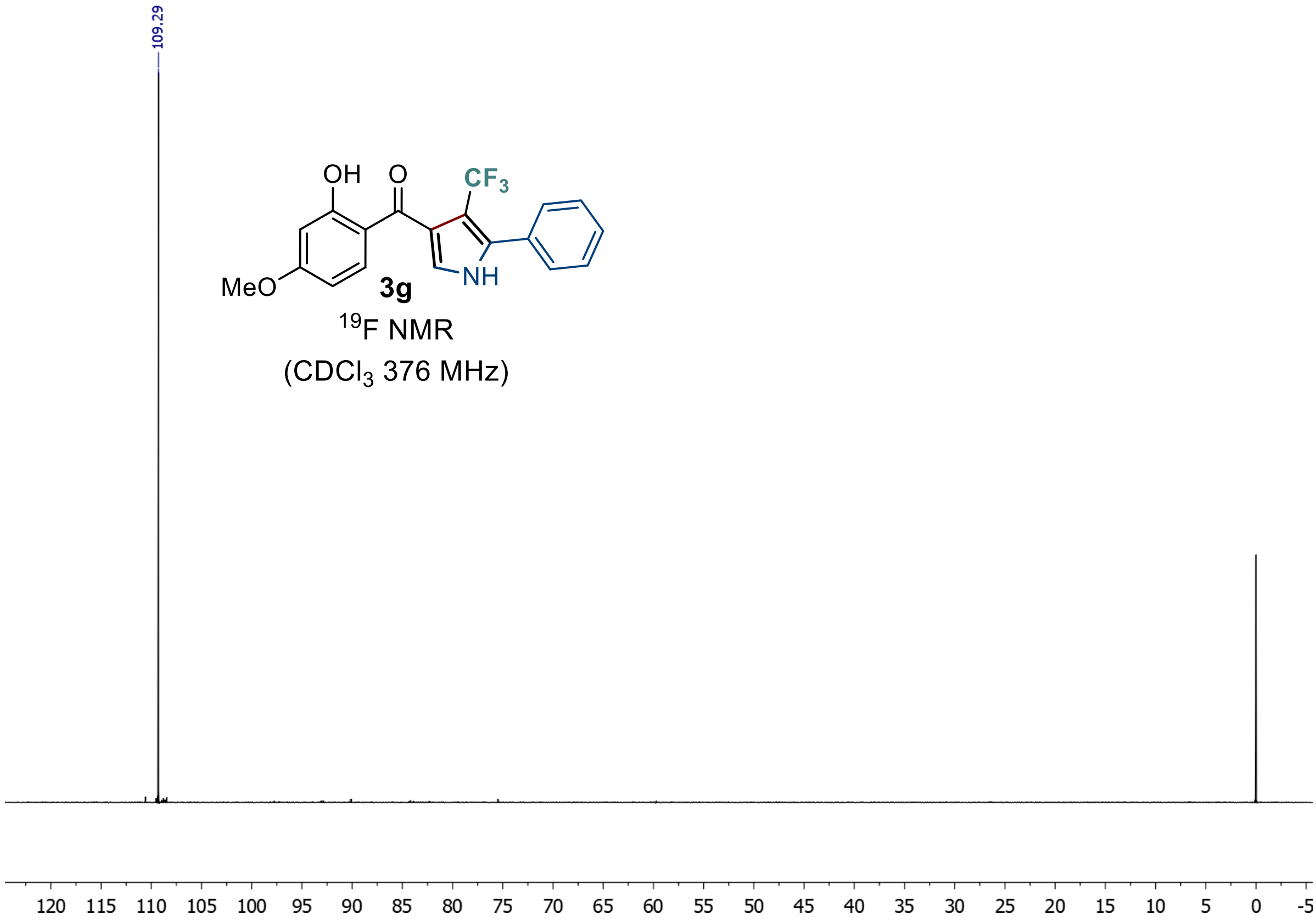

109.29
OH
O
CF3
MeO
NH
3g
19F NMR
(CDCl3 376 MHz)
120 115 110 105 100 95 90 85 80 75 70 65 60 55 50 45 40 35 30 25 20 15 10 5 0 -5

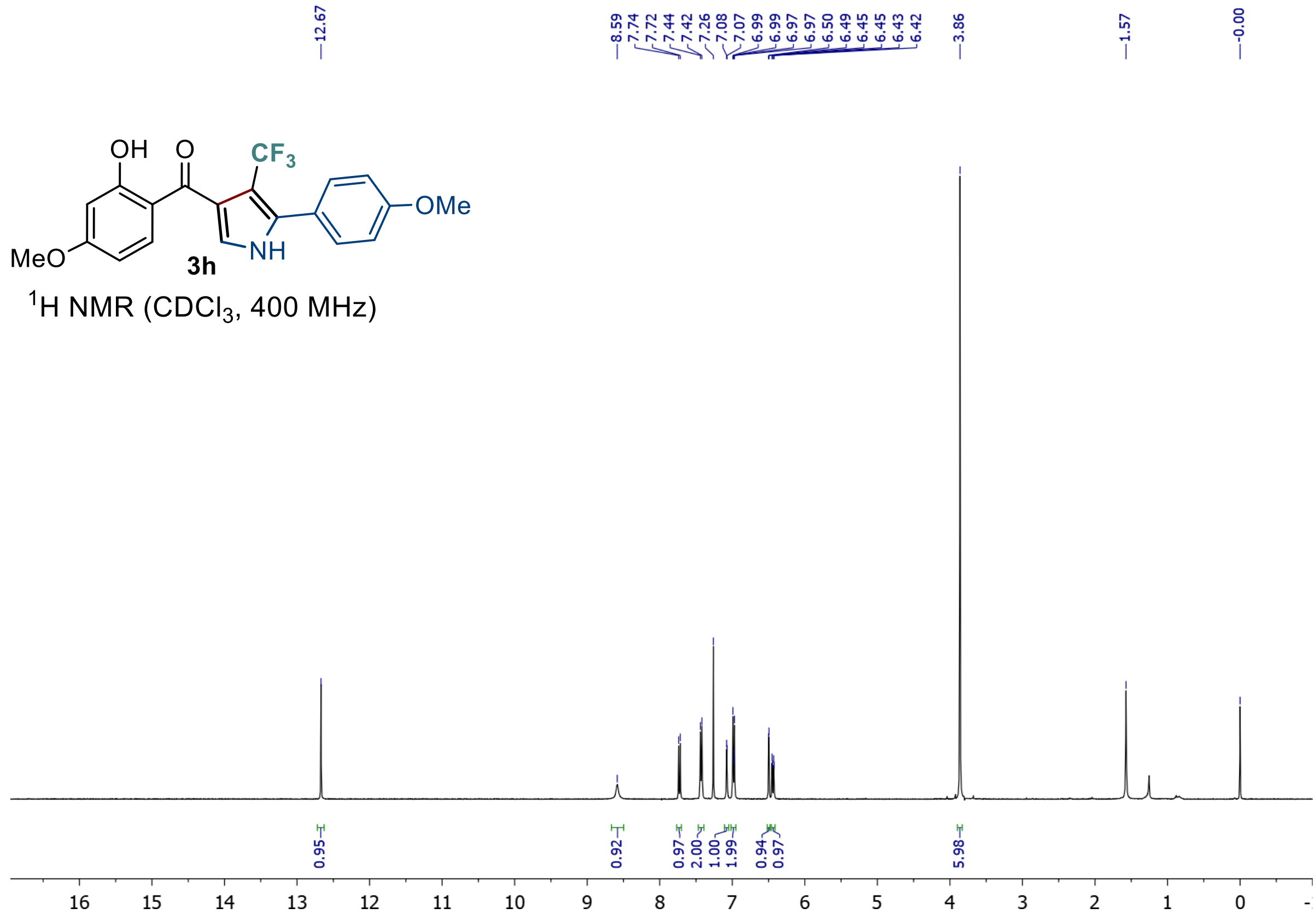
12.67
8.59 7.74 7.72 7.44 7.42 7.26 7.08 7.07 6.99 6.99 6.97 6.97 6.50 6.49 6.45 6.45 6.43 6.42
3.86
1.57
-0.00
OH O CF3
OMe
MeO
NH
3h
1H NMR (CDCl3, 400 MHz)
0.95 0.92 0.97 2.00 1.00 1.99 0.94 0.97 5.98
16 15 14 13 12 11 10 9 8 7 6 5 4 3 2 1 0

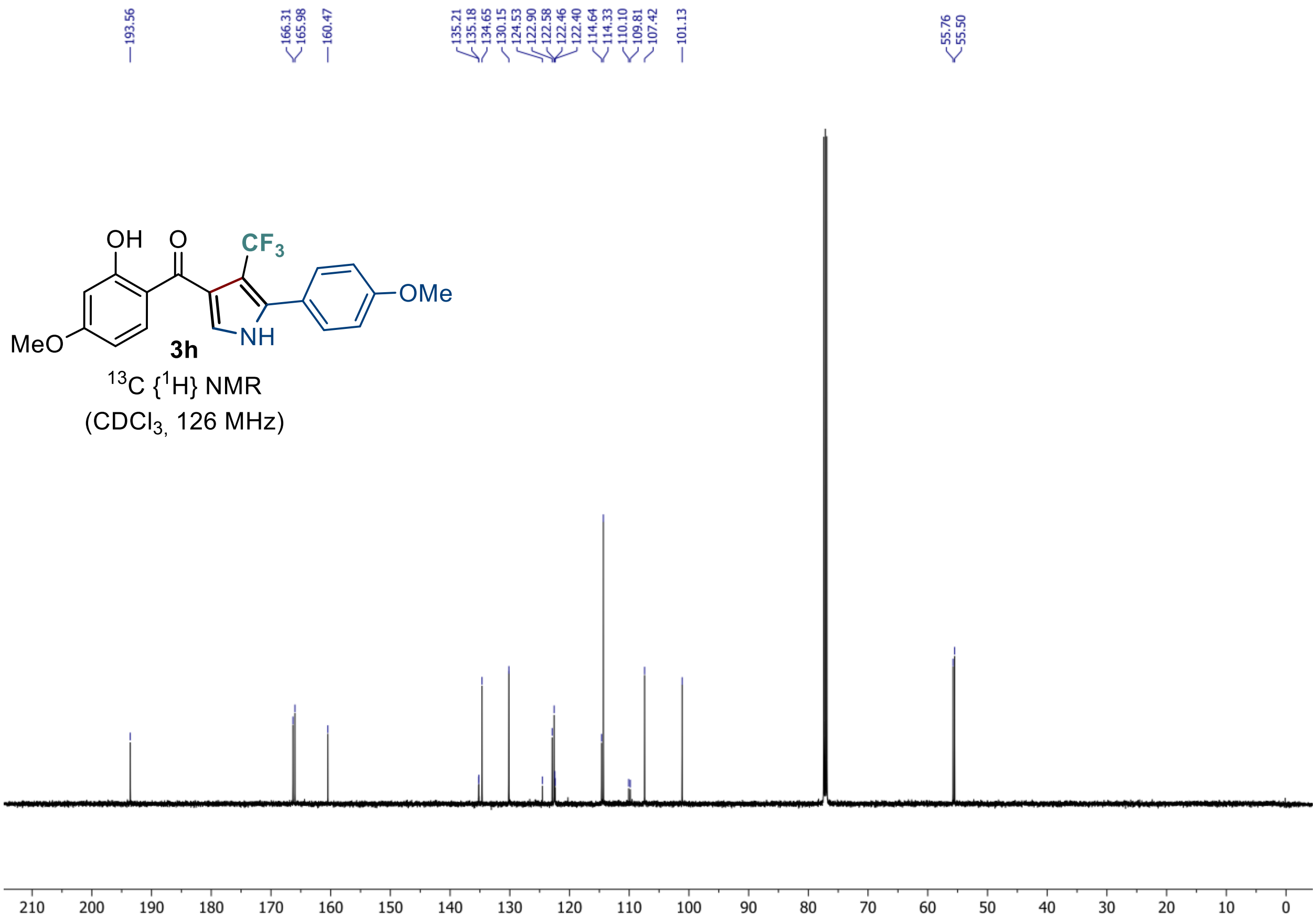
193.56
166.31 165.98
160.47
135.21 135.18 134.65 130.15 124.53 122.90 122.58 122.46 122.40 114.64 114.33 110.10 109.81 107.42
101.13
55.76 55.50
OH O CF3
OMe
MeO
NH
3h
13C {1H} NMR
(CDCl3, 126 MHz)
210 200 190 180 170 160 150 140 130 120 110 100 90 80 70 60 50 40 30 20 10 0

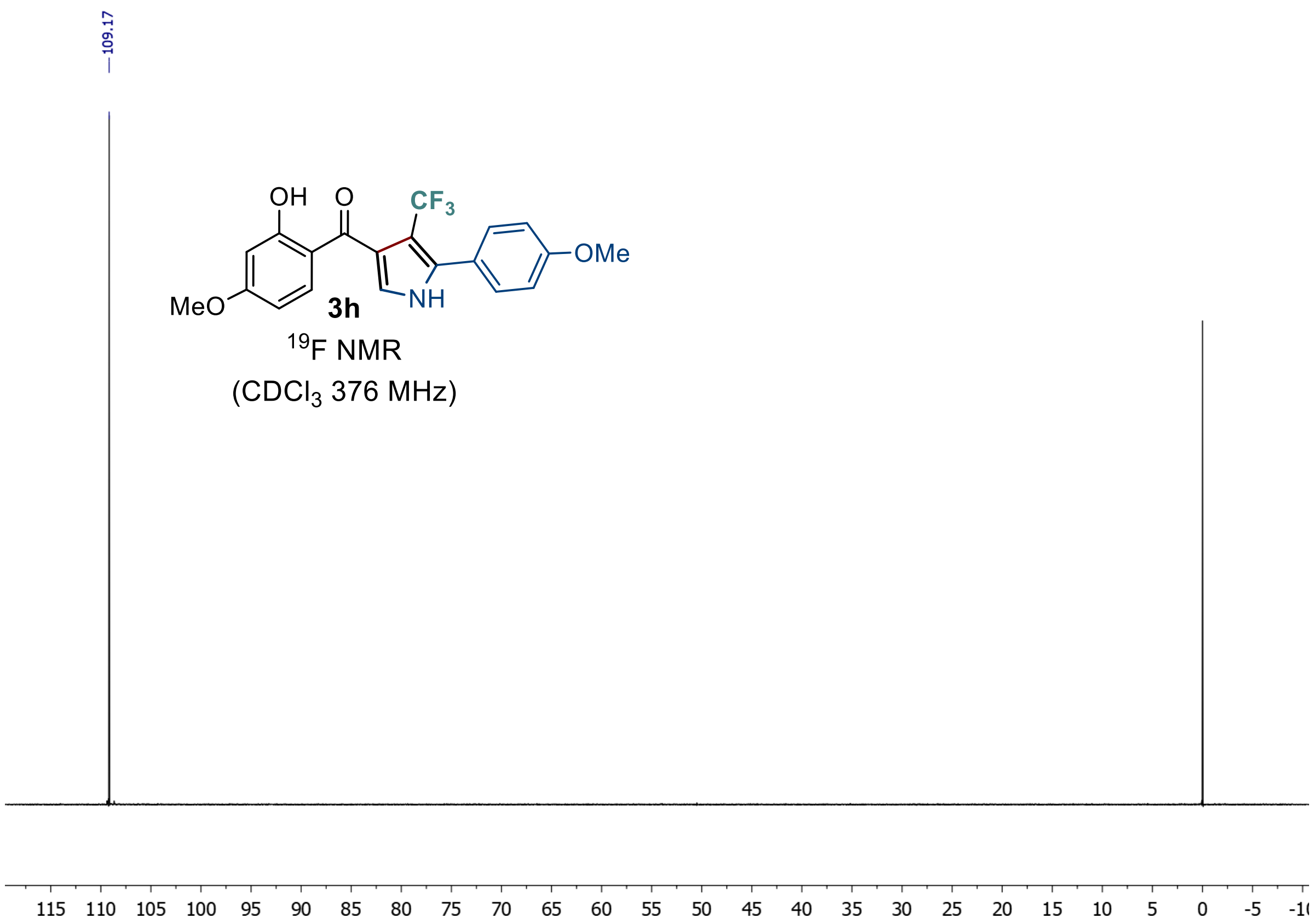

109.17
OH
O
CF3
OMe
MeO
NH
3h
19F NMR
(CDCl3 376 MHz)
115 110 105 100 95 90 85 80 75 70 65 60 55 50 45 40 35 30 25 20 15 10 5 0 -5

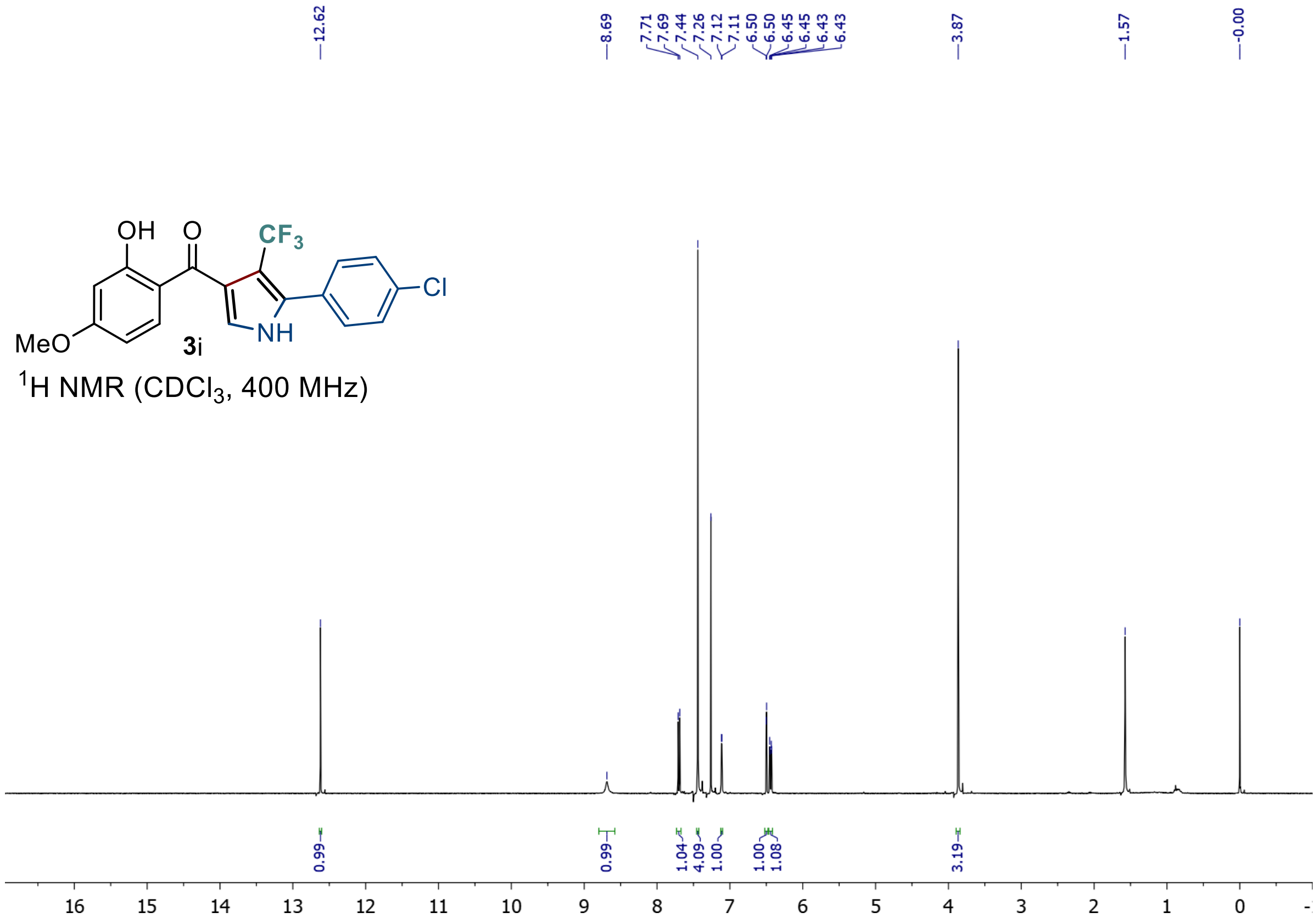
OH
O
CF3
Cl
NH
MeO
3i
1H NMR (CDCl3, 400 MHz)

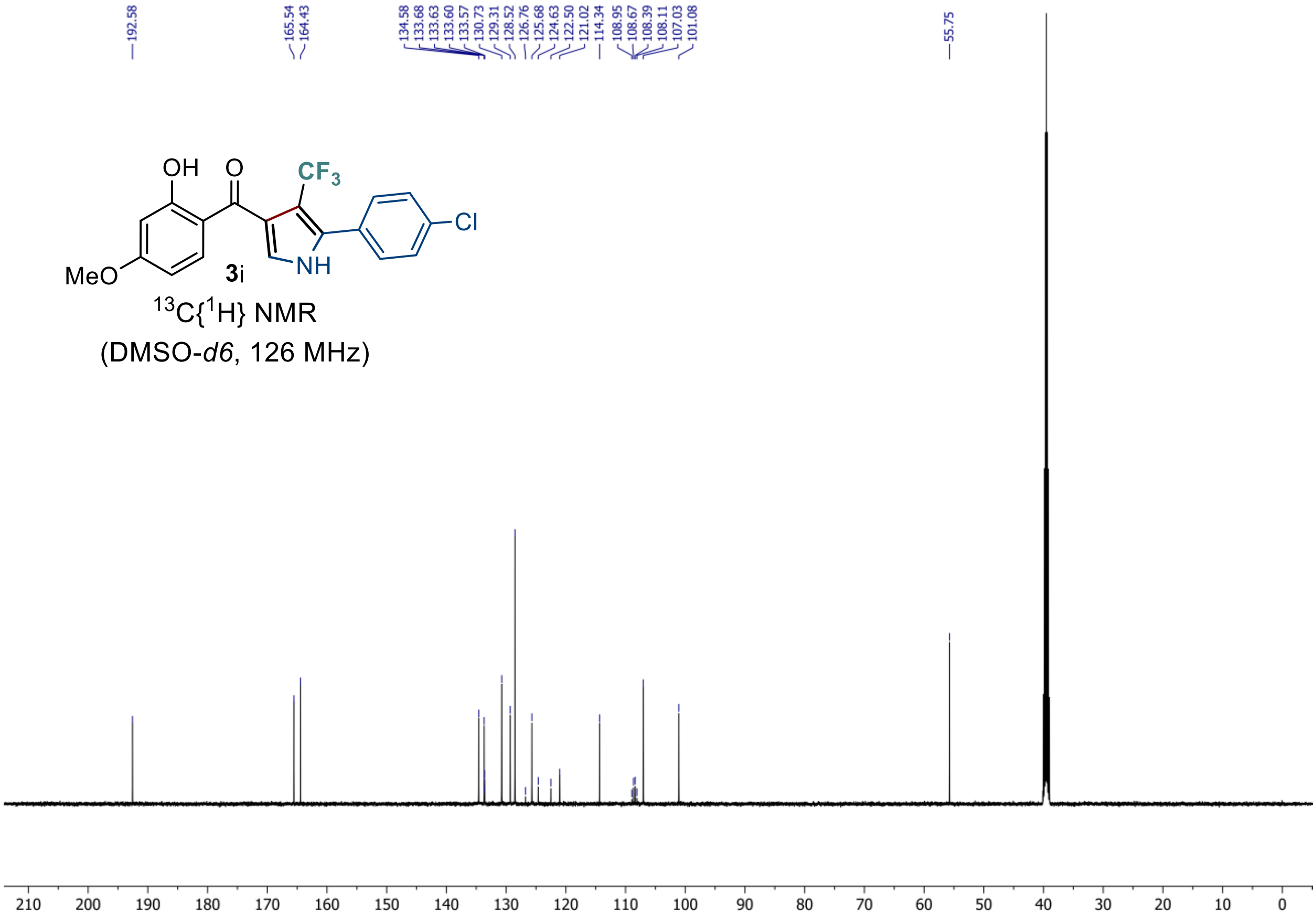
OH
O
CF3
Cl
NH
MeO
3i
13C{1H} NMR
(DMSO-d6, 126 MHz)

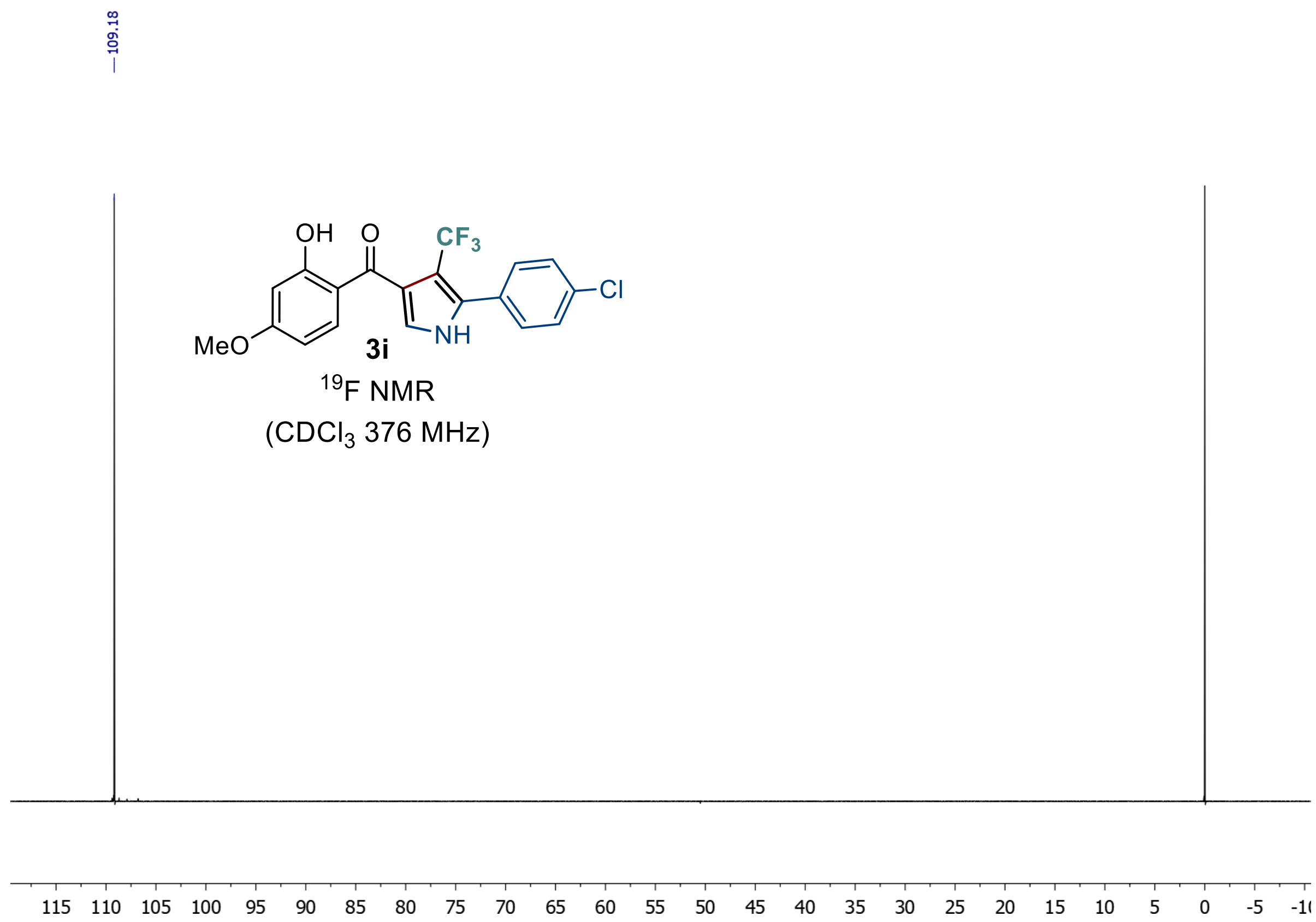

109.18
OH
O
CF3
Cl
MeO
NH
3i
19F NMR
(CDCl3 376 MHz)
115 110 105 100 95 90 85 80 75 70 65 60 55 50 45 40 35 30 25 20 15 10 5 0 -5

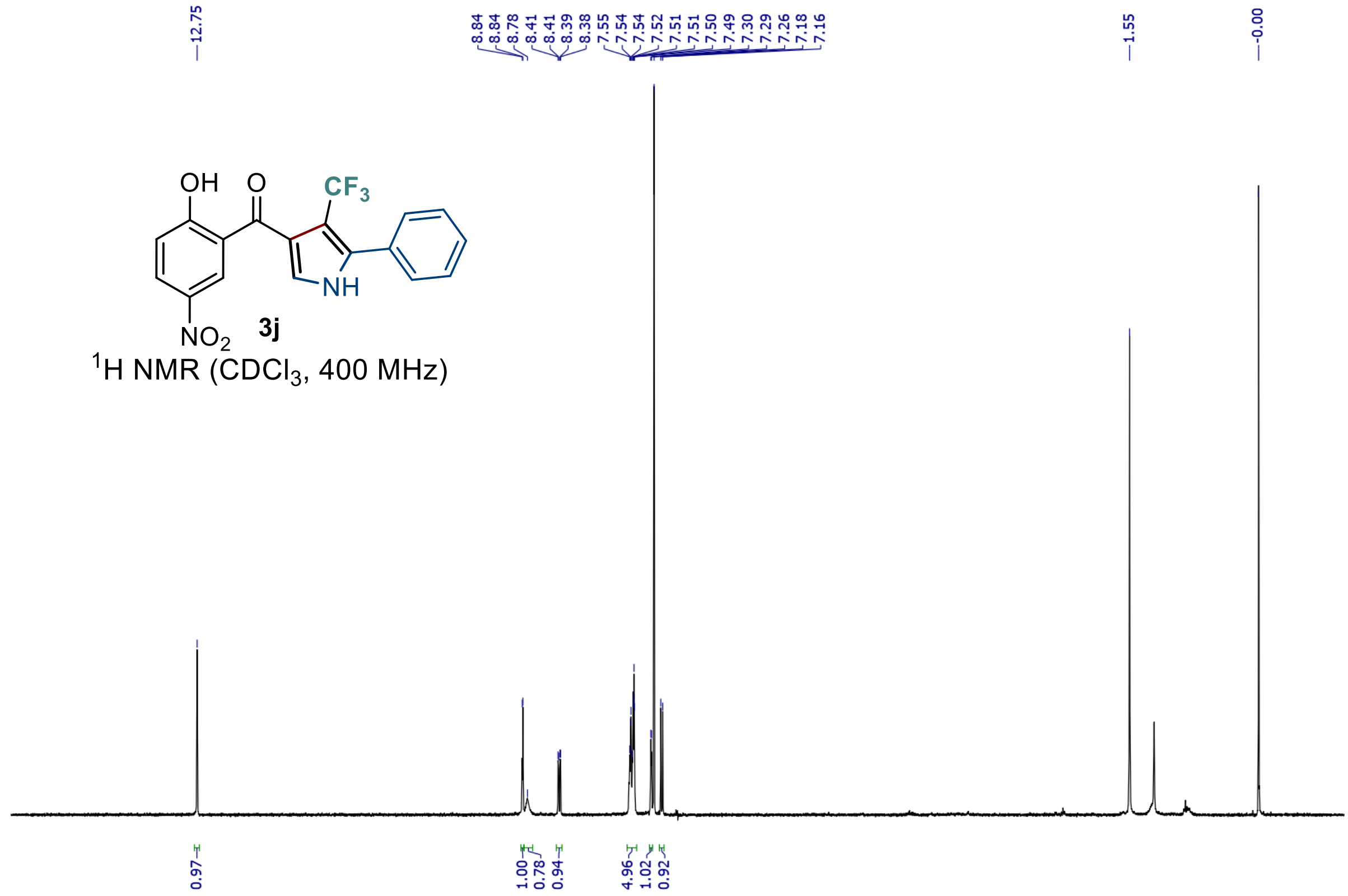
12.75
8.84
8.84
8.78
8.41
8.41
8.39
8.38
7.55
7.54
7.54
7.52
7.51
7.51
7.50
7.49
7.30
7.29
7.26
7.18
7.16
1.55
-0.00
OH
O
CF3
NH
NO2
3j
1H NMR (CDCl3, 400 MHz)
0.97
1.00
0.78
0.94
4.96
1.02
0.92
14
13
12
11
10
9
8
7
6
5
4
3
2
1
0

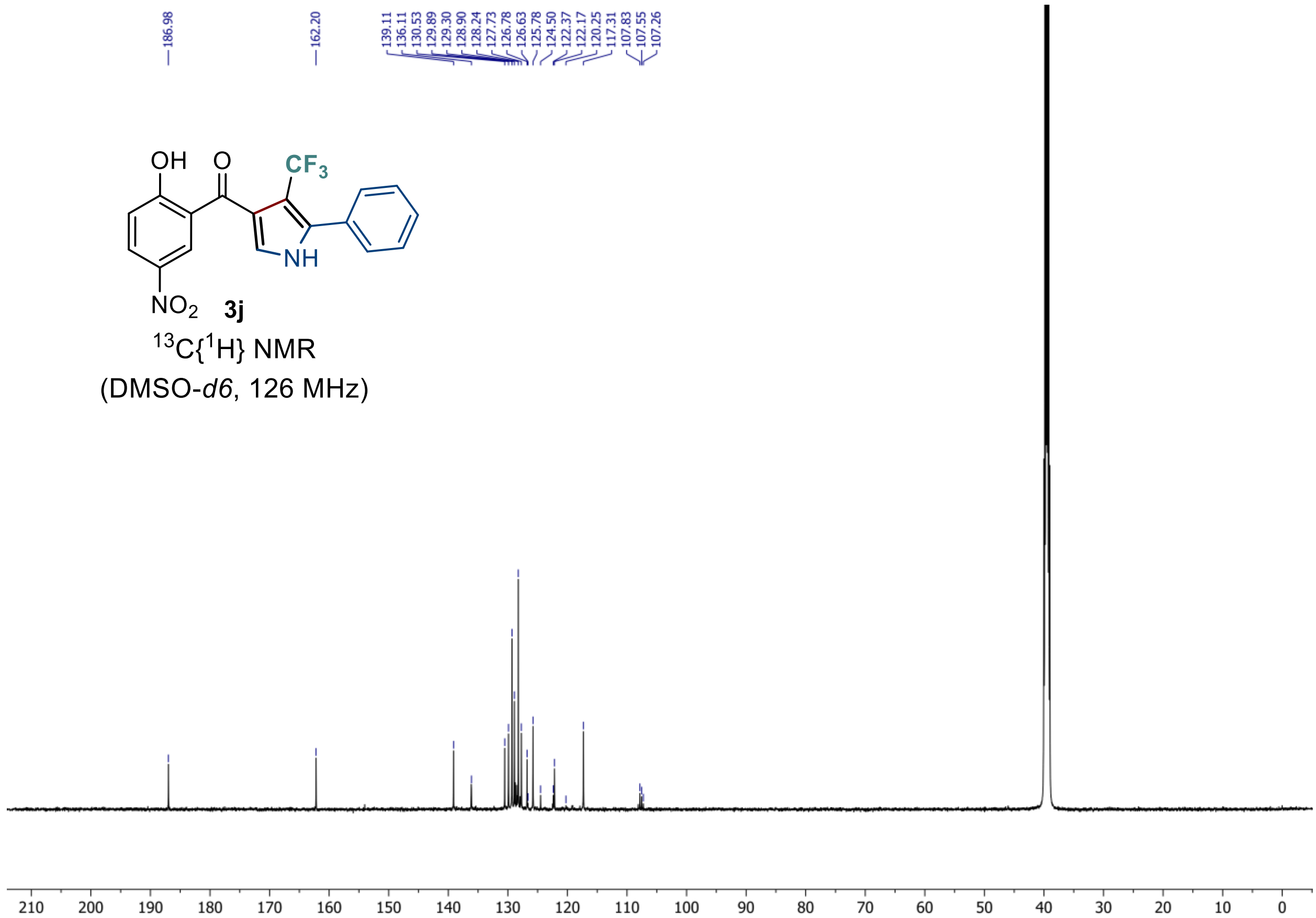
186.98
162.20
139.11
136.11
130.53
129.89
129.30
128.90
128.24
127.73
126.78
126.63
125.78
124.50
122.37
122.17
120.25
117.31
107.83
107.55
107.26
OH
O
CF3
NH
NO2
3j
13C{1H} NMR
(DMSO-d6, 126 MHz)
210
200
190
180
170
160
150
140
130
120
110
100
90
80
70
60
50
40
30
20
10
0

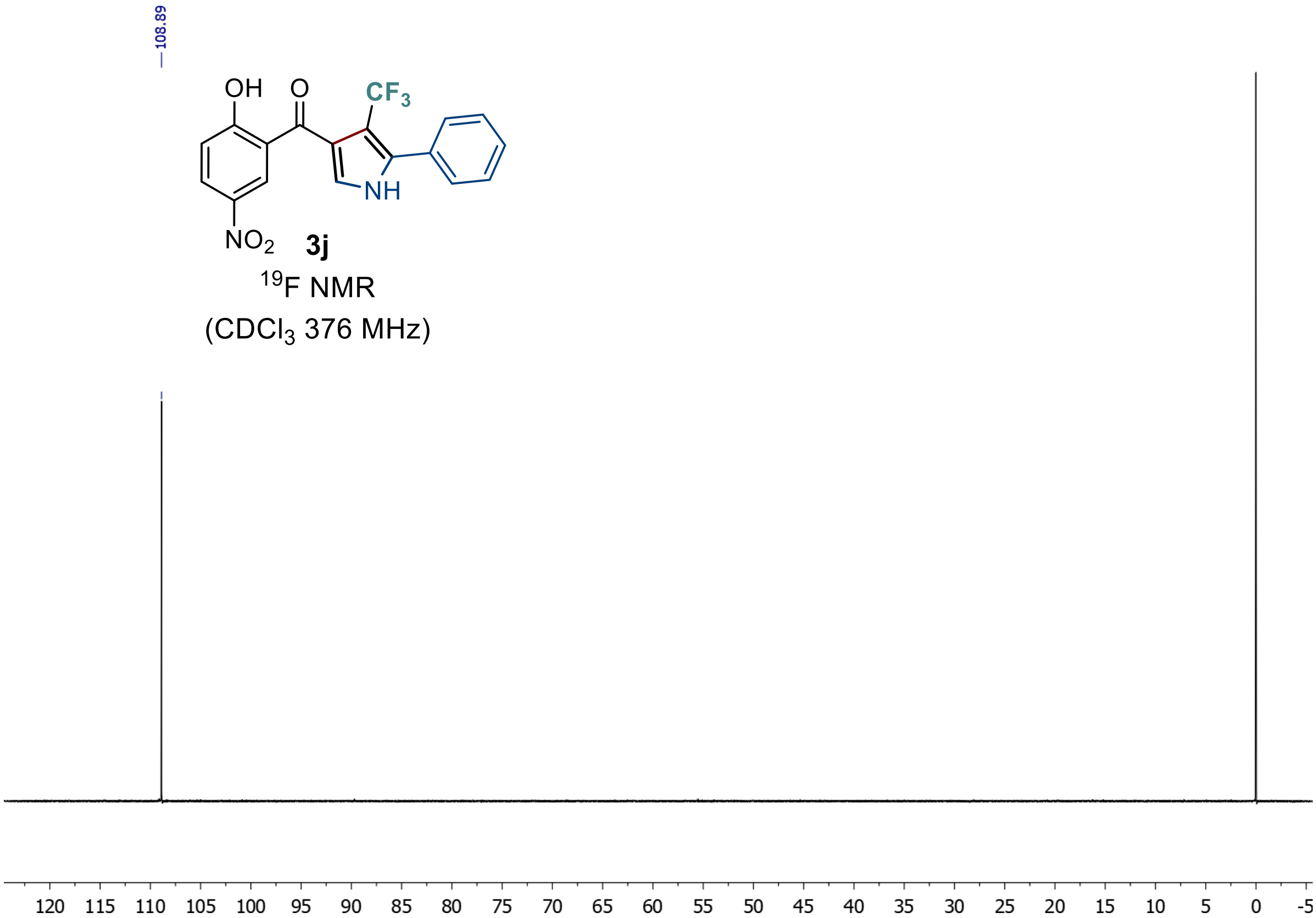

108.89
OH
O
CF3
NH
NO2
3j
19F NMR
(CDCl3 376 MHz)
120 115 110 105 100 95 90 85 80 75 70 65 60 55 50 45 40 35 30 25 20 15 10 5 0 -5

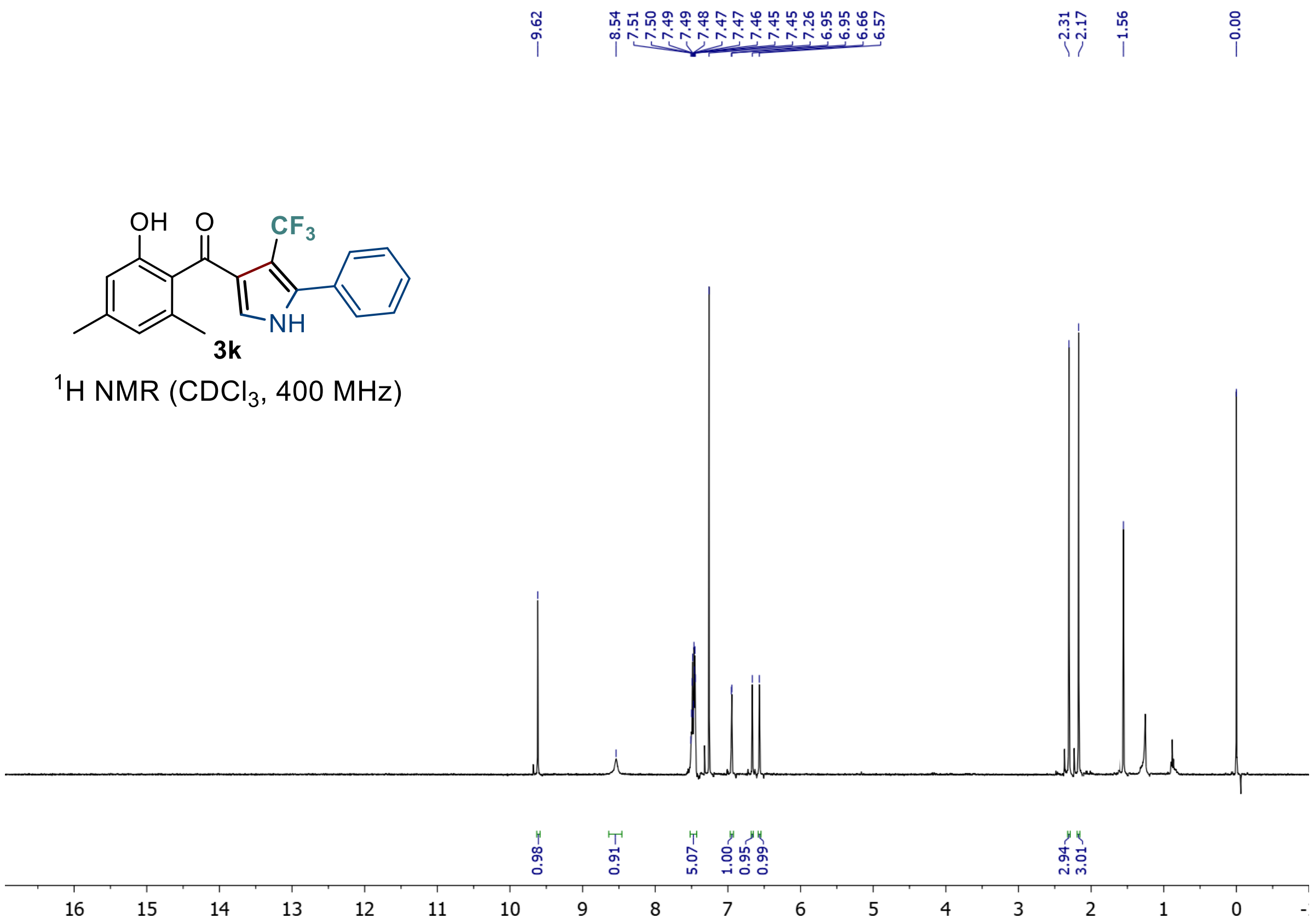

OH
O
CF3
NH
3k
1H NMR (CDCl3, 400 MHz)

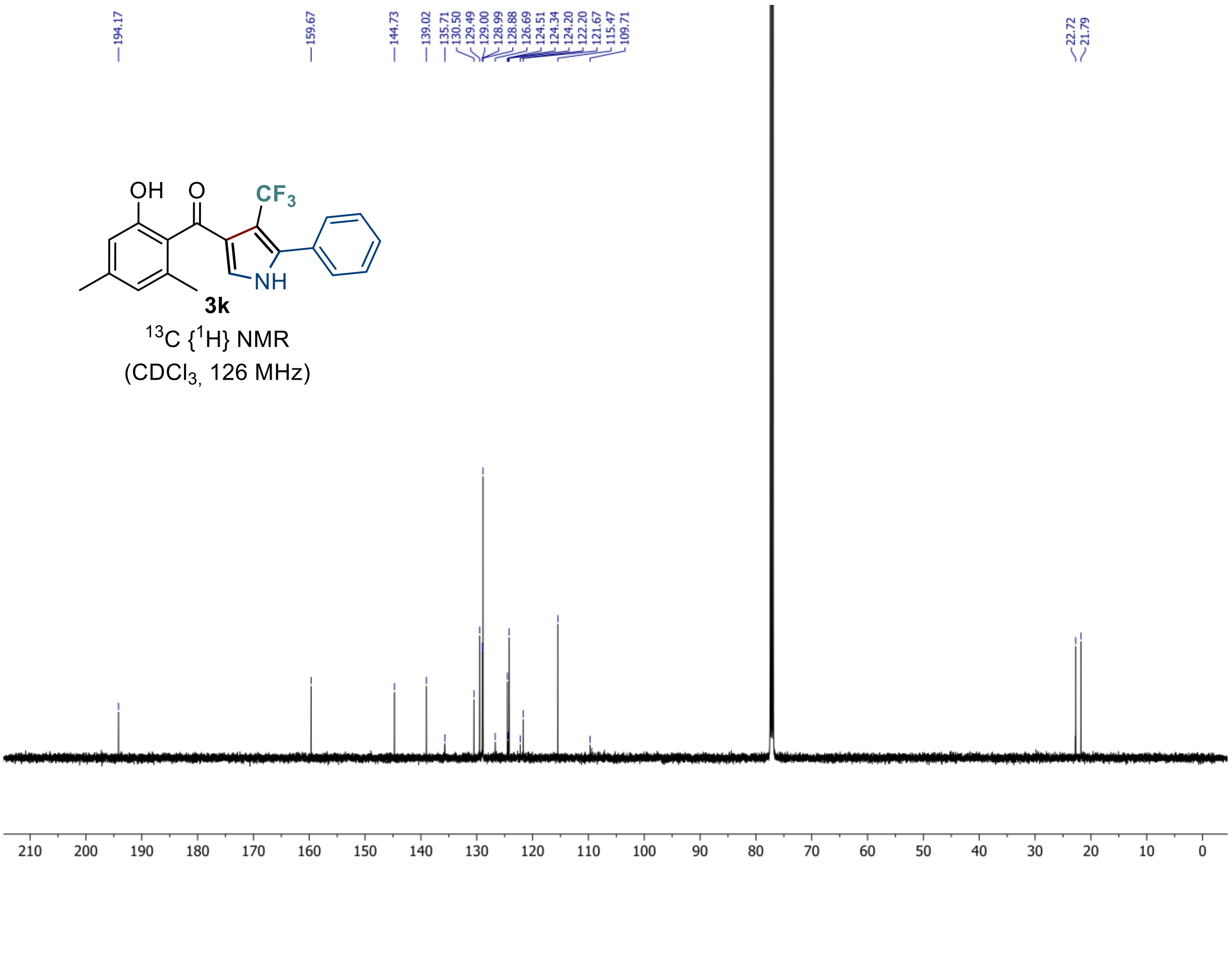

OH
O
CF3
NH
3k
13C {1H} NMR
(CDCl3, 126 MHz)

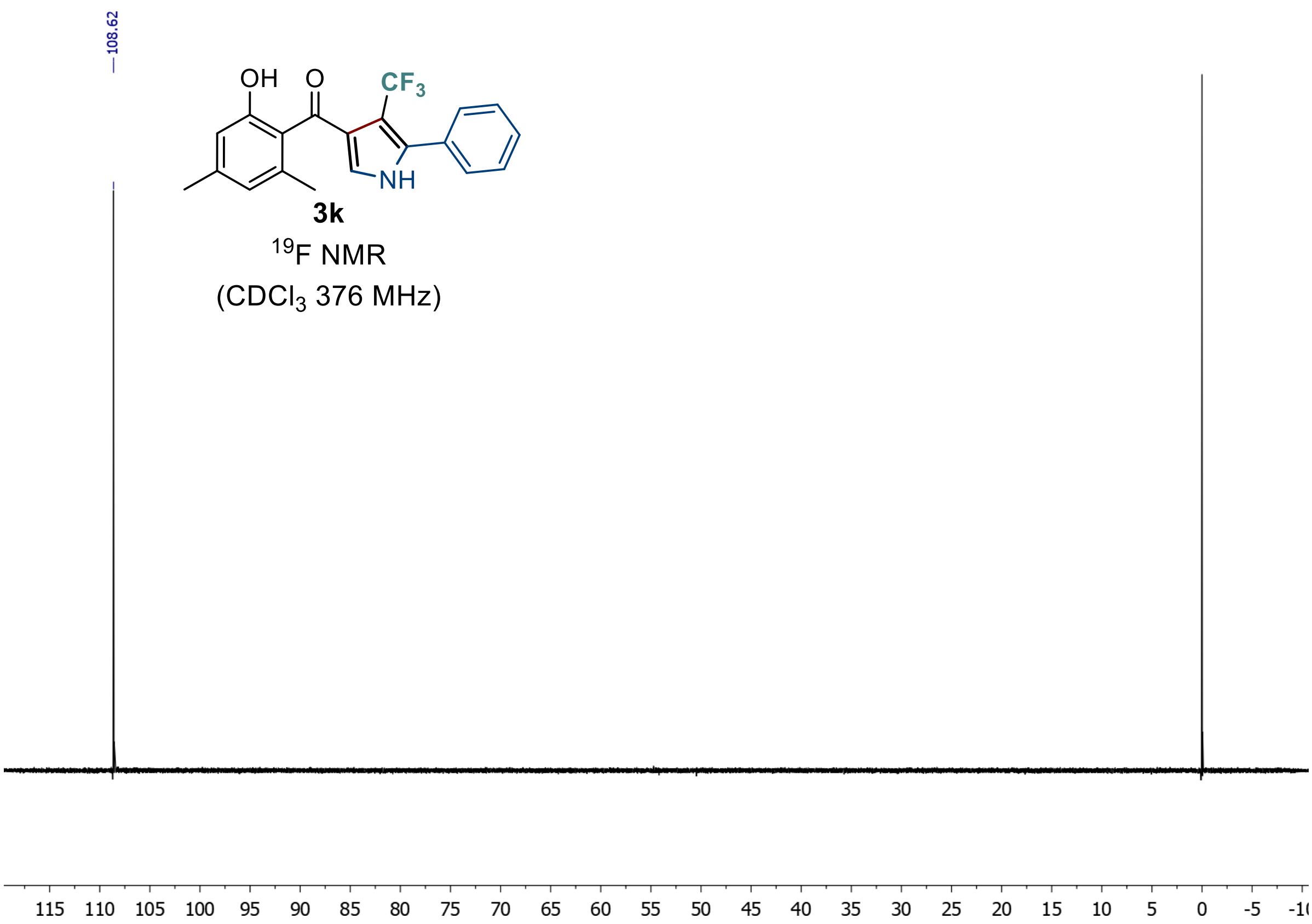

108.62
OH
O
CF3
NH
3k
19F NMR
(CDCl3 376 MHz)
115 110 105 100 95 90 85 80 75 70 65 60 55 50 45 40 35 30 25 20 15 10 5 0 -5 -1

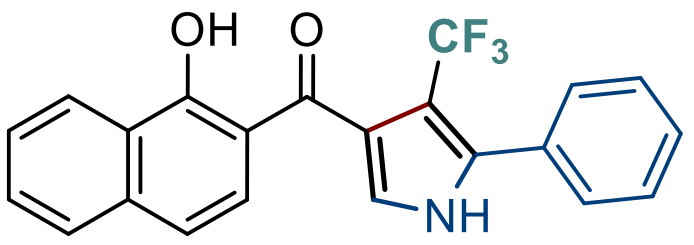

3l

^{1}H NMR ($CDCl_3$, 400 MHz)

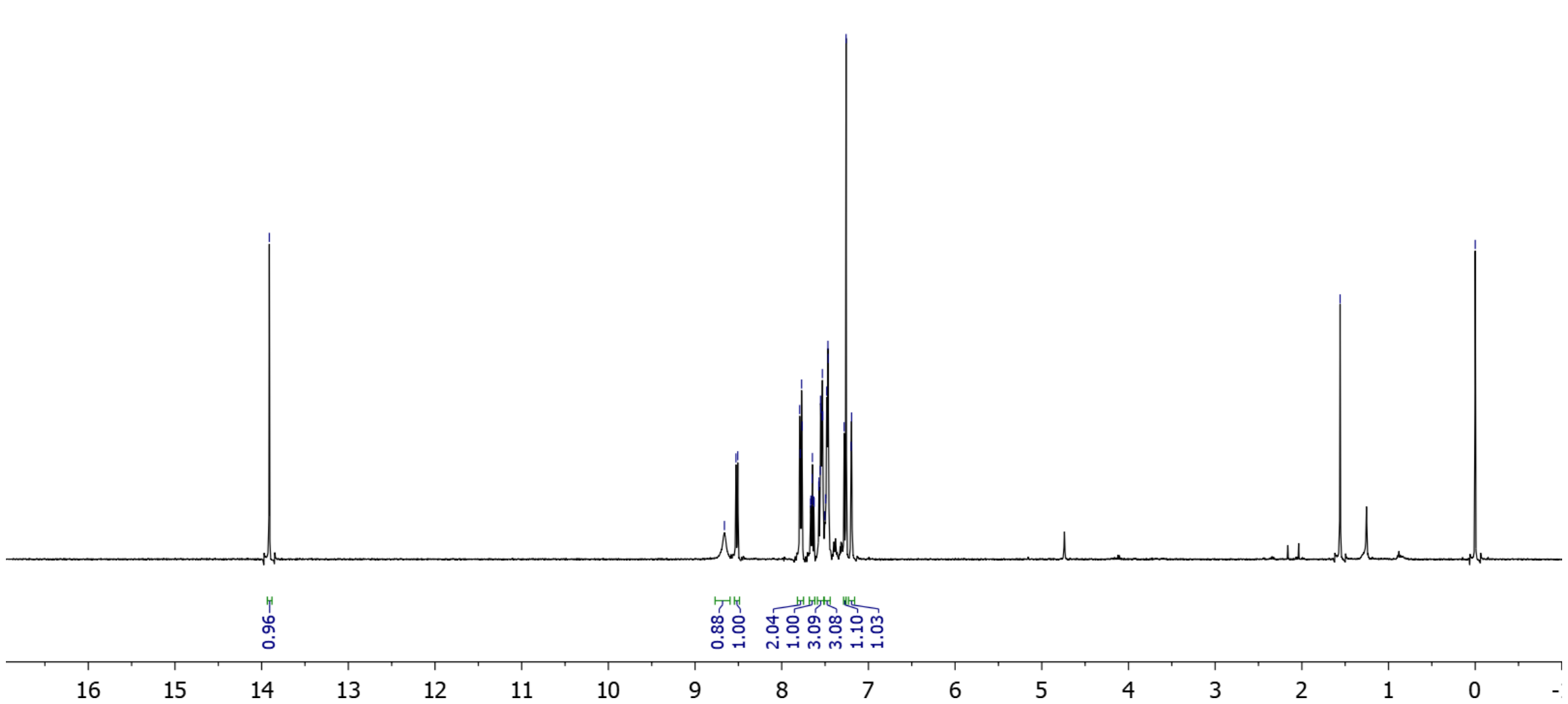

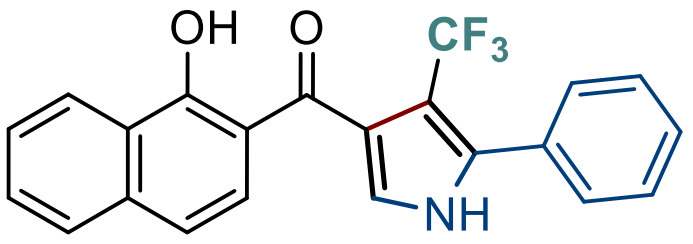

3l

$^{13}C\{^{1}H\}$ NMR

(DMSO-*d6*, 126 MHz)

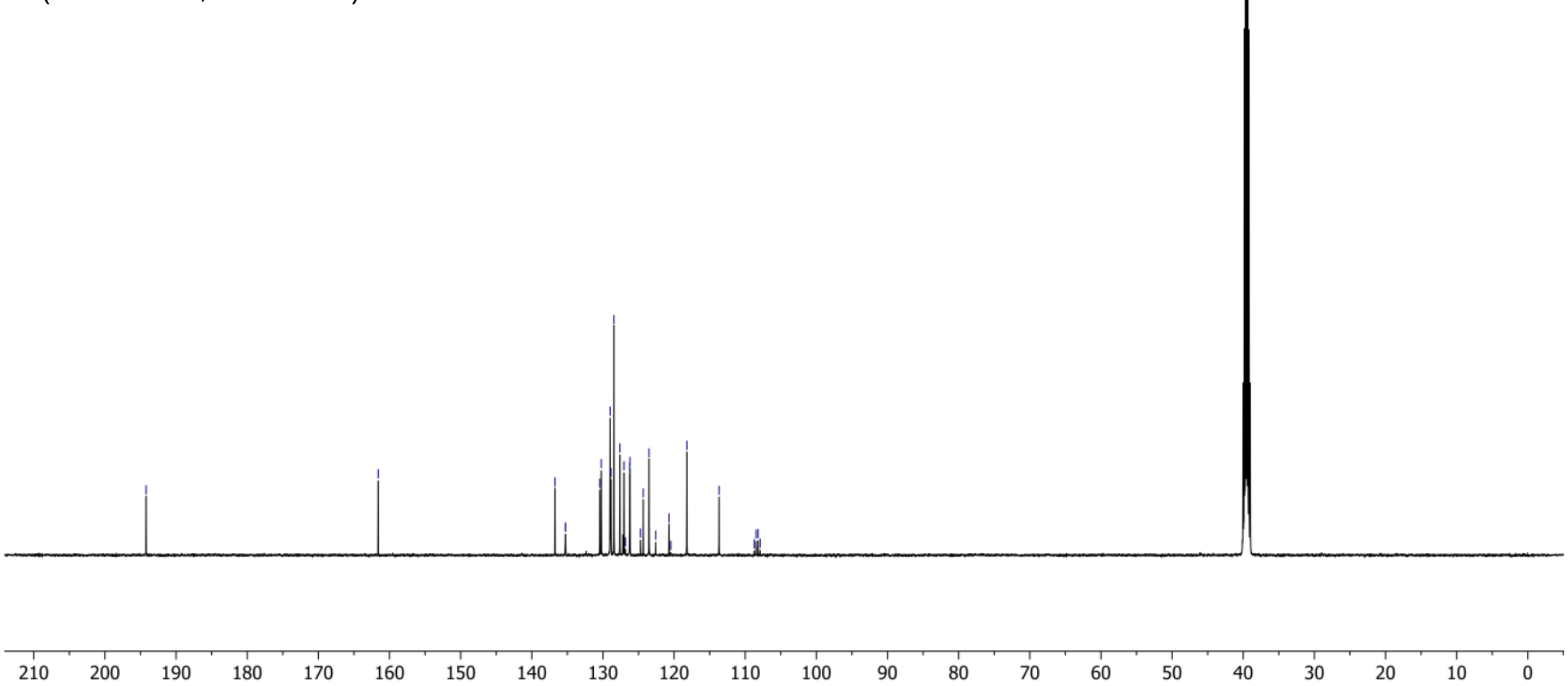

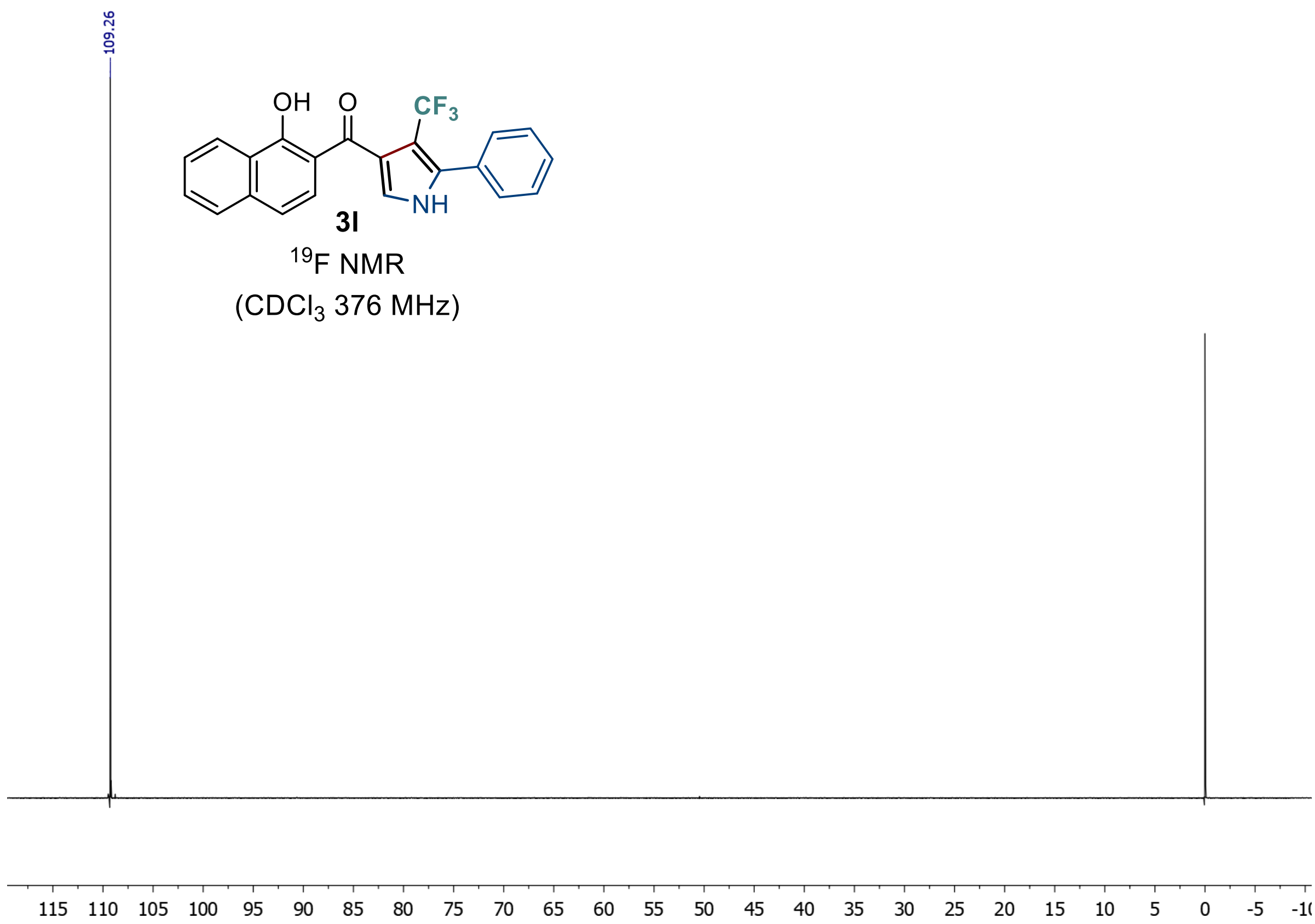

-109.26
OH
O
CF_3
NH
3l
^{19}F NMR
($CDCl_3$ 376 MHz)
115 110 105 100 95 90 85 80 75 70 65 60 55 50 45 40 35 30 25 20 15 10 5 0 -5 -10

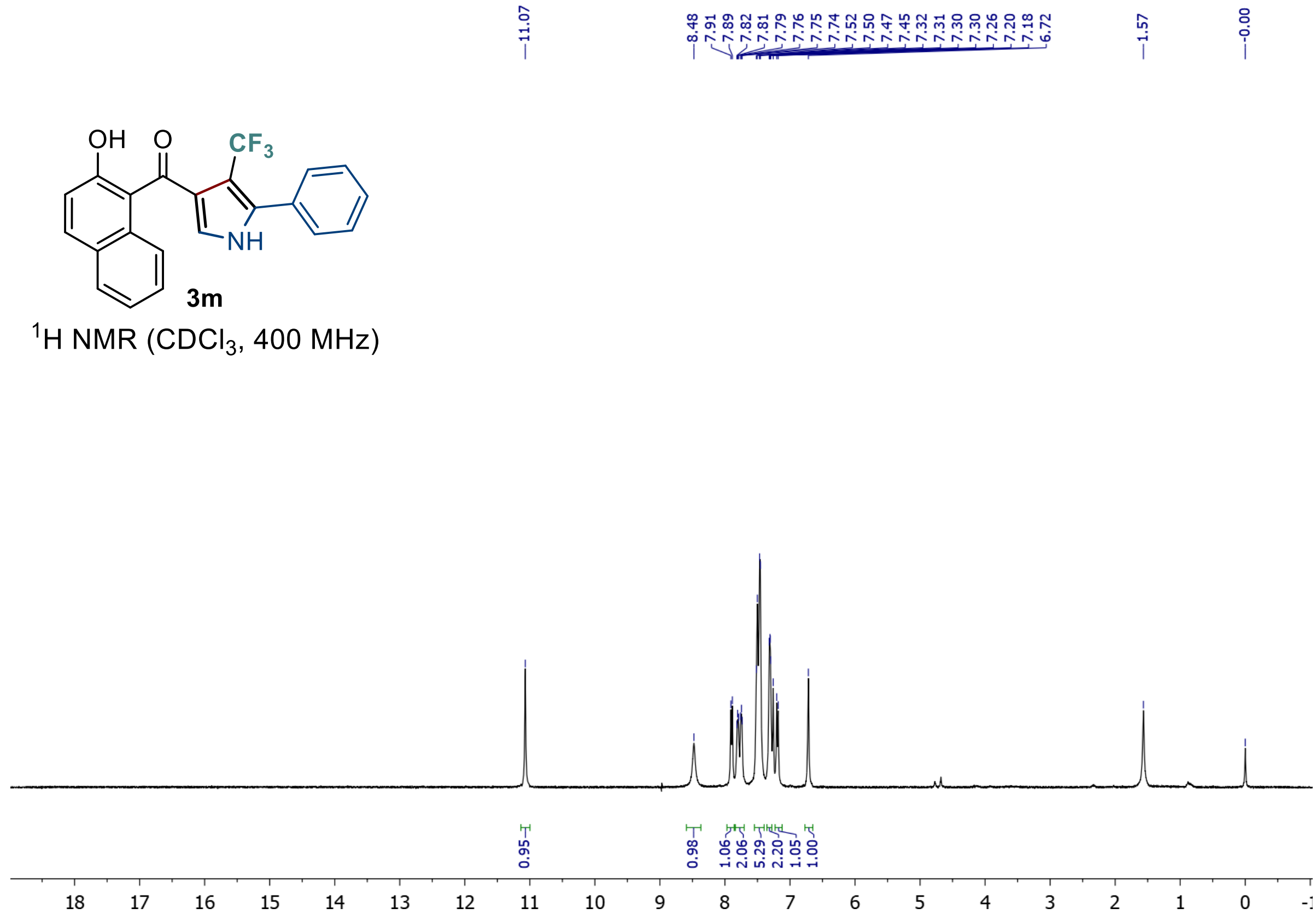

3m

^{1}H NMR (CDCl$_3$, 400 MHz)

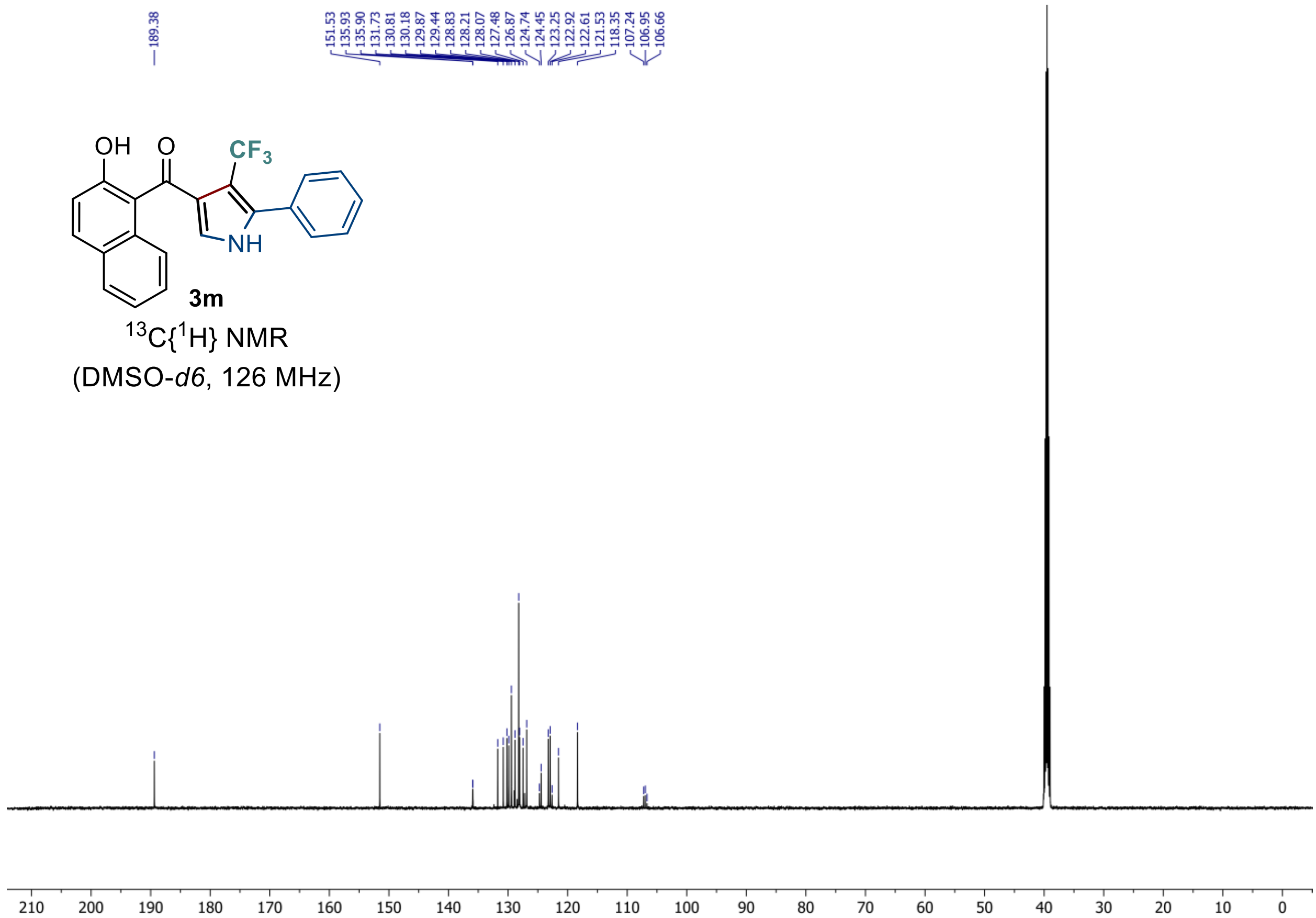

3m

^{13}C{^{1}H} NMR

(DMSO-*d6*, 126 MHz)

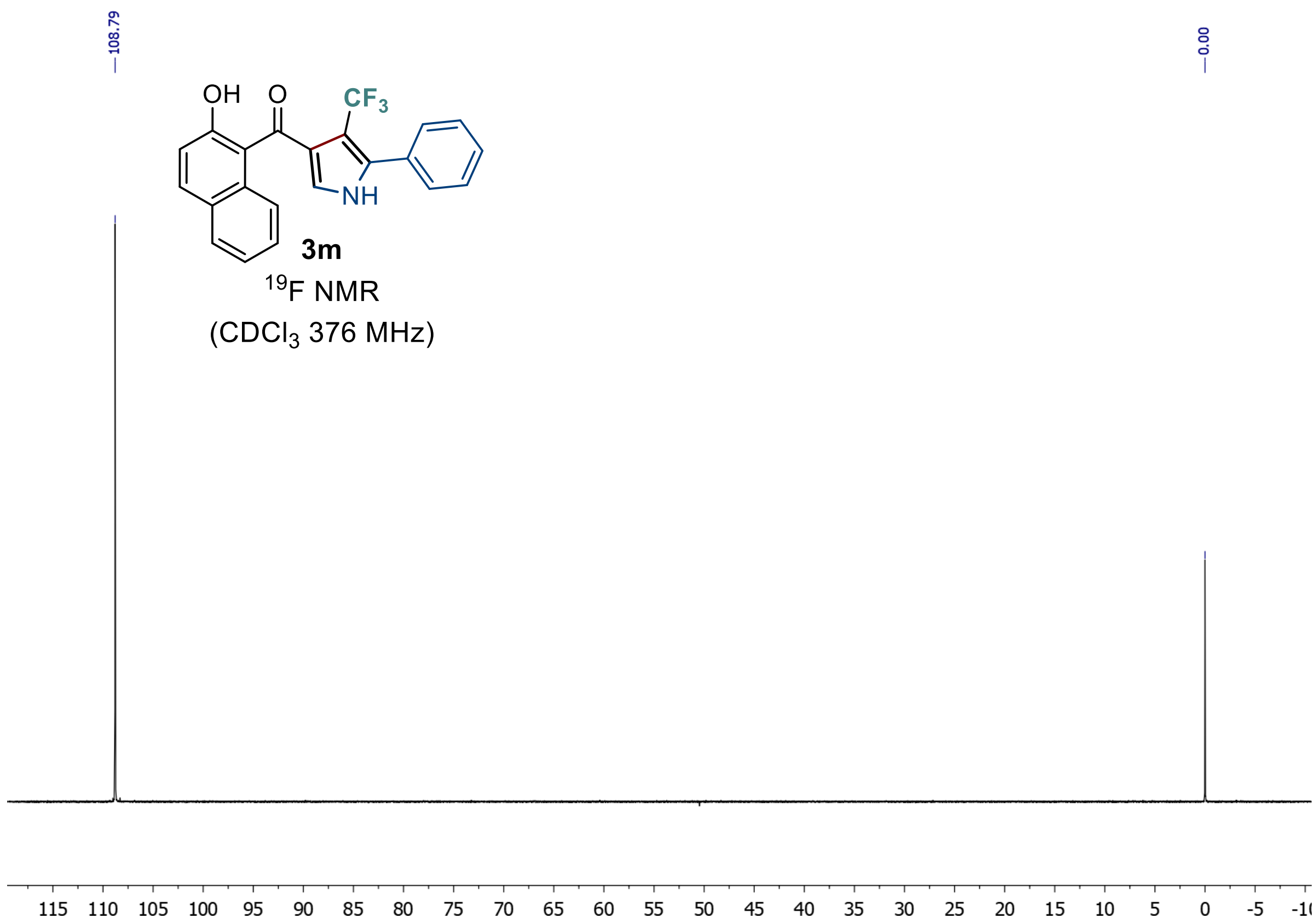

108.79
0.00
OH
O
CF3
NH
3m
19F NMR
(CDCl3 376 MHz)
115 110 105 100 95 90 85 80 75 70 65 60 55 50 45 40 35 30 25 20 15 10 5 0 -5

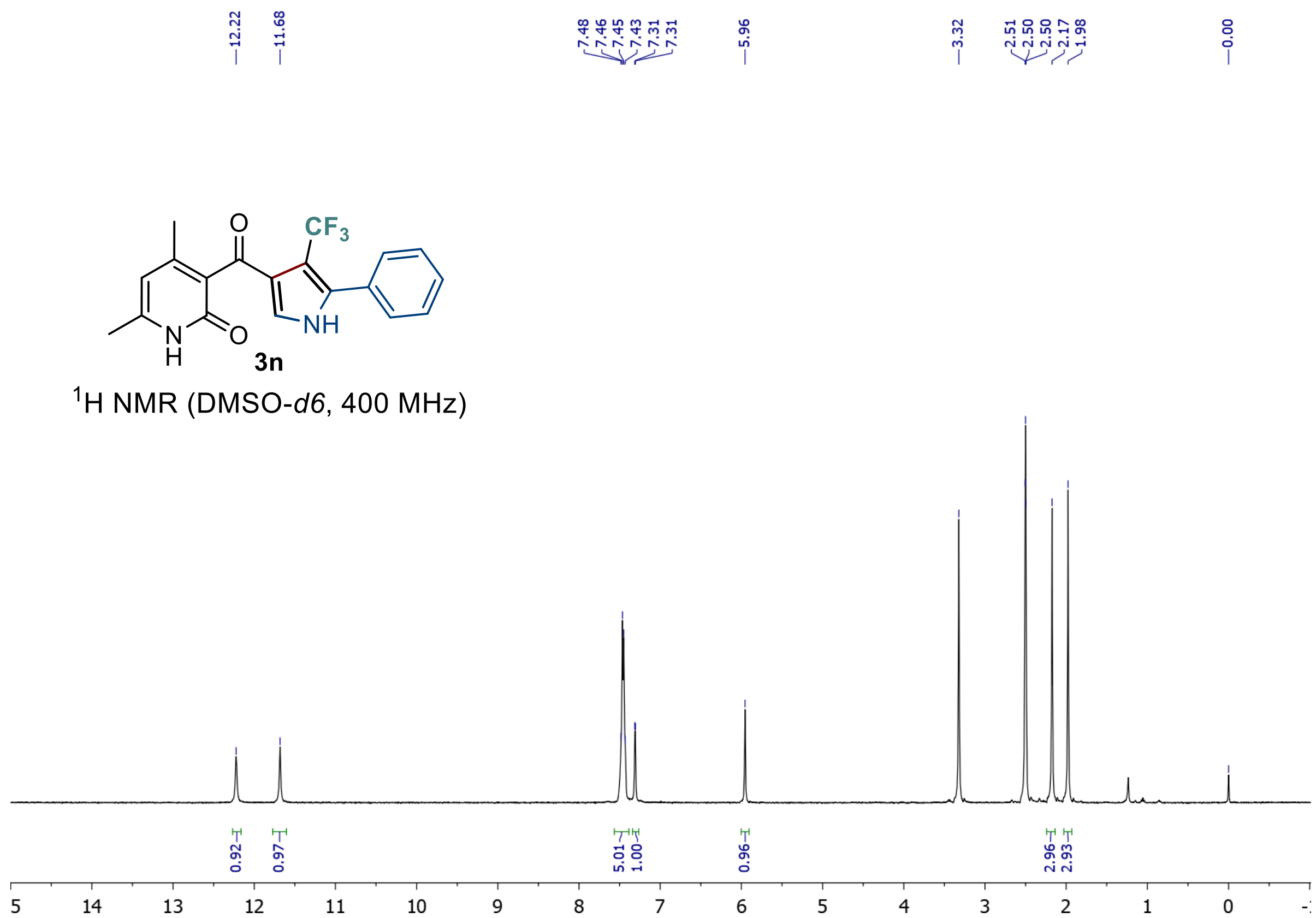
12.22
11.68
7.48
7.46
7.45
7.43
7.31
7.31
5.96
3.32
2.51
2.50
2.50
2.17
1.98
0.00
CF3
O
O
N
H
NH
3n
1H NMR (DMSO-d6, 400 MHz)
0.92
0.97
5.01
1.00
0.96
2.96
2.93
14
13
12
11
10
9
8
7
6
5
4
3
2
1
0

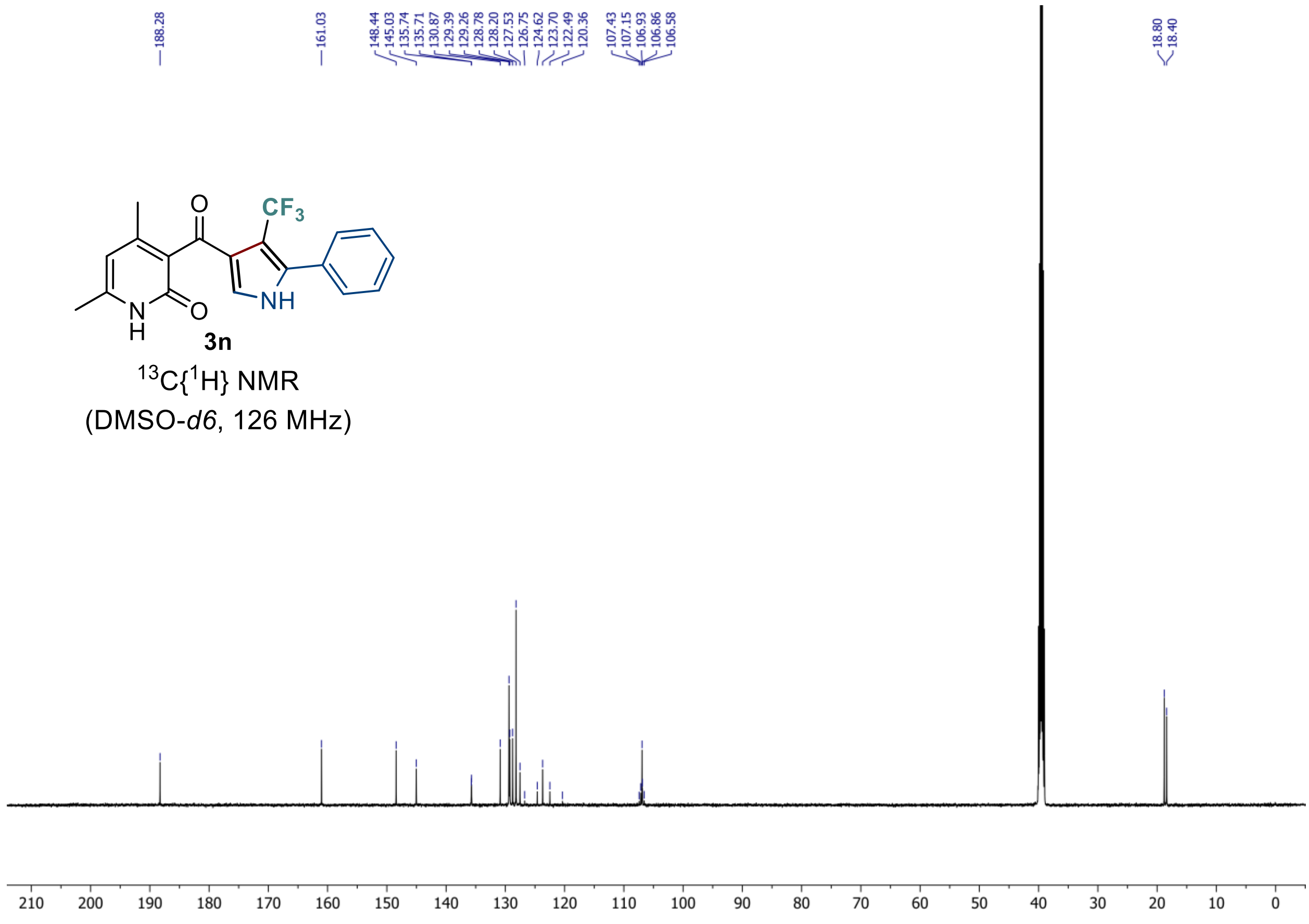
188.28
161.03
148.44
145.03
135.74
135.71
130.87
129.39
129.26
128.78
128.20
127.53
126.75
124.62
123.70
122.49
120.36
107.43
107.15
106.93
106.86
106.58
18.80
18.40
CF3
O
O
N
H
NH
3n
13C{1H} NMR
(DMSO-d6, 126 MHz)
210
200
190
180
170
160
150
140
130
120
110
100
90
80
70
60
50
40
30
20
10
0

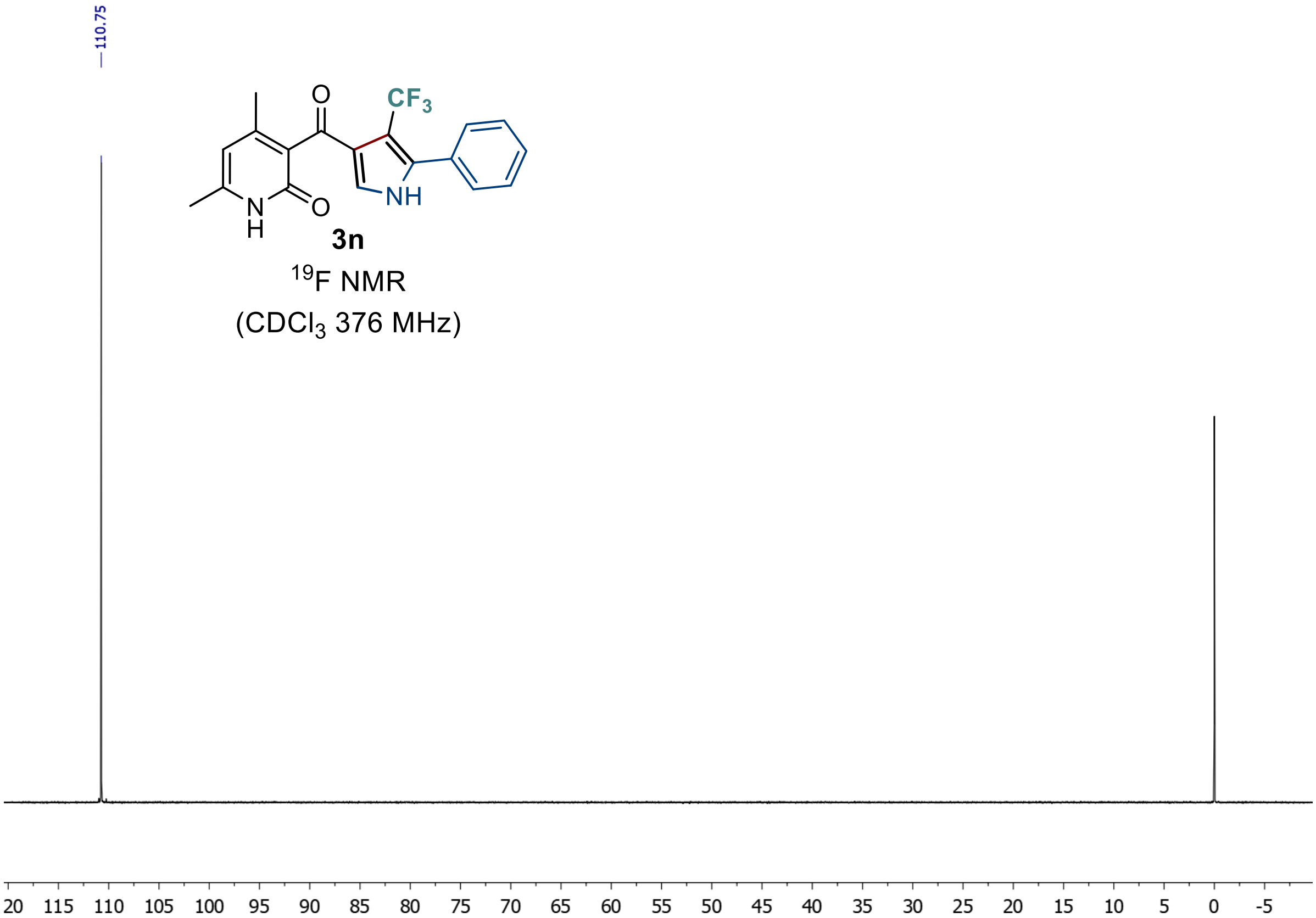
—110.75
CF3
O
NH
N
H
O
3n
19F NMR
(CDCl3 376 MHz)
20 115 110 105 100 95 90 85 80 75 70 65 60 55 50 45 40 35 30 25 20 15 10 5 0 -5

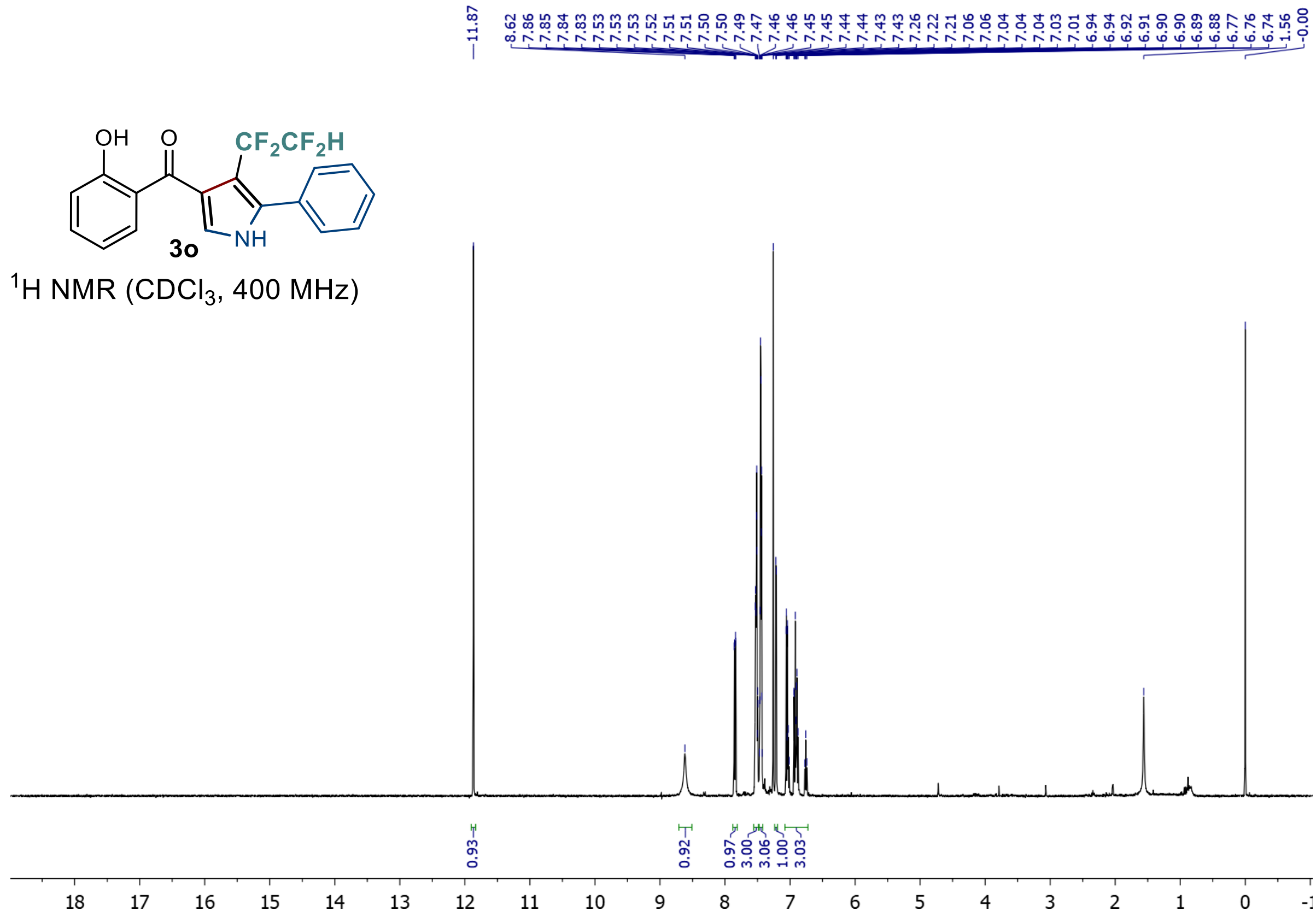

11.87
8.62
7.86
7.85
7.84
7.83
7.53
7.53
7.53
7.52
7.51
7.51
7.50
7.50
7.49
7.47
7.46
7.46
7.45
7.45
7.44
7.44
7.43
7.43
7.26
7.22
7.21
7.06
7.06
7.04
7.04
7.04
7.03
7.01
6.94
6.94
6.92
6.91
6.90
6.90
6.89
6.88
6.77
6.76
6.74
1.56
-0.00
OH
O
CF2CF2H
NH
3o
¹H NMR (CDCl3, 400 MHz)
0.93
0.92
0.97
3.00
3.06
1.00
3.03

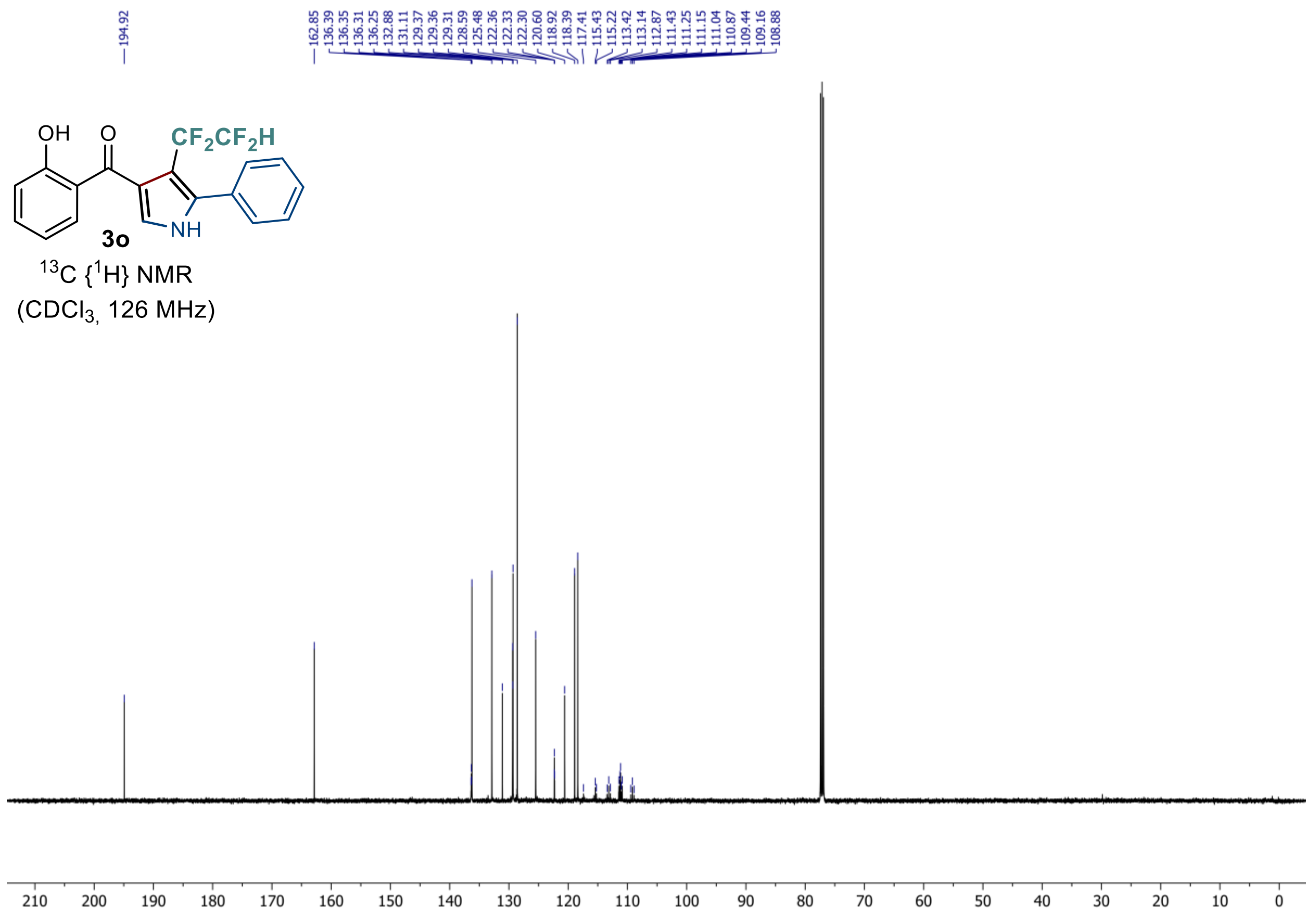

194.92
162.85
136.39
136.35
136.31
136.25
132.88
131.11
129.37
129.36
129.31
128.59
125.48
122.36
122.33
122.30
120.60
118.92
118.39
117.41
115.43
115.22
113.42
113.14
112.87
111.43
111.25
111.15
111.04
110.87
109.44
109.16
108.88
OH
O
CF2CF2H
NH
3o
¹³C {¹H} NMR
(CDCl3, 126 MHz)

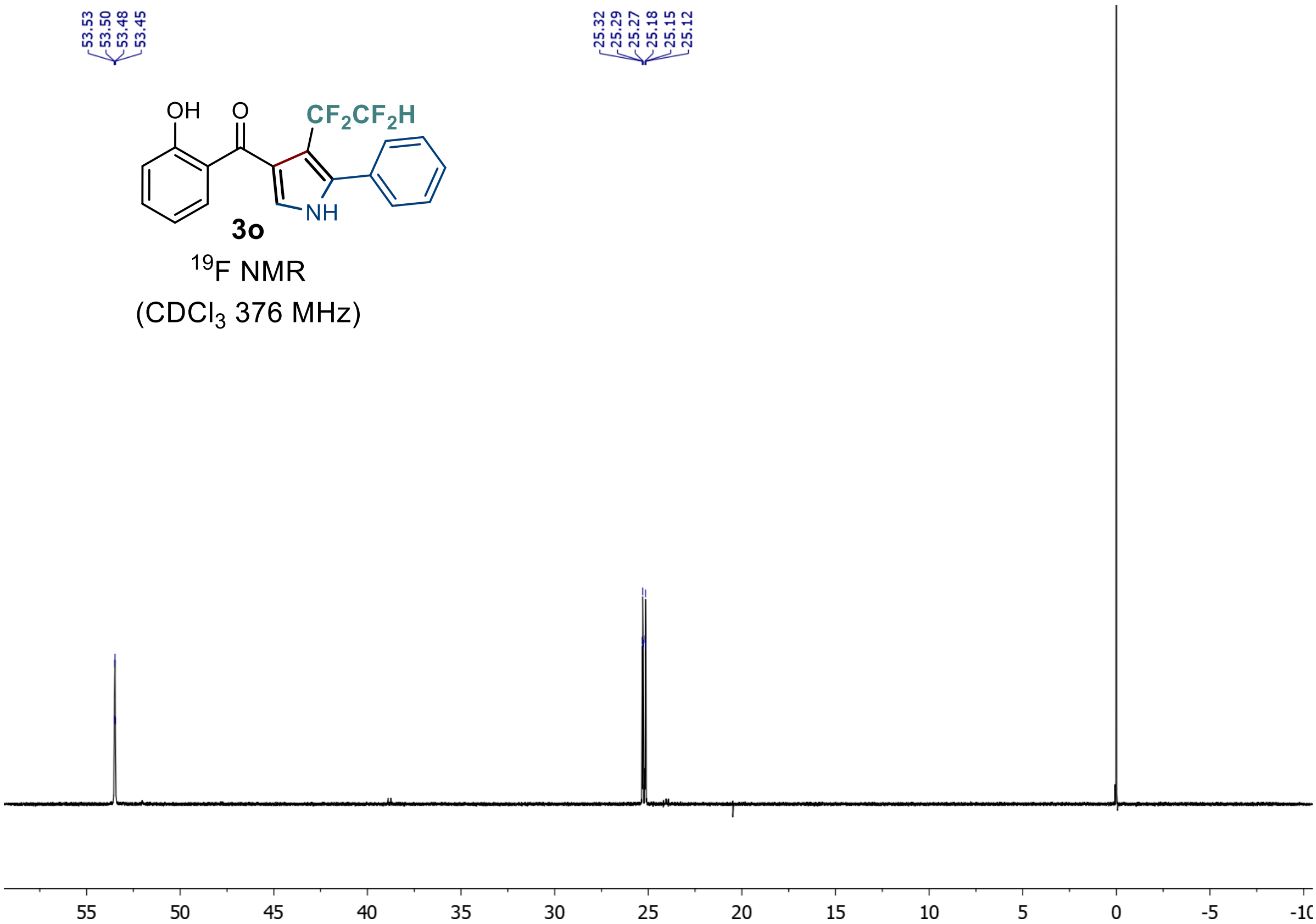
53.53
53.50
53.48
53.45
25.32
25.29
25.27
25.18
25.15
25.12
OH
O
CF2CF2H
NH
3o
19F NMR
(CDCl3 376 MHz)
55
50
45
40
35
30
25
20
15
10
5
0
-5

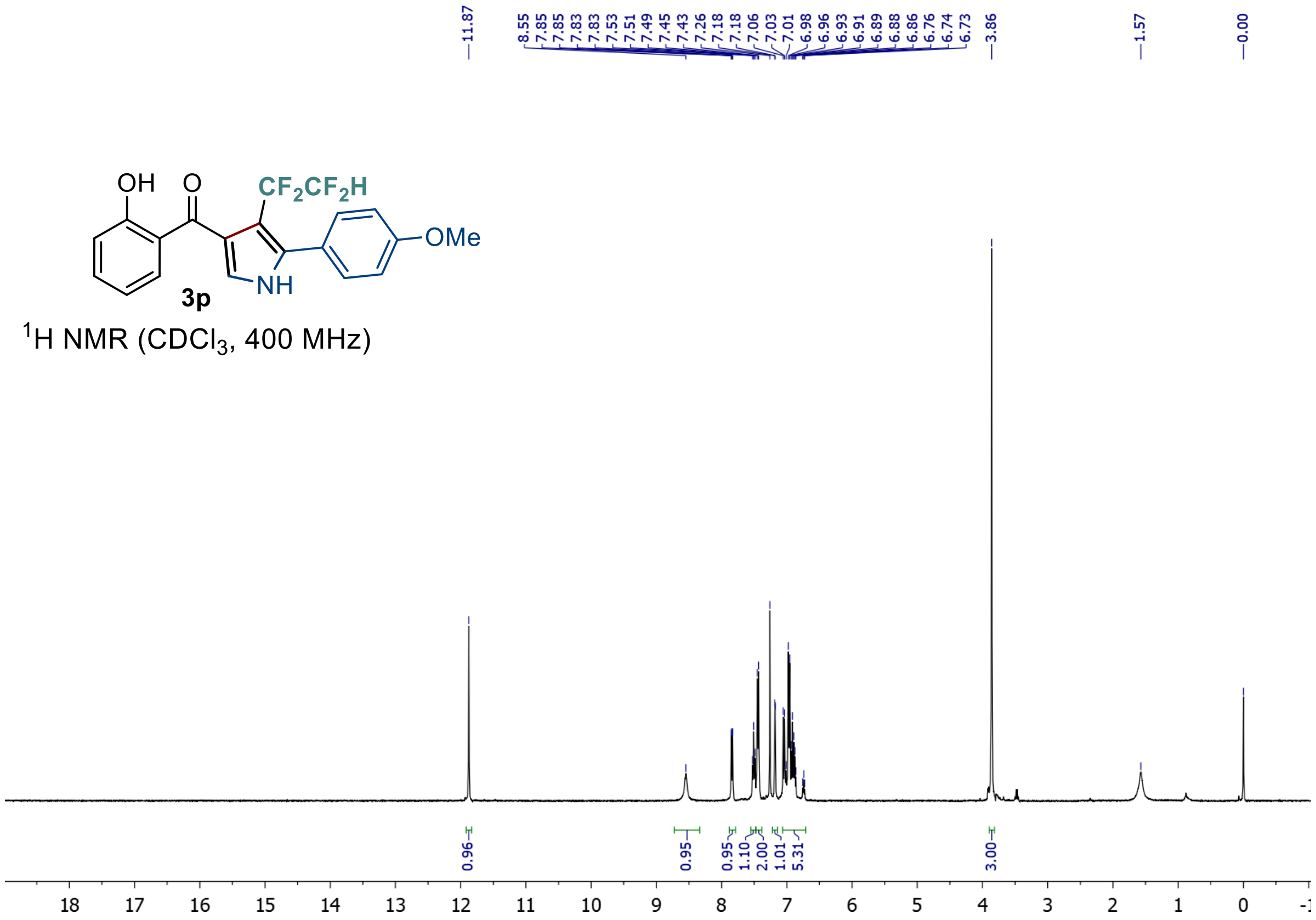

OH
O
CF2CF2H
OMe
NH
3p
1H NMR (CDCl3, 400 MHz)

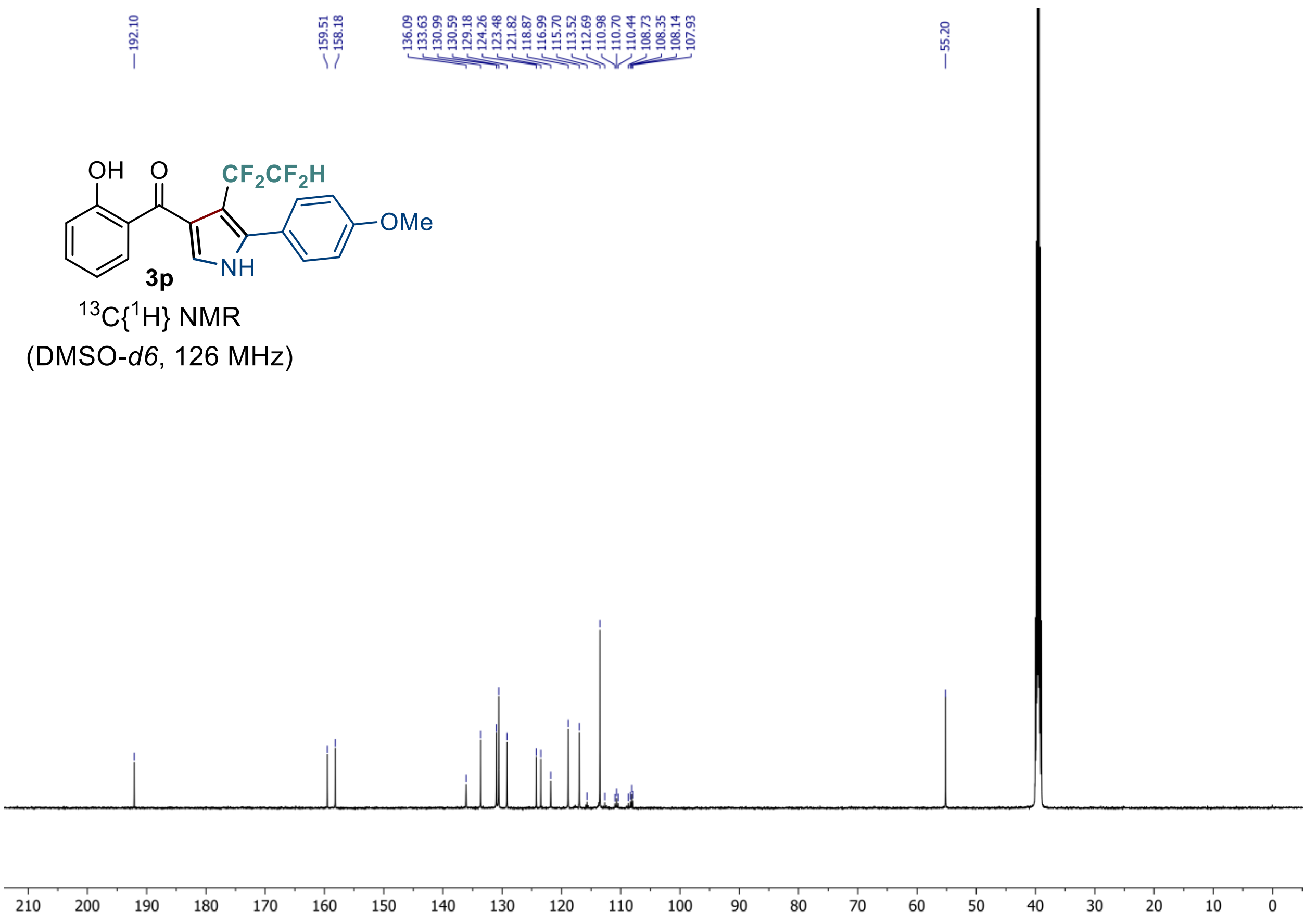

OH
O
CF2CF2H
OMe
NH
3p
13C{1H} NMR
(DMSO-d6, 126 MHz)

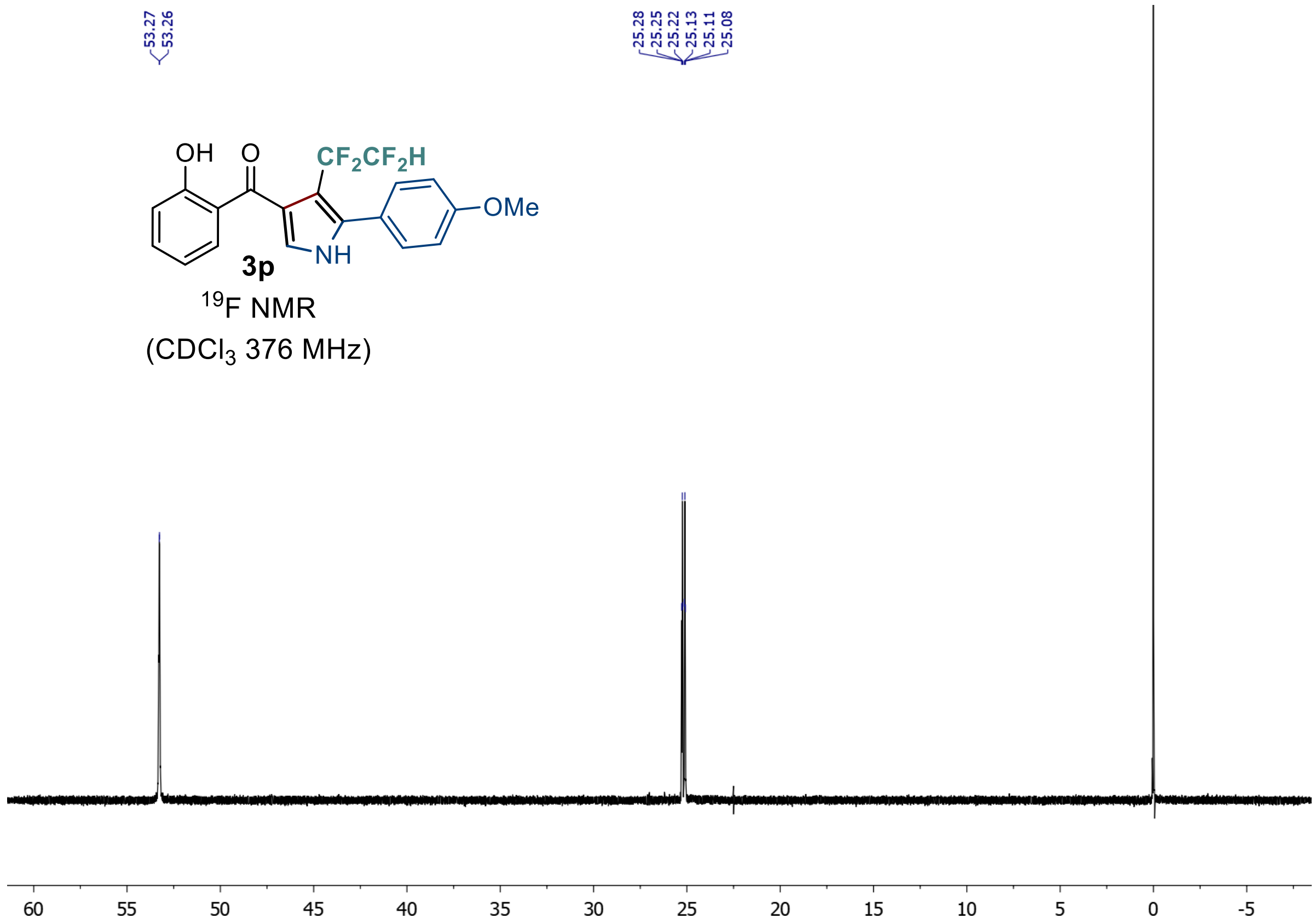
53.27
53.26
25.28
25.25
25.22
25.13
25.11
25.08
OH
O
CF2CF2H
OMe
NH
3p
19F NMR
(CDCl3 376 MHz)
60 55 50 45 40 35 30 25 20 15 10 5 0 -5

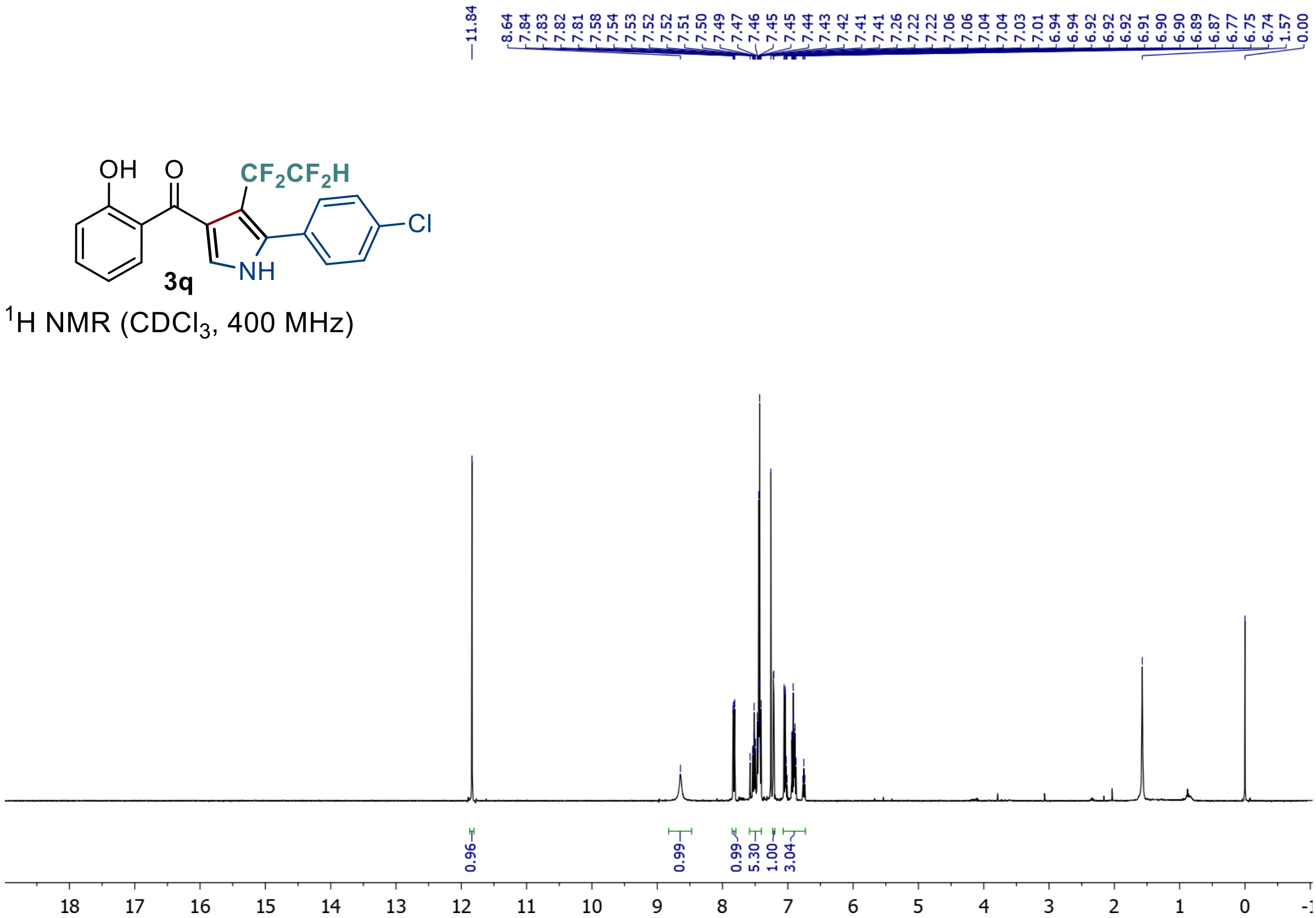
11.84
8.64 7.84 7.83 7.82 7.81 7.58 7.54 7.53 7.52 7.52 7.51 7.50 7.49 7.47 7.46 7.45 7.45 7.44 7.43 7.42 7.41 7.41 7.26 7.22 7.22 7.06 7.06 7.04 7.04 7.03 7.01 6.94 6.94 6.92 6.92 6.92 6.91 6.90 6.90 6.89 6.87 6.77 6.75 6.74 1.57 0.00
OH
O
CF2CF2H
Cl
NH
3q
1H NMR (CDCl3, 400 MHz)
0.96
0.99
0.99
5.30
1.00
3.04

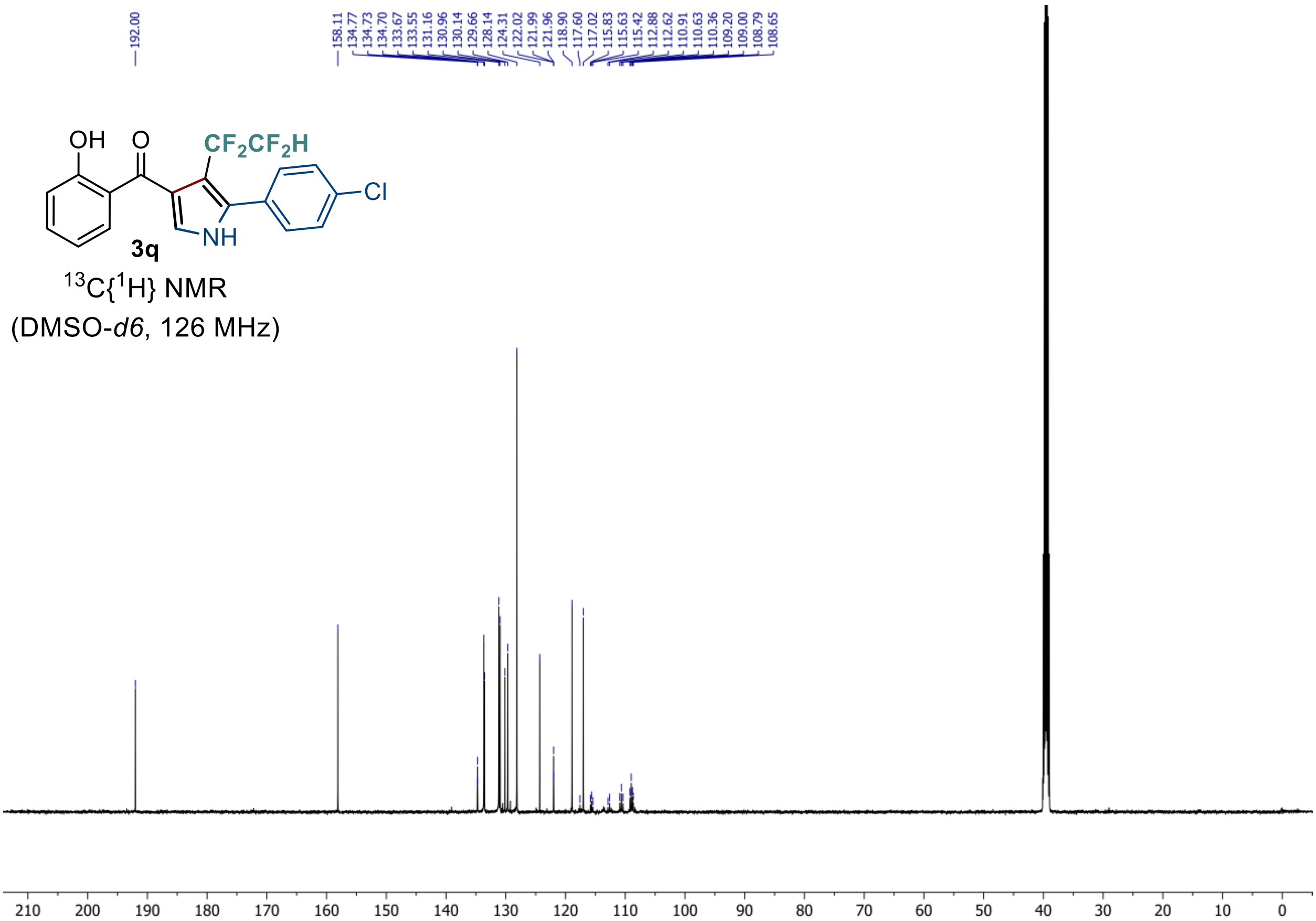
192.00
158.11 134.77 134.73 134.70 133.67 133.55 131.16 130.96 130.14 129.66 128.14 124.31 122.02 121.99 121.96 118.90 117.60 117.02 115.83 115.63 115.42 112.88 112.62 110.91 110.63 110.36 109.20 109.00 108.79 108.65
OH
O
CF2CF2H
Cl
NH
3q
13C{1H} NMR
(DMSO-d6, 126 MHz)

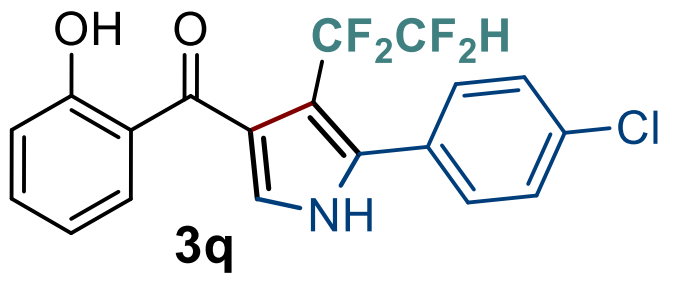

3q

^{19}F NMR

($CDCl_3$ 376 MHz)

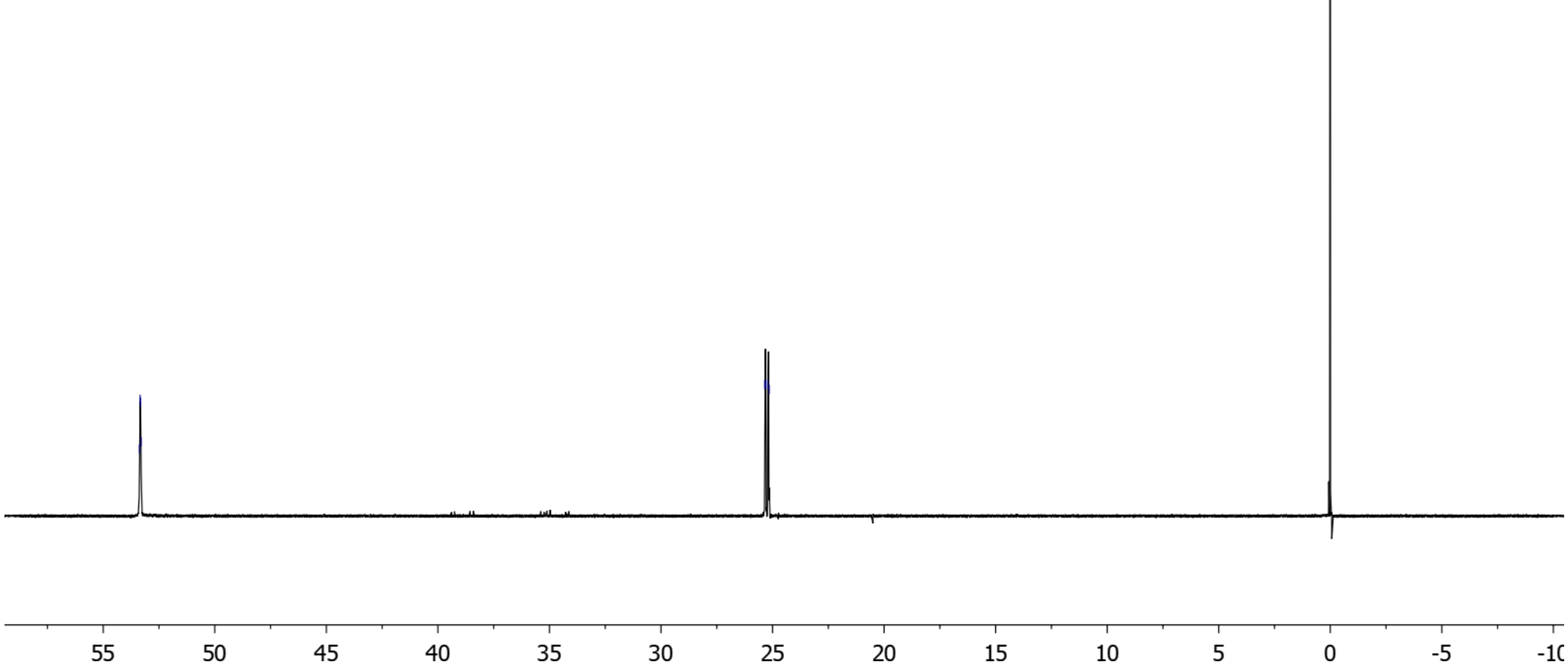

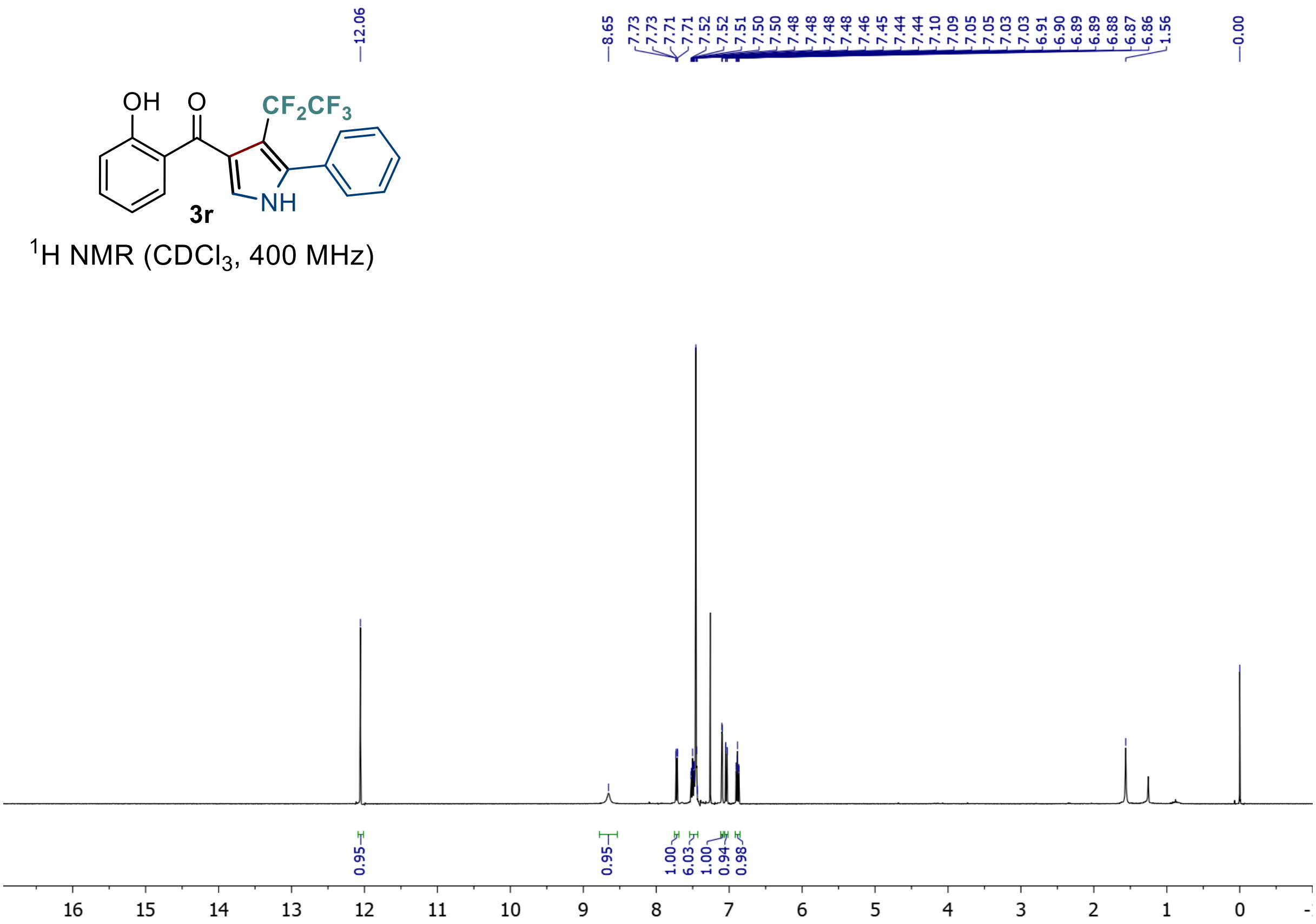

CF2CF3
OH
O
NH
3r
1H NMR (CDCl3, 400 MHz)

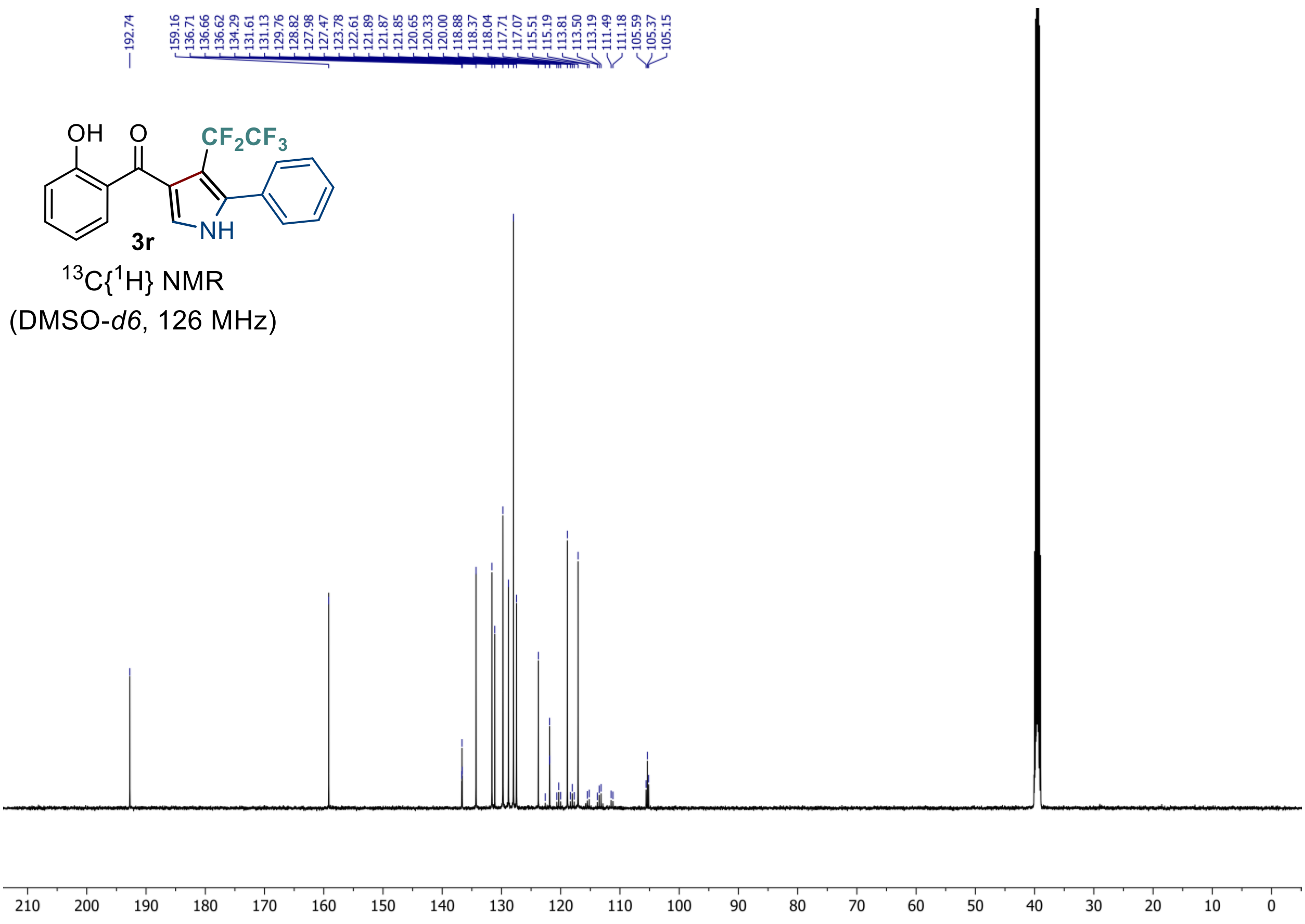

CF2CF3
OH
O
NH
3r
13C{1H} NMR
(DMSO-d6, 126 MHz)

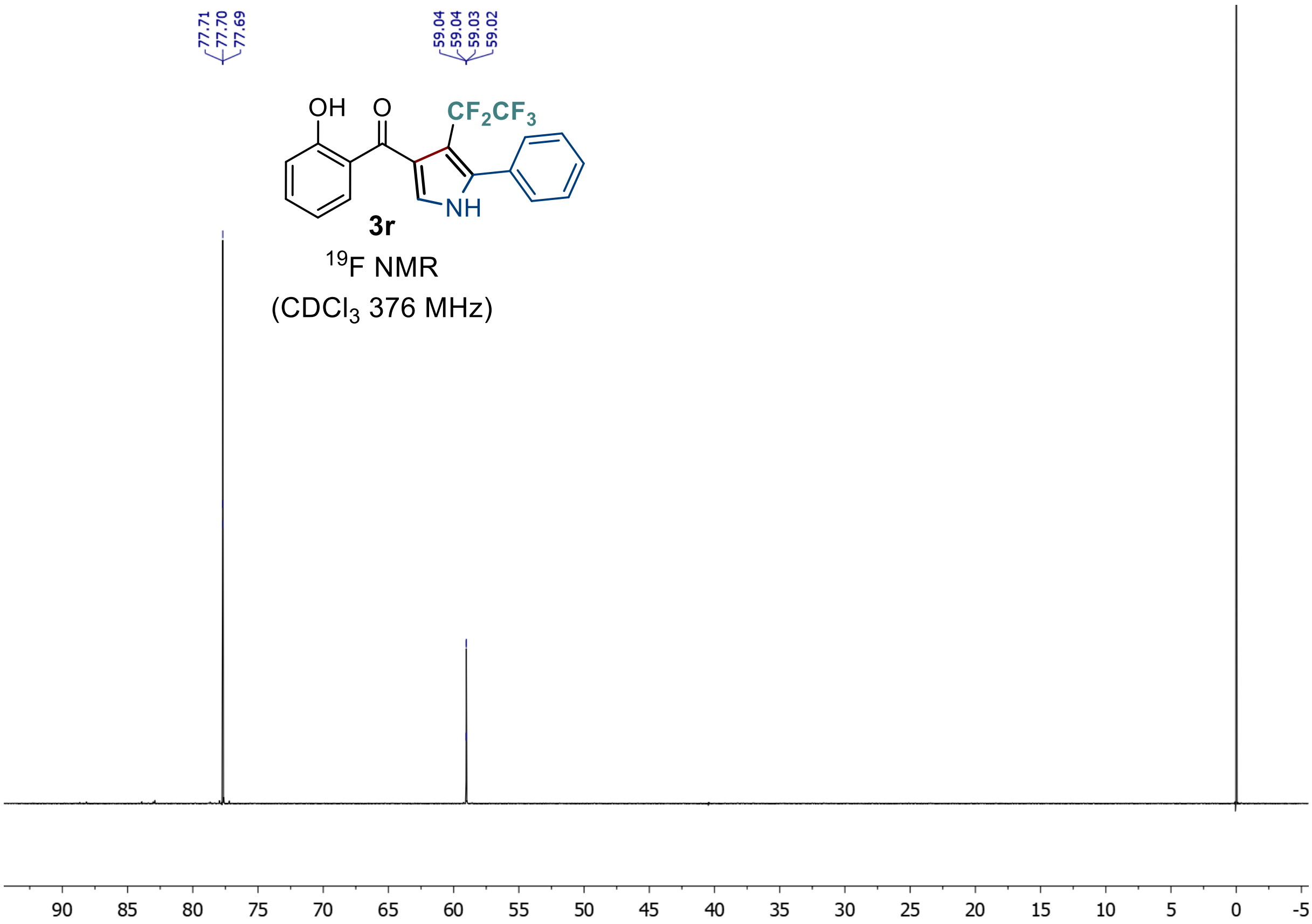

77.71
77.70
77.69
59.04
59.04
59.03
59.02
OH
O
CF2CF3
NH
3r
19F NMR
(CDCl3 376 MHz)
90 85 80 75 70 65 60 55 50 45 40 35 30 25 20 15 10 5 0 -5

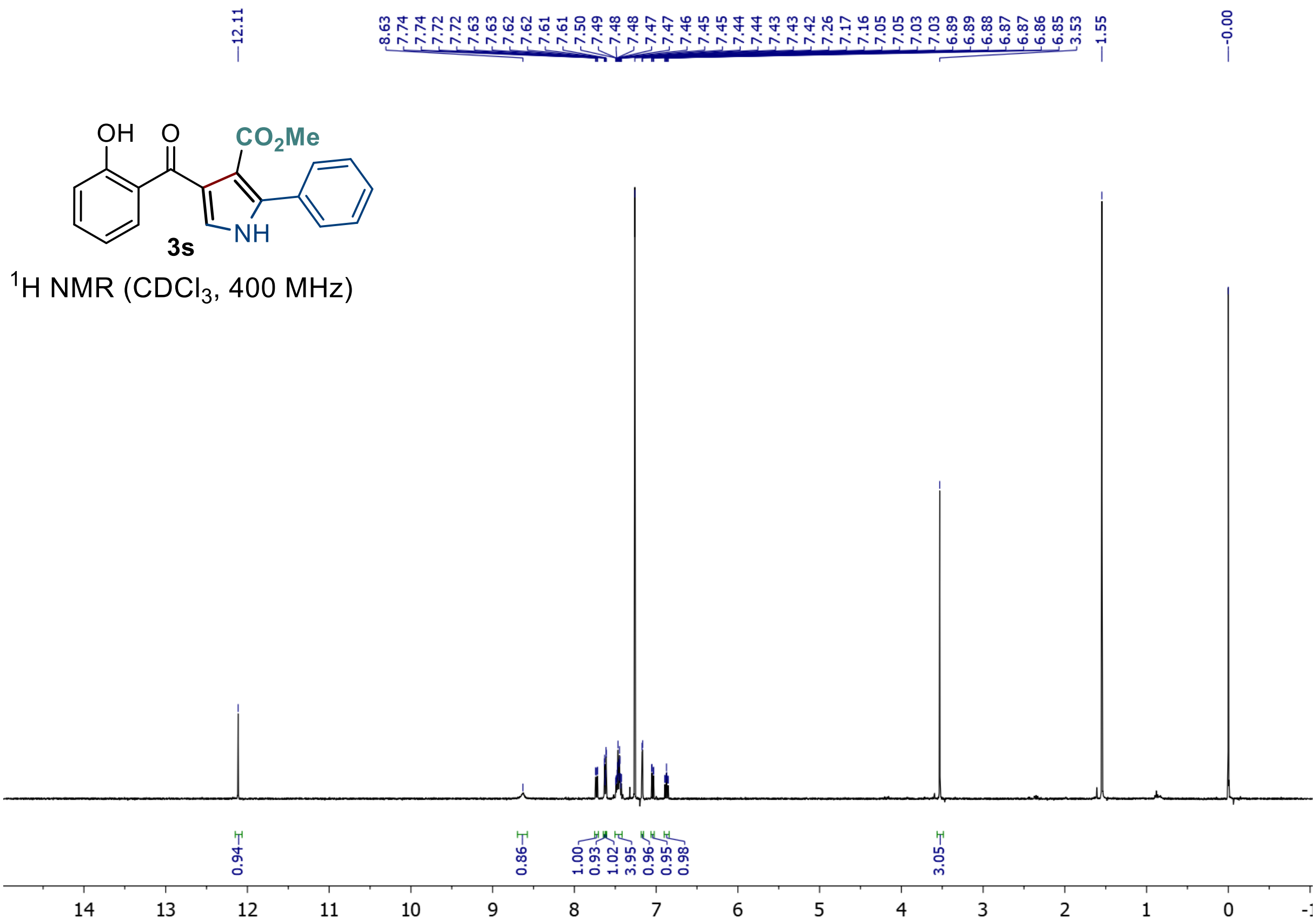
OH
O
CO2Me
NH
3s
1H NMR (CDCl3, 400 MHz)

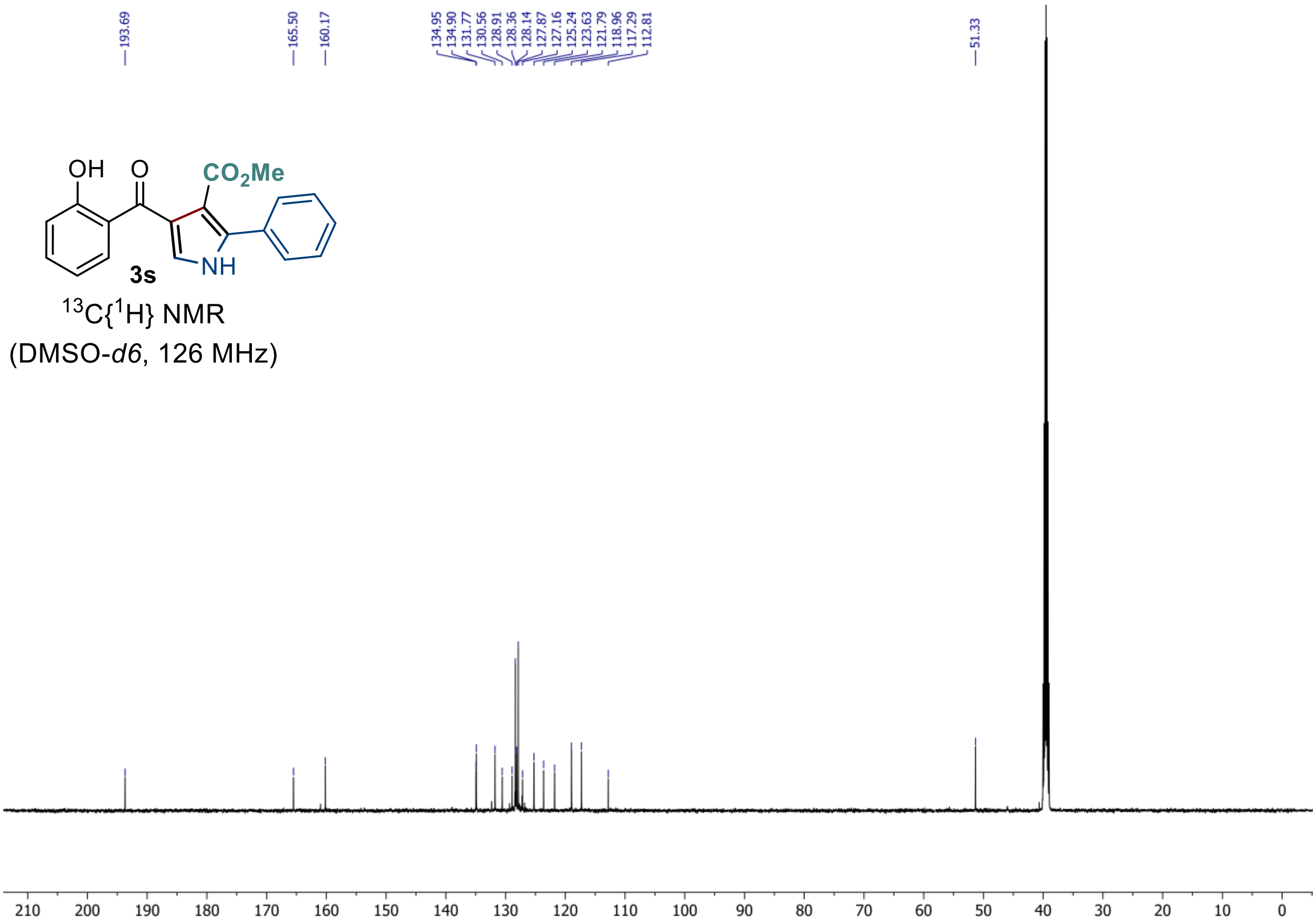
OH
O
CO2Me
NH
3s
13C{1H} NMR
(DMSO-d6, 126 MHz)

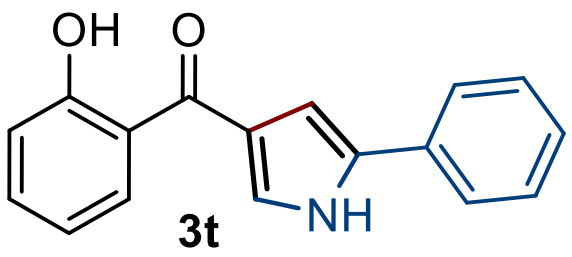

3t

^{1}H NMR ($CDCl_3$, 500 MHz)

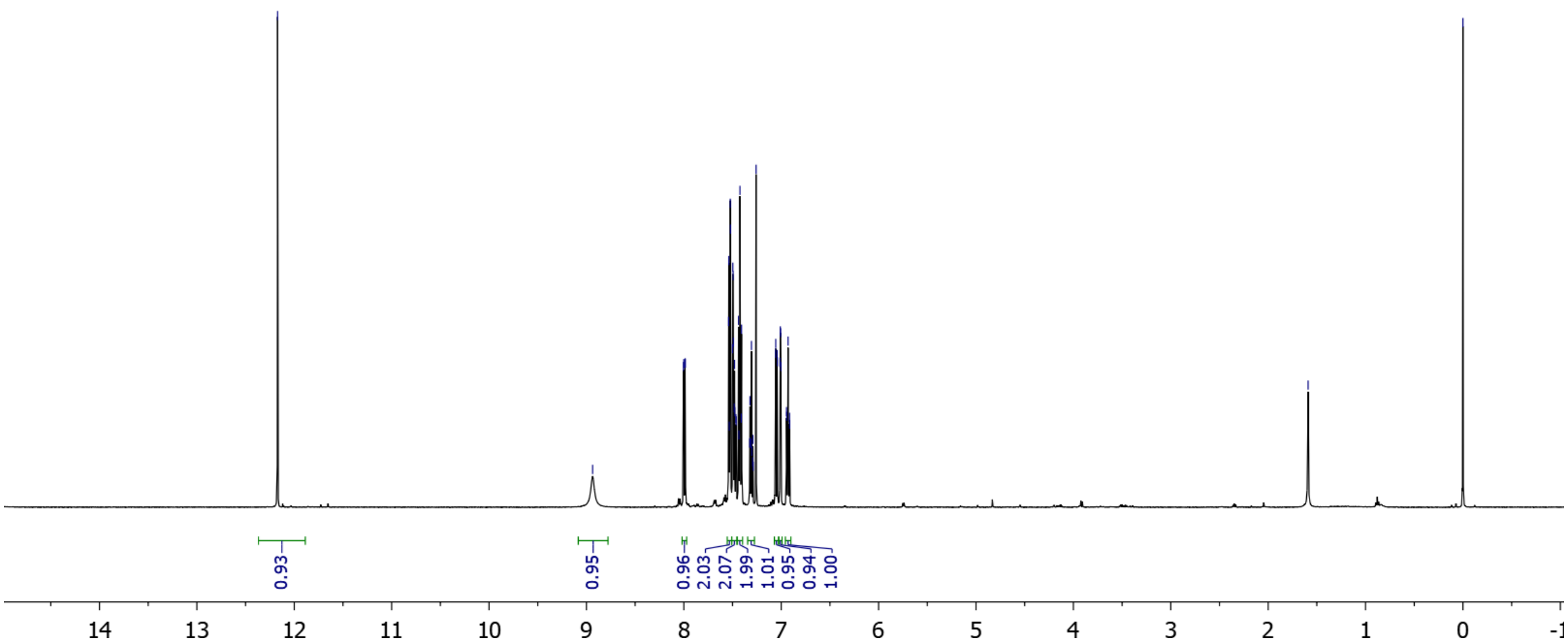

3. Copy of ^{1}H, ^{13}C NMR, ^{19}F and HRMS spectrum of pyrroles 3u and 4u

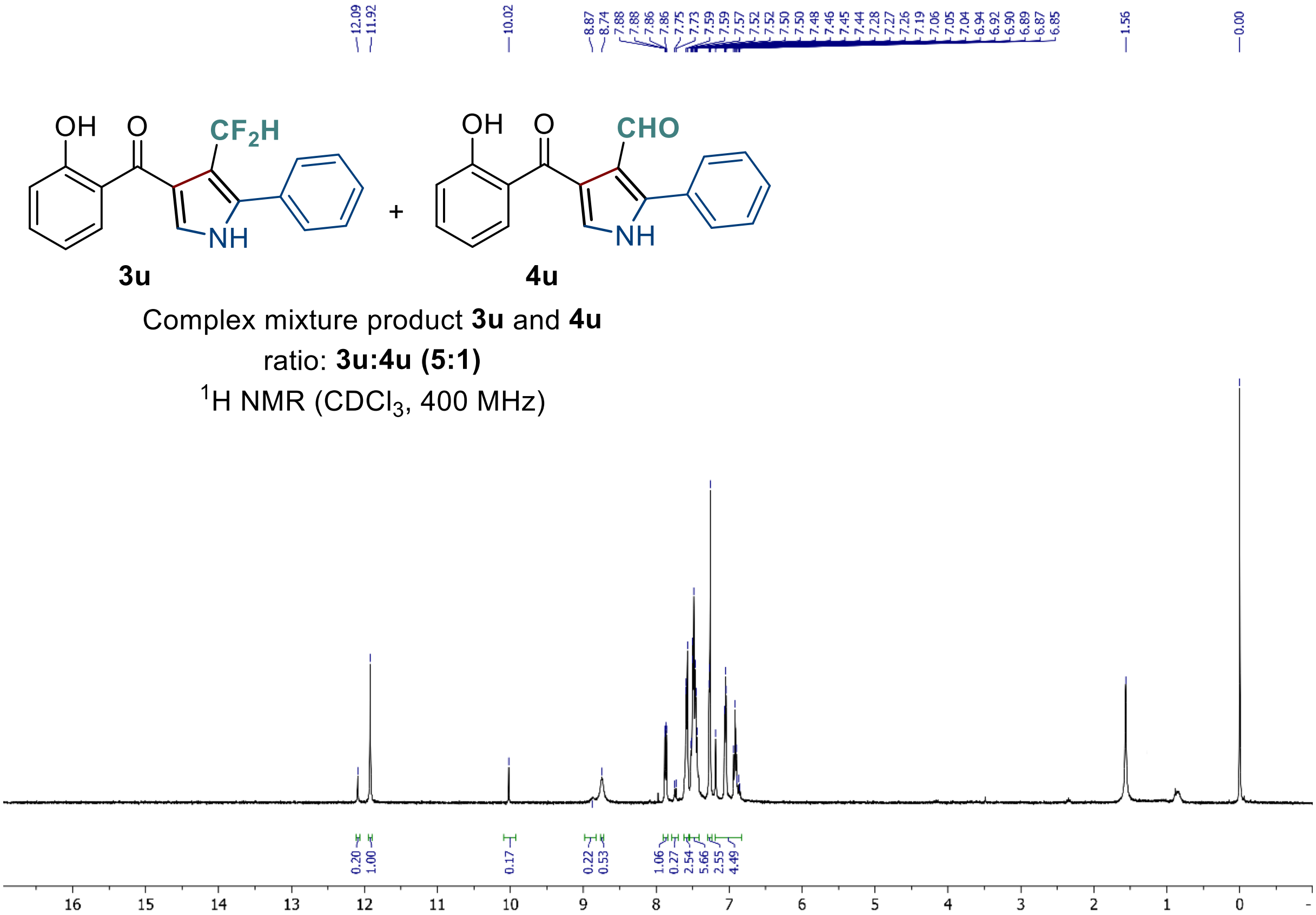

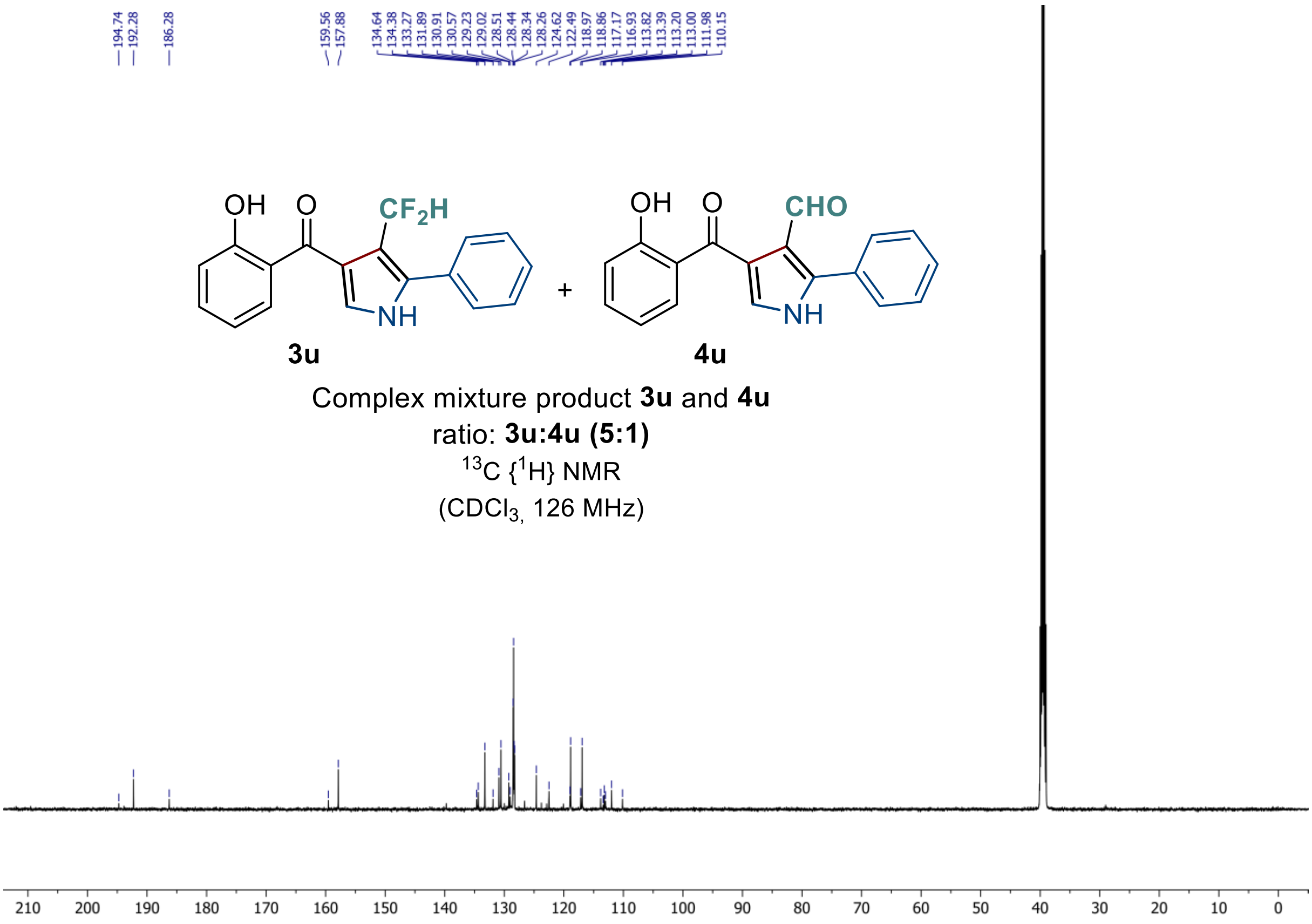

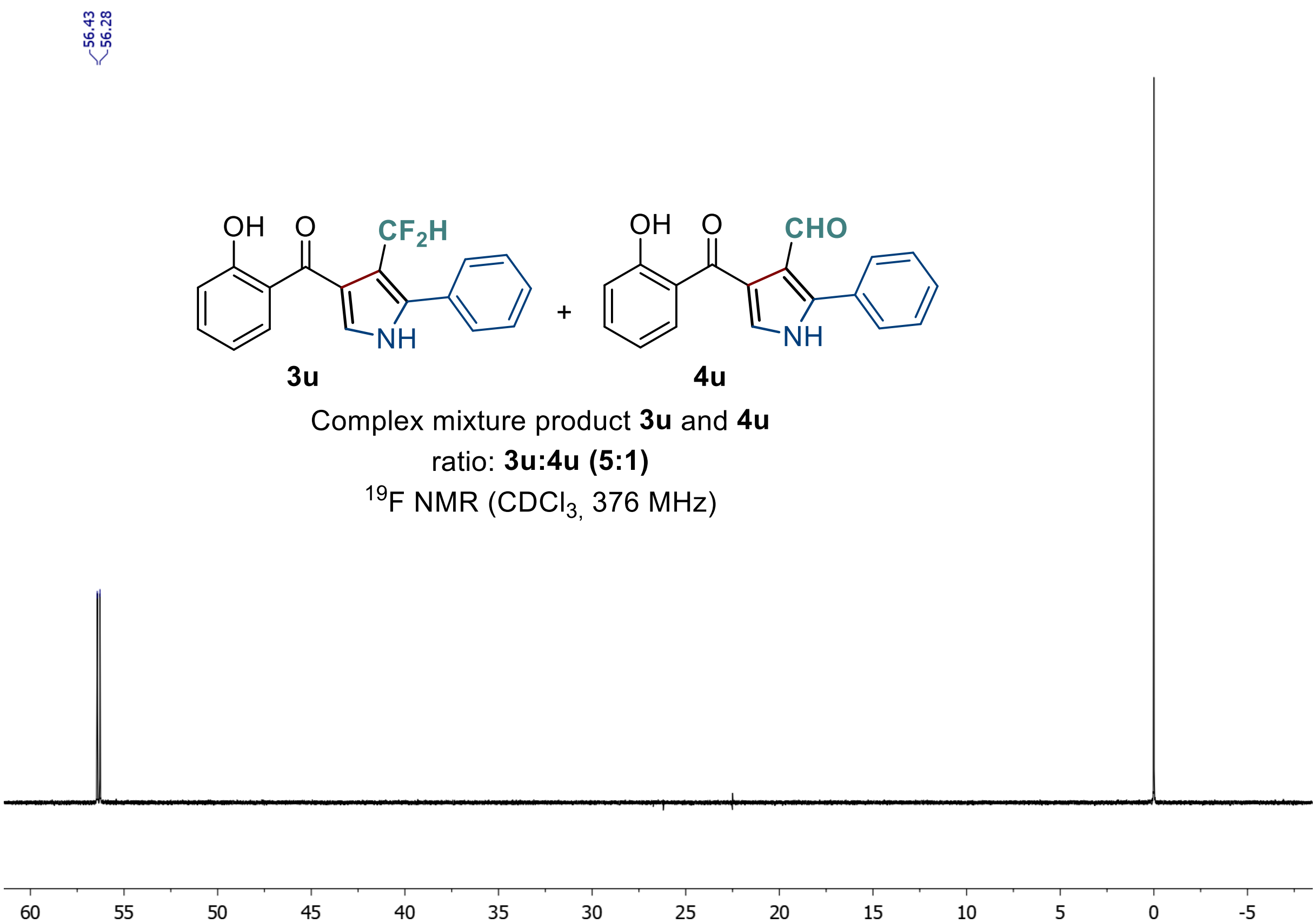

Complex mixture product **3u** and **4u**

ratio: **3u:4u (5:1)**

^{19}F NMR (CDCl$_3$, 376 MHz)

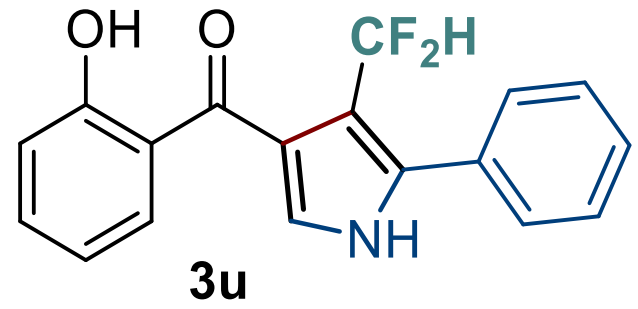

3u

Chemical Formula: $C_{18}H_{13}F_2NO_2$

Exact Mass: 313,0914

Meas. m/z	#	Ion Formula	m/z	err [ppm]
336.0807	1	$C_{18}H_{13}F_2NNaO_2$ $[M+Na]^+$	336.0807	-0.1
352.0544	2	$C_{18}H_{13}F_2KNO_2$ $[M+K]^+$	352.0546	0.5

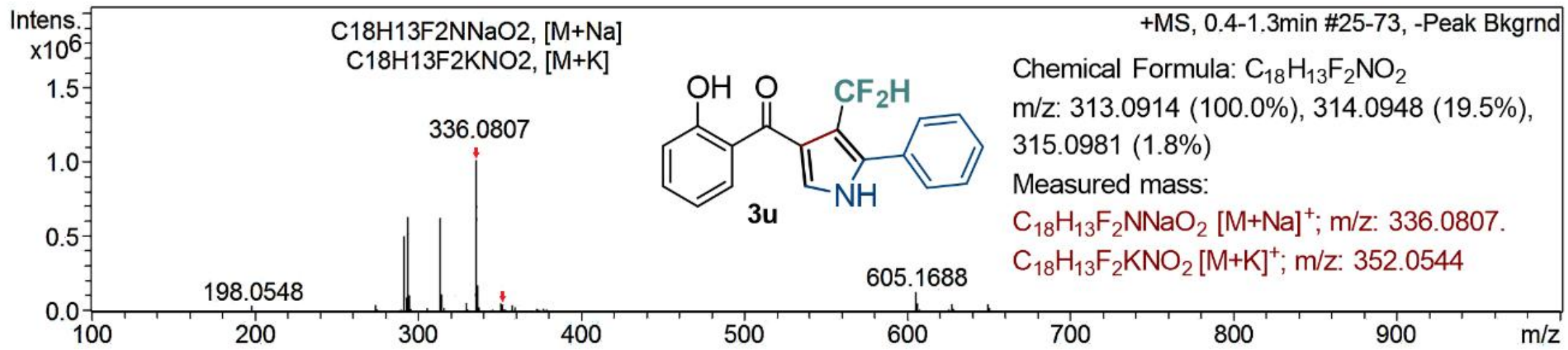

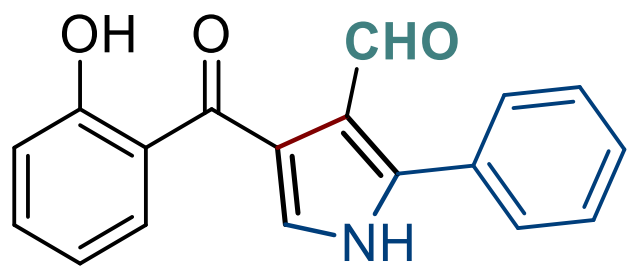

4u

Chemical Formula: $C_{18}H_{13}NO_3$

Exact Mass: 291,0895

Meas. m/z	#	Ion Formula	m/z	err [ppm]
292.0969	1	$C_{18}H_{14}NO_3$ $[M+H]^+$	292.0968	-0.3
314.0790	2	$C_{18}H_{13}NNaO_3$ $[M+Na]^+$	314.0788	-0.6
330/0527	3	$C_{18}H_{13}KNO_3$ $[M+K]^+$	330.0527	0.0

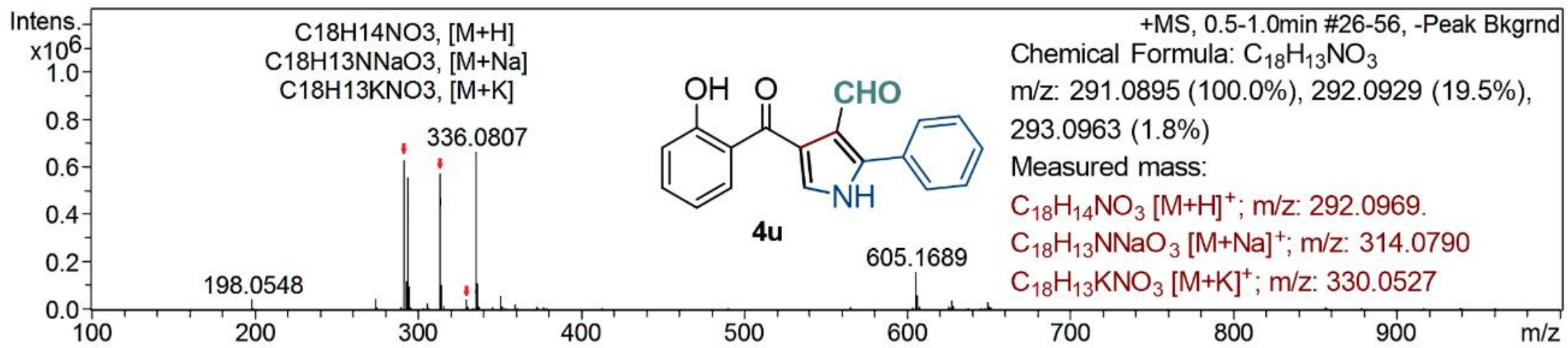

4. Copy of ^{1}H, ^{13}C and ^{19}F NMR spectrum of pyrroles 6a-d

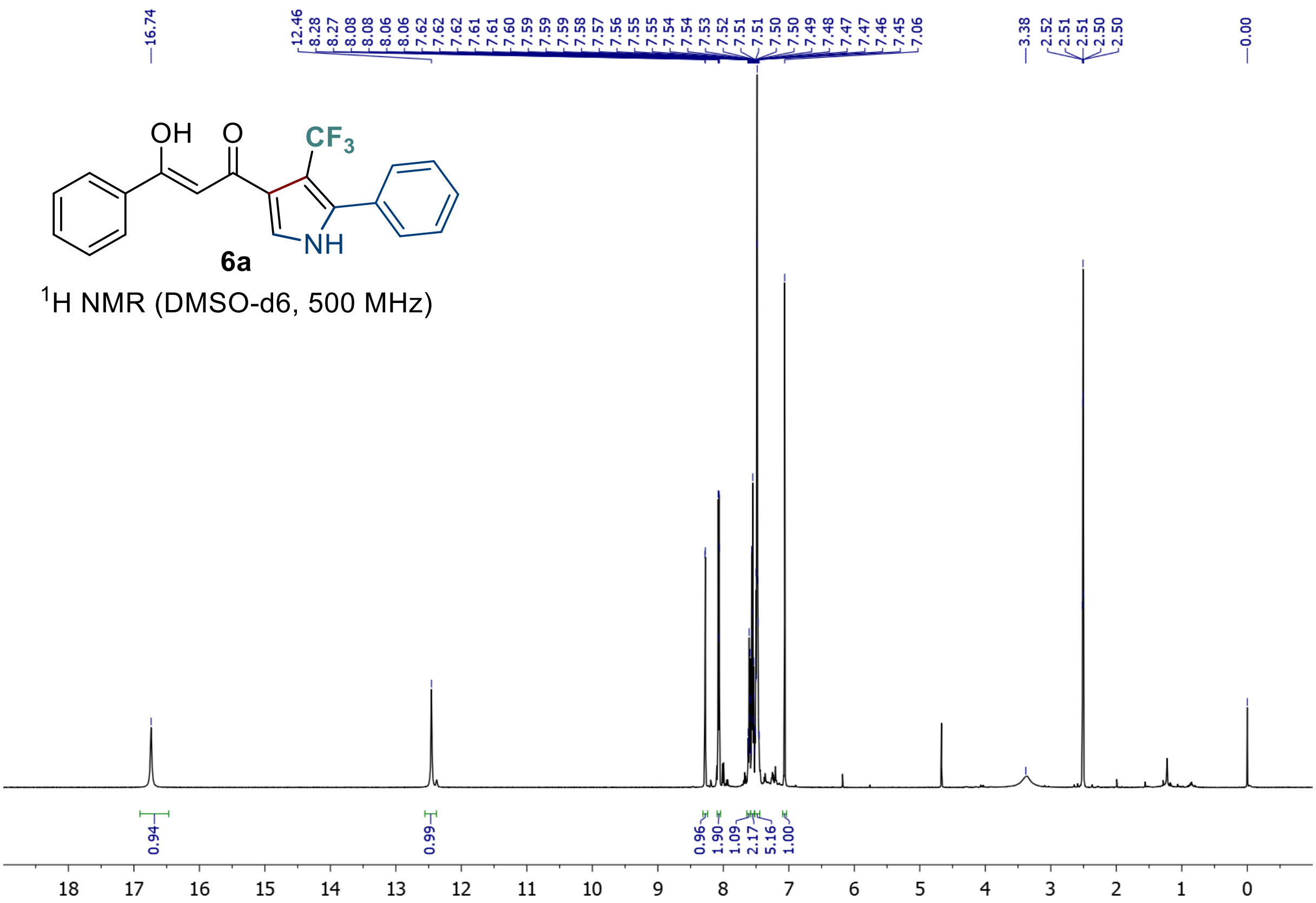

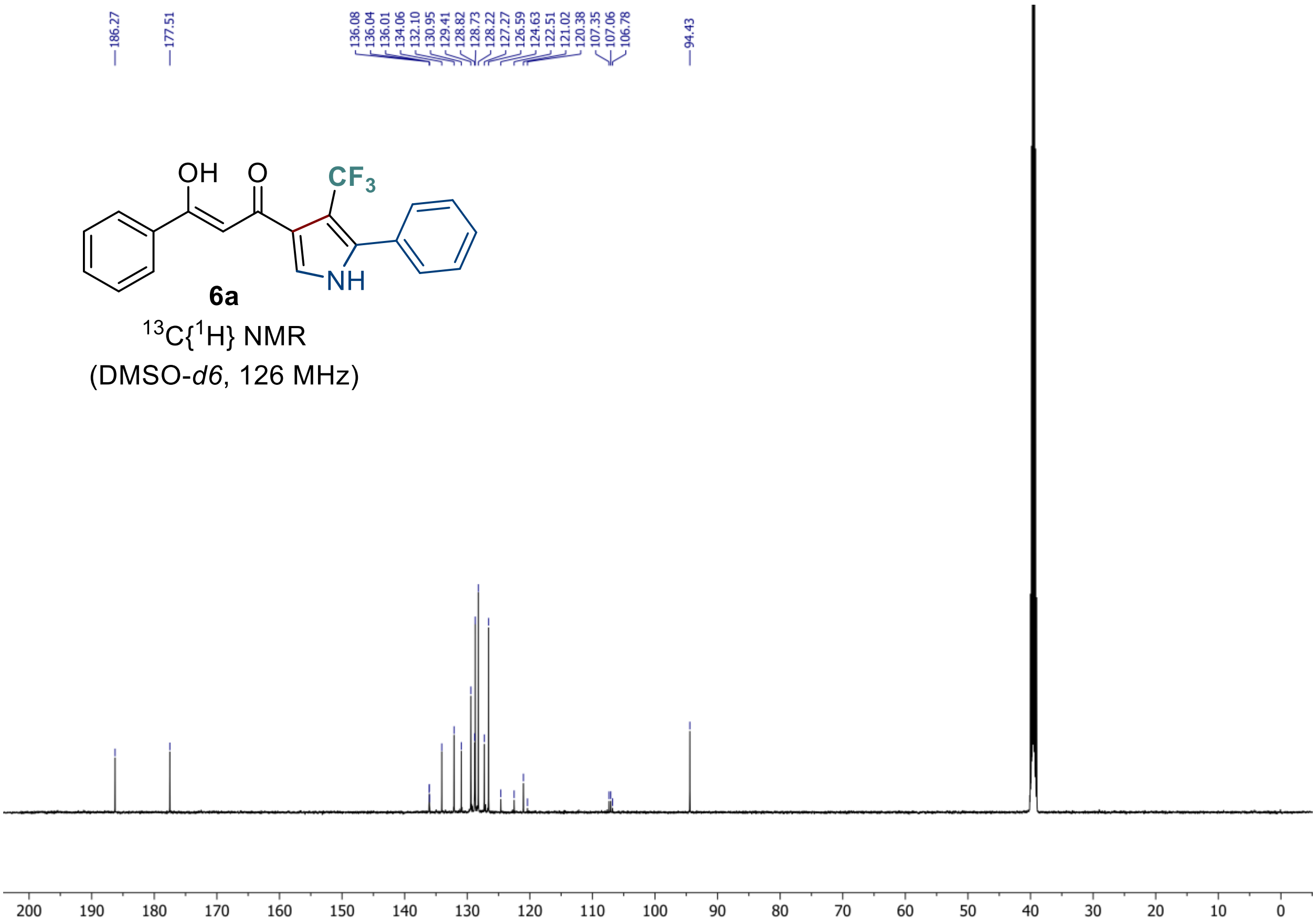

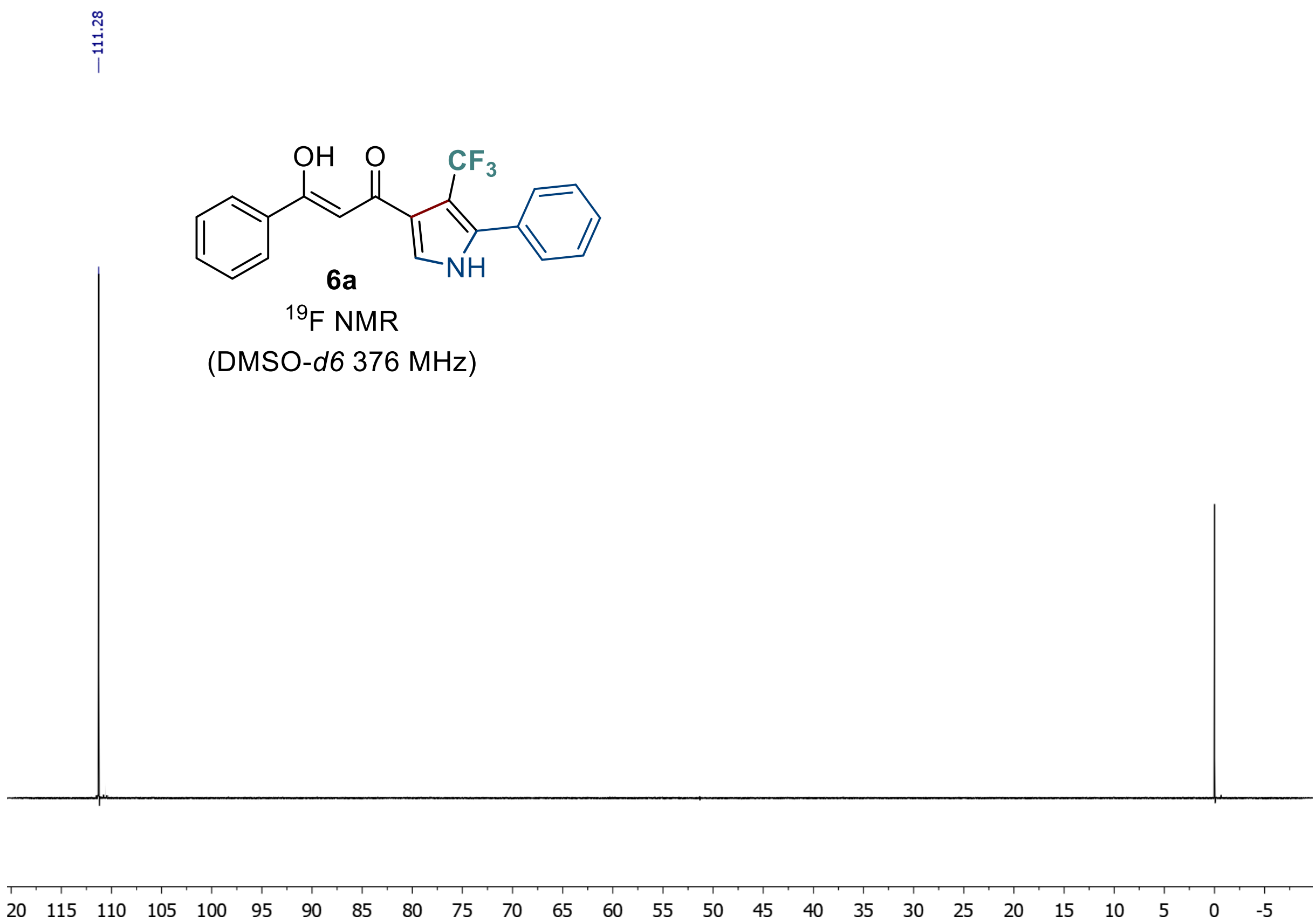
—111.28
OH
O
CF3
NH
6a
19F NMR
(DMSO-d6 376 MHz)
20 115 110 105 100 95 90 85 80 75 70 65 60 55 50 45 40 35 30 25 20 15 10 5 0 -5

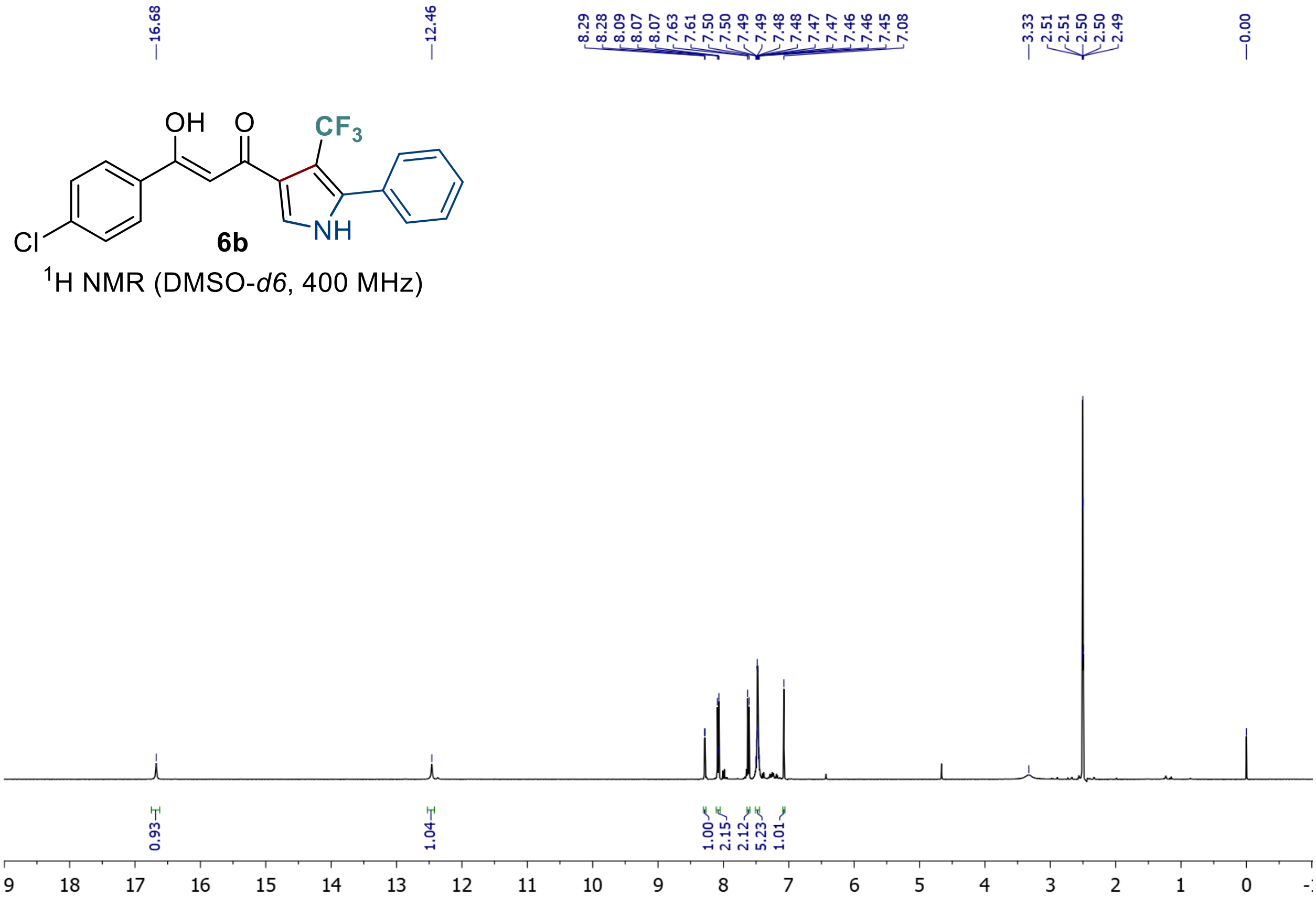

16.68
12.46
8.29 8.28 8.09 8.07 7.63 7.61 7.50 7.50 7.49 7.49 7.48 7.48 7.47 7.47 7.46 7.46 7.45 7.08
3.33 2.51 2.51 2.50 2.50 2.49
0.00
OH
O
CF3
Cl
6b
NH
1H NMR (DMSO-d6, 400 MHz)
0.93
1.04
1.00 2.15 2.12 5.23 1.01

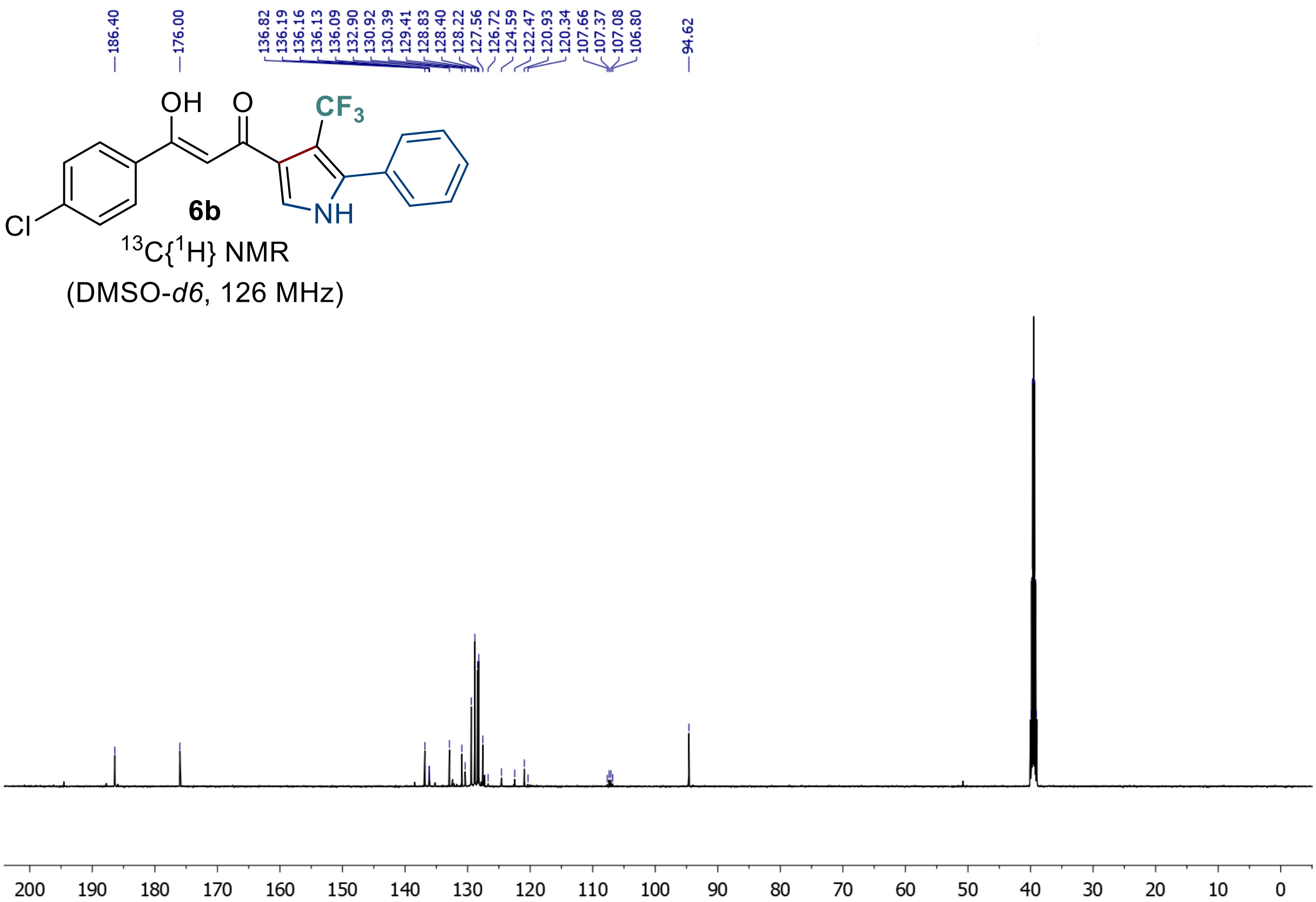

186.40
176.00
136.82 136.19 136.16 136.13 136.09 132.90 132.92 130.92 130.39 129.41 128.83 128.40 128.22 127.56 126.72 124.59 122.47 120.93 120.34 107.66 107.37 107.08 106.80
94.62
OH
O
CF3
Cl
6b
NH
13C{1H} NMR
(DMSO-d6, 126 MHz)

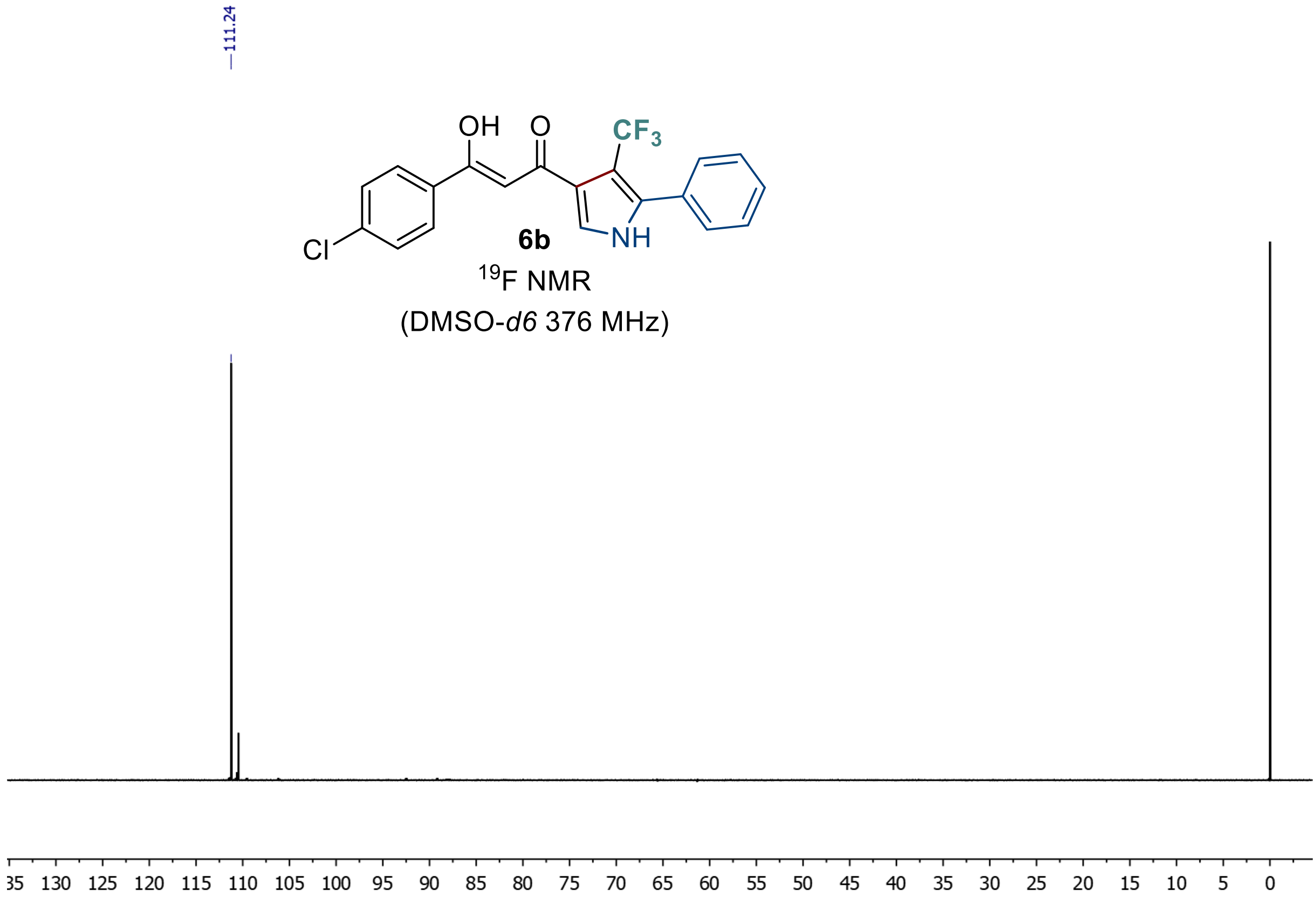
—111.24
OH
O
CF3
Cl
NH
6b
19F NMR
(DMSO-d6 376 MHz)
35 130 125 120 115 110 105 100 95 90 85 80 75 70 65 60 55 50 45 40 35 30 25 20 15 10 5 0

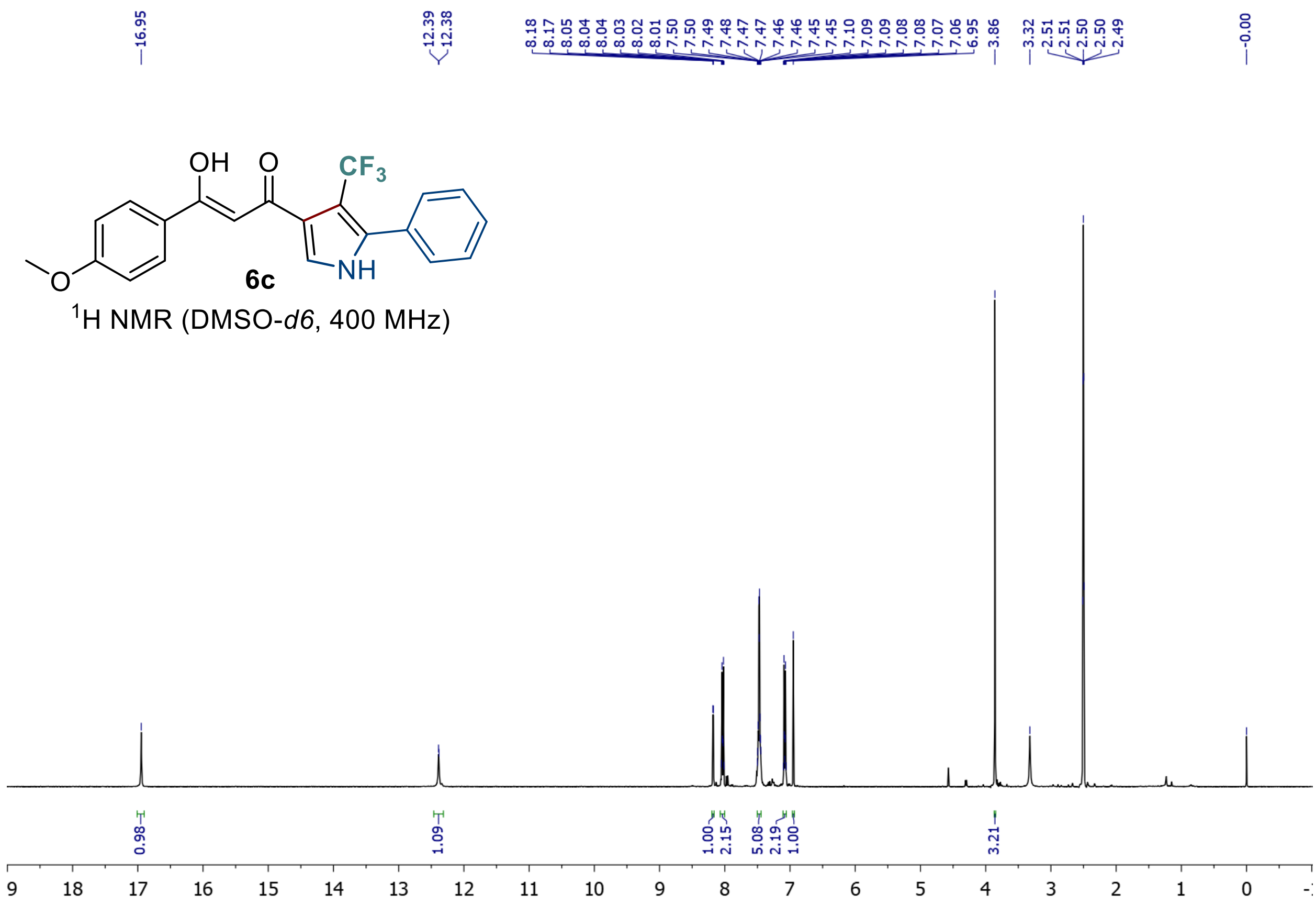
16.95
12.39
12.38
8.18 8.17 8.05 8.04 8.04 8.03 8.02 8.01 7.50 7.50 7.49 7.48 7.47 7.47 7.46 7.46 7.45 7.45 7.10 7.09 7.09 7.08 7.08 7.07 7.06 6.95
3.86
3.32 2.51 2.51 2.50 2.50 2.49
-0.00
OH
O
CF3
NH
6c
1H NMR (DMSO-d6, 400 MHz)
0.98
1.09
1.00 2.15 5.08 2.19 1.00
3.21

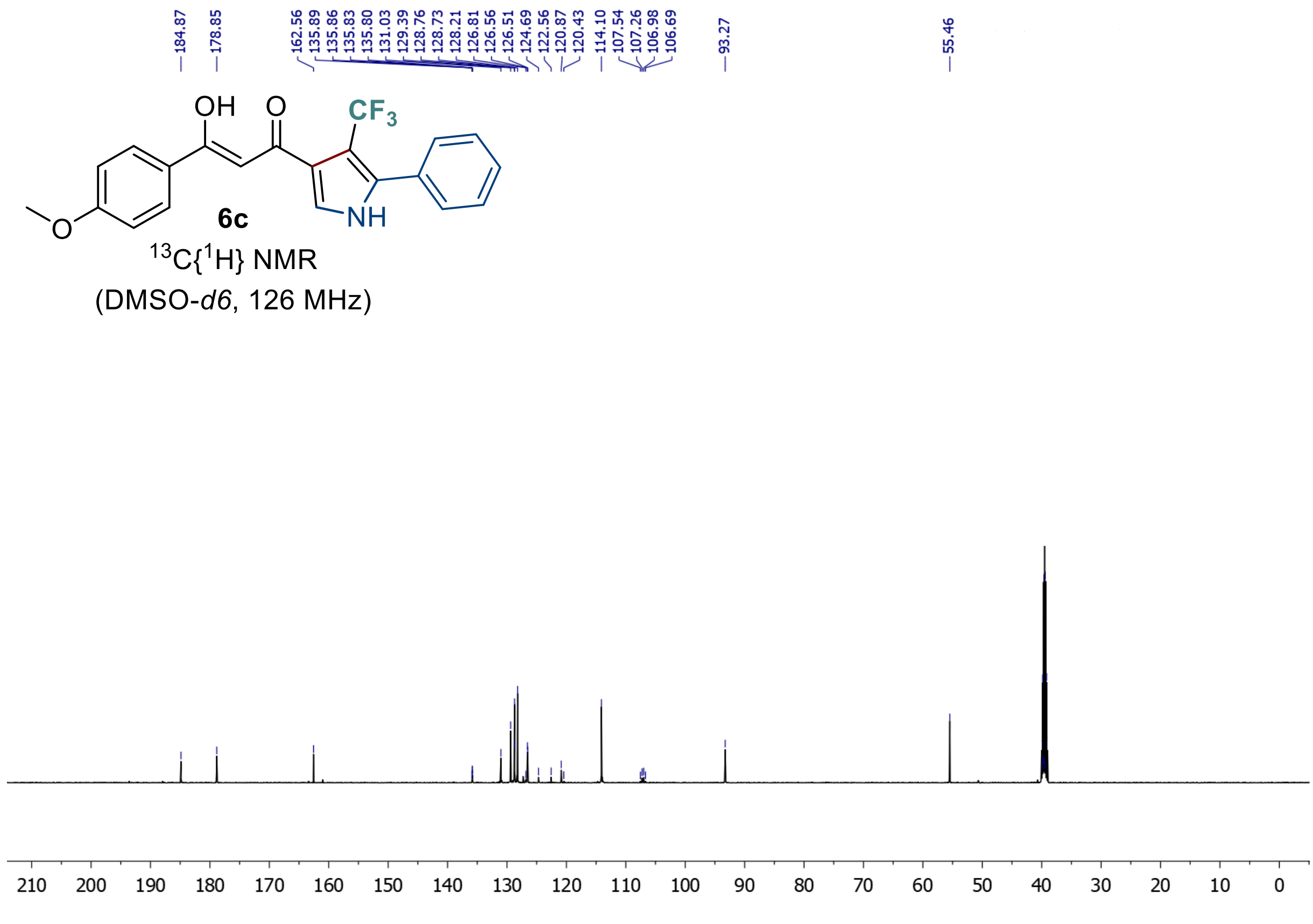
184.87
178.85
162.56 135.89 135.86 135.83 135.80 131.03 129.39 128.76 128.73 128.21 126.81 126.56 126.51 124.69 122.56 120.87 120.43
114.10 107.54 107.26 106.98 106.69
93.27
55.46
OH
O
CF3
NH
6c
13C{1H} NMR
(DMSO-d6, 126 MHz)

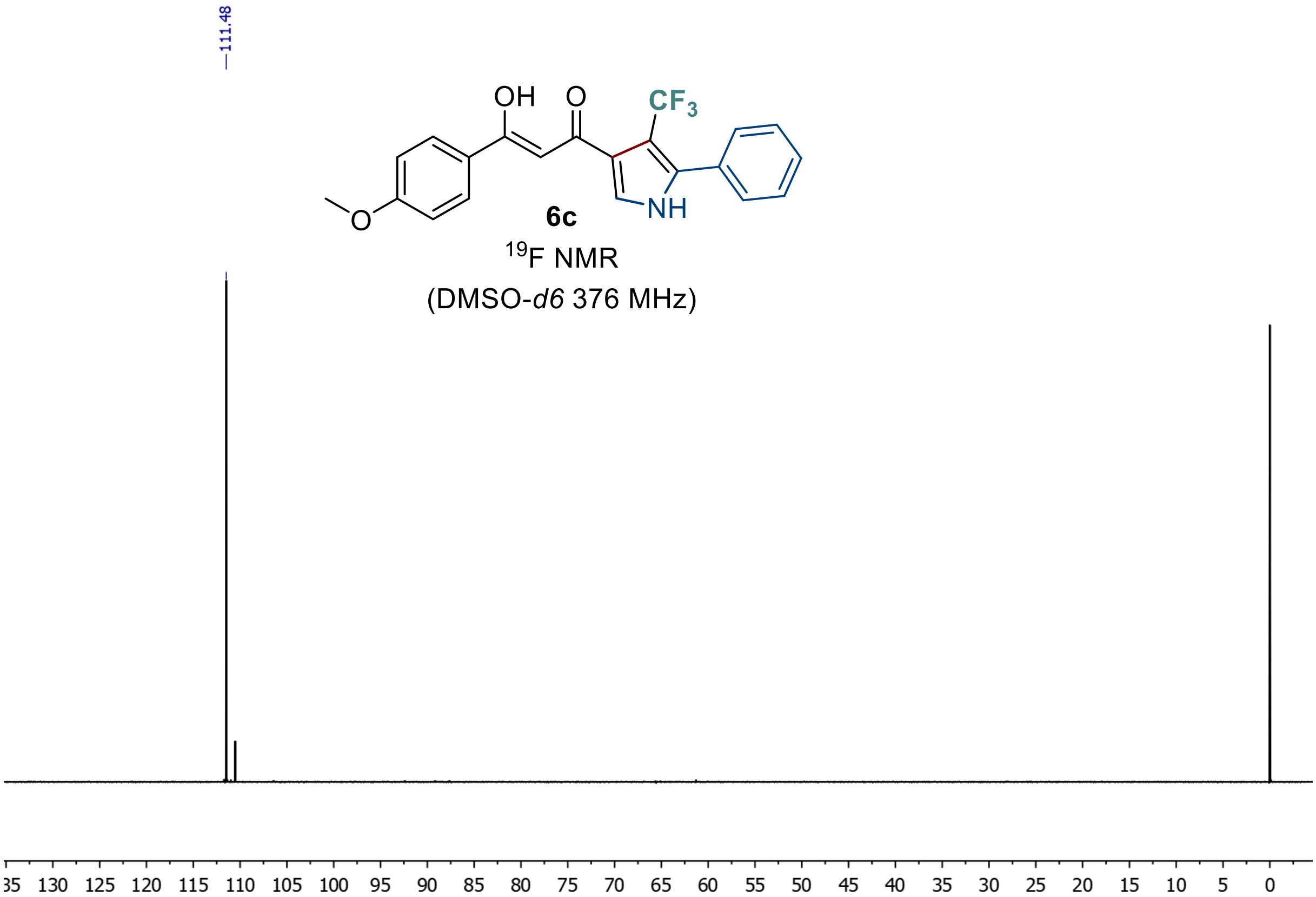
—111.48
OH
O
CF3
O
NH
6c
19F NMR
(DMSO-d6 376 MHz)
35 130 125 120 115 110 105 100 95 90 85 80 75 70 65 60 55 50 45 40 35 30 25 20 15 10 5 0

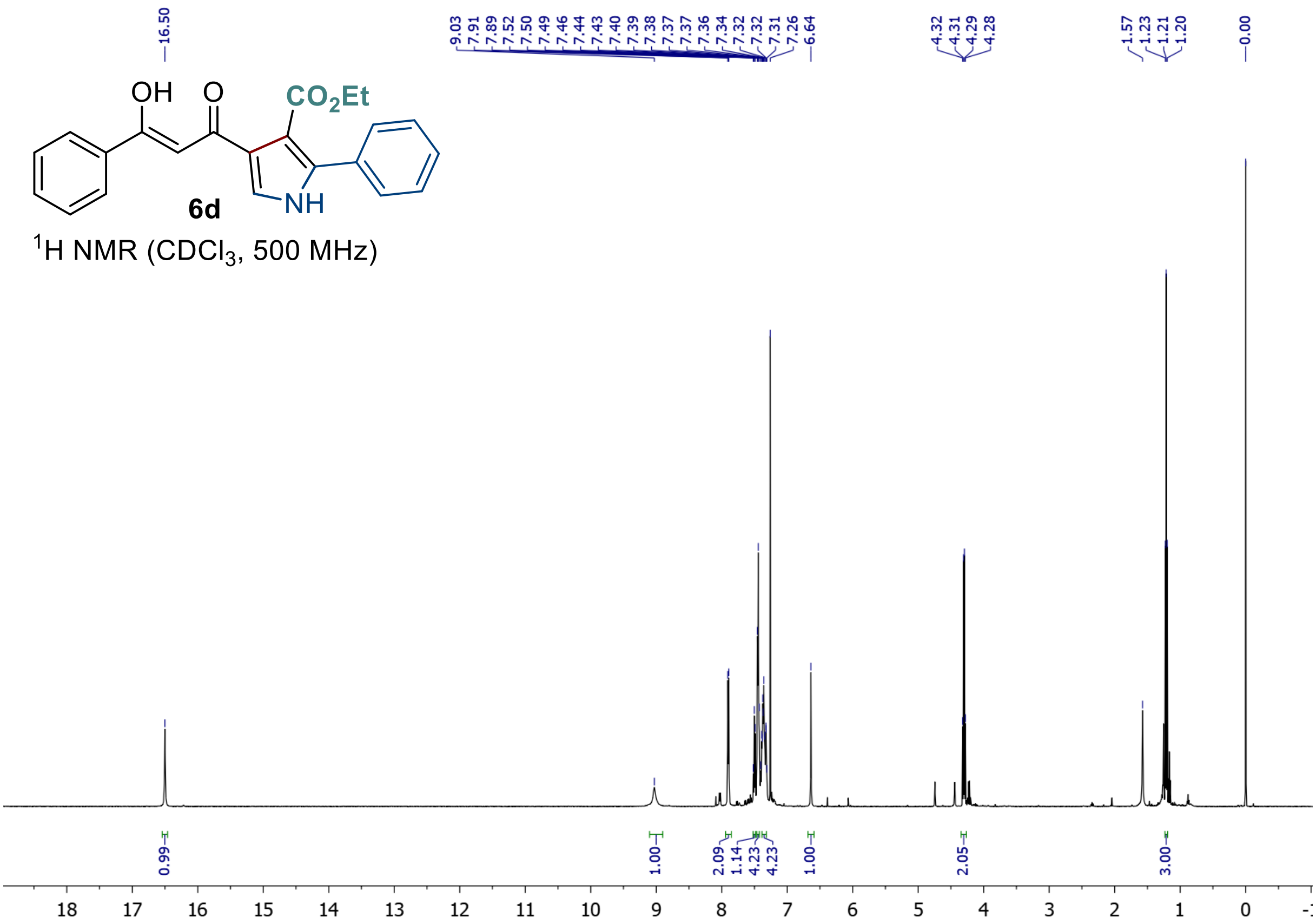
16.50
9.03 7.91 7.89 7.52 7.50 7.49 7.46 7.44 7.43 7.40 7.39 7.38 7.37 7.37 7.36 7.34 7.32 7.32 7.31 7.26 6.64
4.32 4.31 4.29 4.28
1.57 1.23 1.21 1.20
0.00
OH O
CO2Et
NH
6d
1H NMR (CDCl3, 500 MHz)
0.99
1.00
2.09 1.14 4.23 4.23
1.00
2.05
3.00
18 17 16 15 14 13 12 11 10 9 8 7 6 5 4 3 2 1 0

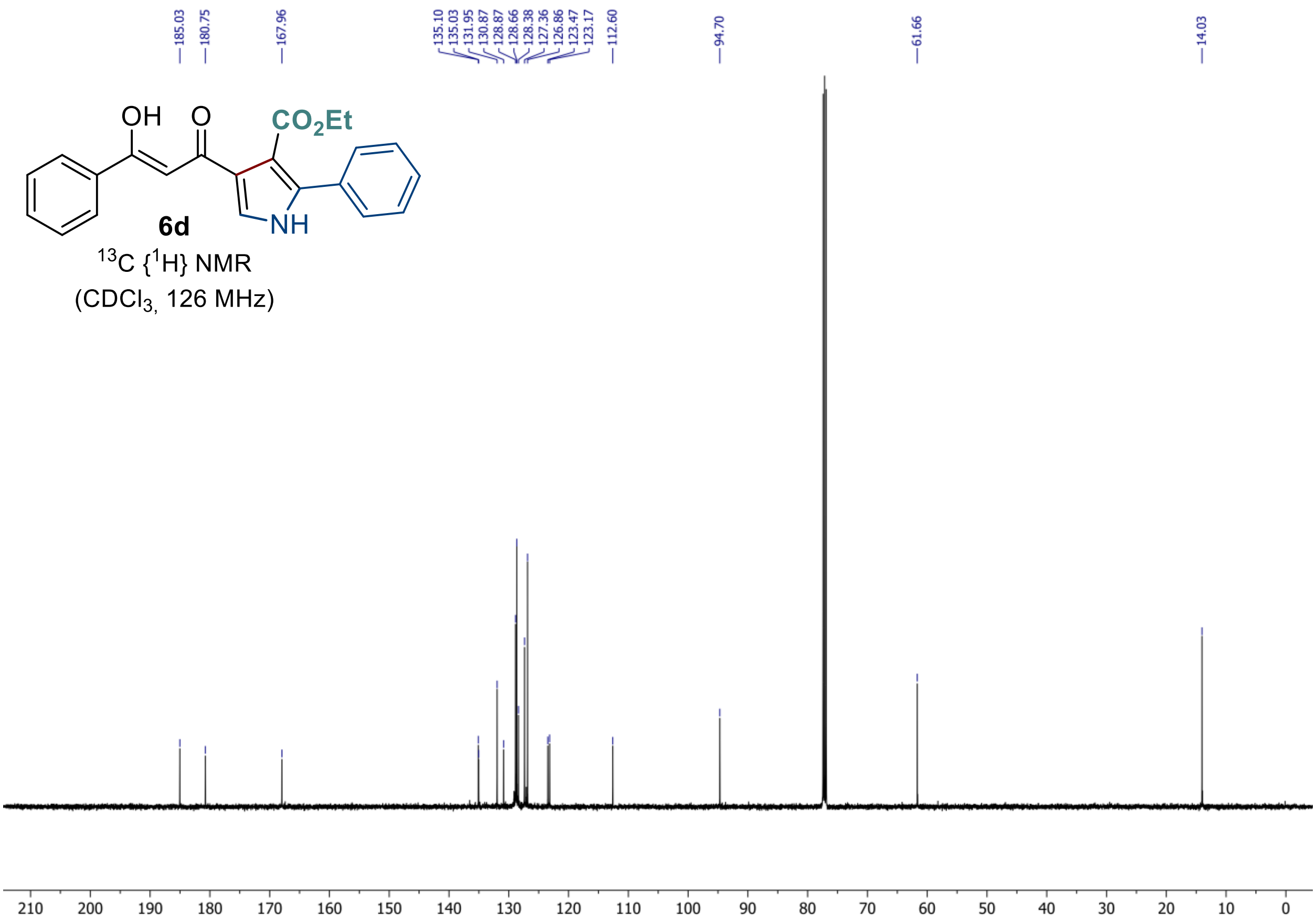
185.03 180.75
167.96
135.10 135.03 131.95 130.87 128.87 128.66 128.38 127.36 126.86 123.47 123.17
112.60
94.70
61.66
14.03
OH O
CO2Et
NH
6d
13C {1H} NMR
(CDCl3, 126 MHz)
210 200 190 180 170 160 150 140 130 120 110 100 90 80 70 60 50 40 30 20 10 0

5. X-ray crystal data for 3f

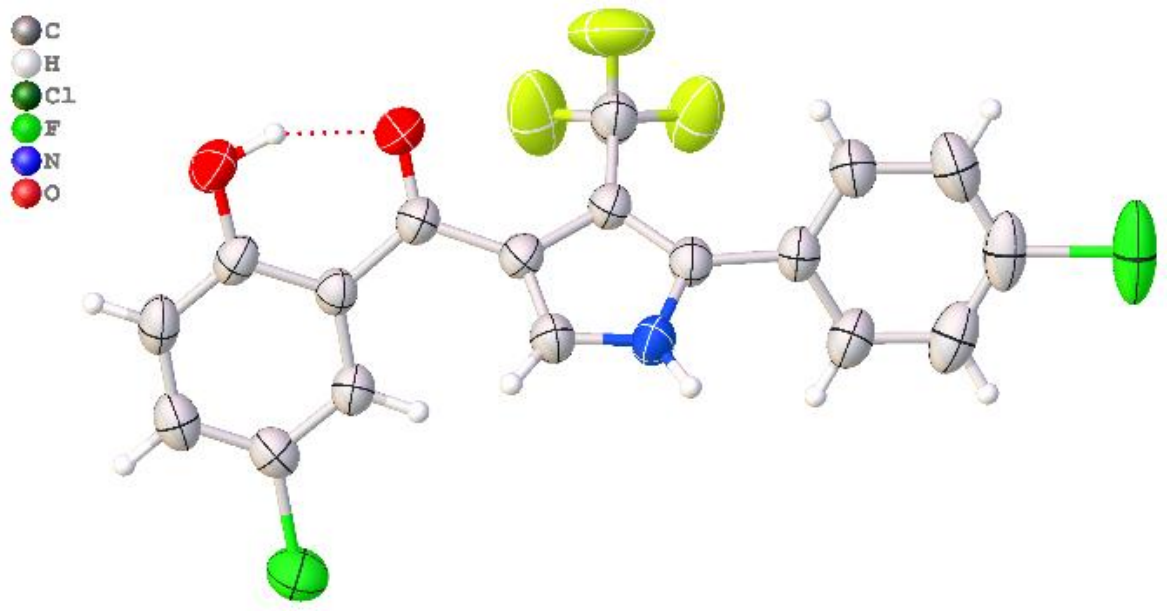

CCDC 2297092

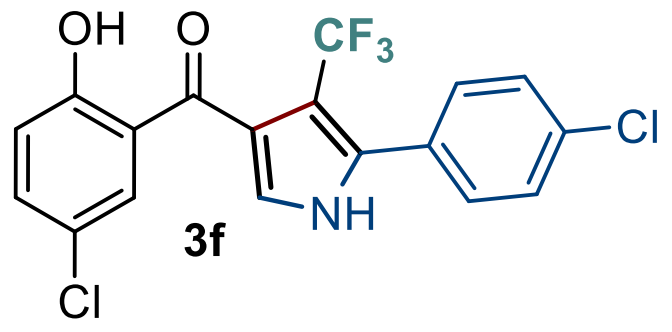

Table S1 Crystal data and structure refinement for 3f (MMX-02-976) / CCDC 2297092.

Identification code	MMX-02-976
Empirical formula	$C_{18}H_{10}Cl_2F_3NO_2$
Formula weight	400.17
Temperature/K	295(2)
Crystal system	monoclinic
Space group	$P2_1/n$
a/Å, b/Å, c/Å	7.1089(6), 16.9445(12), 14.1791(12)
α/° β/° γ/°	90, 97.778(9), 90
Volume/$Å^3$	1692.3(2)
Z	4
$\rho_{calc}g/cm^3$	1.571
μ/mm^{-1}	0.427
F(000)	808.0
Crystal size/mm^3	0.48 × 0.39 × 0.31
Radiation	MoKα (λ = 0.71073)
2Θ range for data collection/°	7.226 to 60.95
Index ranges	$-9 \leq h \leq 9$, $-24 \leq k \leq 21$, $-19 \leq l \leq 18$
Reflections collected	13230
Independent reflections	4663 [R_{int} = 0.0540, R_{sigma} = 0.0678]
Data/restraints/parameters	4663/0/243
Goodness-of-fit on F^2	1.030
Final R indexes [I>=2σ (I)]	R_1 = 0.0659, wR_2 = 0.1581
Final R indexes [all data]	R_1 = 0.1366, wR_2 = 0.2186
Largest diff. peak/hole / e $Å^{-3}$	0.33/-0.58

Crystal Data for $C_{18}H_{10}Cl_2F_3NO_2$ (M =400.17 g/mol): monoclinic, space group $P2_1/n$ (no. 14), a = 7.1089(6) Å, b = 16.9445(12) Å, c = 14.1791(12) Å, β = 97.778(9)°, V = 1692.3(2) $Å^3$, Z = 4, T = 295(2) K, μ(MoKα) = 0.427 mm^{-1}, $Dcalc$ = 1.571 g/cm^3, 13230 reflections measured (7.226° ≤ 2Θ ≤ 60.95°), 4663 unique (R_{int} = 0.0540, R_{sigma} = 0.0678) which were used in all calculations. The final R_1 was 0.0659 (I > 2σ(I)) and wR_2 was 0.2186 (all data).

6. Citing literature

1. Sosnovskikh, V. Y., Usachev, B. I.; *Synthesis*, **2002**, 2002 (16), 2341–2343. 10.1055/s-2002-35233.
2. Sosnovskikh, V. Y., Usachev, B. I., Sizov, A. Y., Barabanov, M. A.; *Synthesis*, **2004**, 6, 942–948. 10.1055/s-2004-822321.
3. Kochnev, I. A., Zavyalova, L. S., Avkhadieva, A. I., Tverdokhlebov, N. A., Barkov, A. Y.; *J. Fluor. Chem.*, **2024**, *280*, 110368. 10.1016/j.jfluchem.2024.110368
4. Kochnev, I. A., Barkov, A. Y.; *New J. Chem.*, **2025**, *49(46)*, 20254–20263. 10.1039/D5NJ03333K.
5. Qi, X., Xiang, H., Yang, Y., Yang, C.; *RSC Adv.*, **2015**, *5(119)*, 98549–98552. 10.1039/C5RA21915A.
